# Advanced Oxidation Protein Products Are Strongly Associated with the Serum Levels and Lipid Contents of Lipoprotein Subclasses in Healthy Volunteers and Patients with Metabolic Syndrome

**DOI:** 10.3390/antiox13030339

**Published:** 2024-03-11

**Authors:** Iva Klobučar, Lidija Hofmann, Hansjörg Habisch, Margarete Lechleitner, Lucija Klobučar, Matias Trbušić, Gudrun Pregartner, Andrea Berghold, Tobias Madl, Saša Frank, Vesna Degoricija

**Affiliations:** 1 Department of Cardiology, Sisters of Charity University Hospital Centre, 10000 Zagreb, Croatia; iva.klobucar@gmail.com (I.K.); matias.trbusic@gmail.com (M.T.); 2 Institute of Biomedical Science, Department of Health Studies, FH JOANNEUM University of Applied Sciences, 8020 Graz, Austria; lidija.hofmann@fh-joanneum.at; 3 Otto Loewi Research Center, Medicinal Chemistry, Medical University of Graz, 8010 Graz, Austria; hansjoerg.habisch@medunigraz.at (H.H.); tobias.madl@medunigraz.at (T.M.); 4 Gottfried Schatz Research Center, Molecular Biology and Biochemistry, Medical University of Graz, 8010 Graz, Austria; margarete.lechleitner@medunigraz.at; 5 Department of Medicine, University Hospital Centre Osijek, 31000 Osijek, Croatia; klobucar.lucija@gmail.com; 6 School of Medicine, University of Zagreb, 10000 Zagreb, Croatia; vesna.degoricija@mef.hr; 7 Institute for Medical Informatics, Statistics und Documentation, Medical University of Graz, 8036 Graz, Austria; gudrun.pregartner@medunigraz.at (G.P.); andrea.berghold@medunigraz.at (A.B.); 8 BioTechMed-Graz, 8010 Graz, Austria; 9 Department of Medicine, Sisters of Charity University Hospital Centre, 10000 Zagreb, Croatia

**Keywords:** NMR spectroscopy, lipoprotein profiling, advanced oxidation protein products, metabolic syndrome

## Abstract

The association between advanced oxidation protein products (AOPPs) and lipoprotein subclasses remains unexplored. Therefore, we performed comprehensive lipoprotein profiling of serum using NMR spectroscopy and examined the associations of lipoprotein subclasses with the serum levels of AOPPs in healthy volunteers (HVs) and patients with metabolic syndrome (MS). The serum levels of AOPPs were significantly positively correlated with the serum levels of very-low-density lipoprotein (VLDL), intermediate-density lipoprotein (IDL), and low-density lipoprotein (LDL); however, they were significantly negatively correlated with high-density lipoprotein (HDL). These lipoproteins (and their subclasses) differed markedly regarding the direction of correlations between their lipid contents and AOPPs. The strength of the correlations and the relative contributions of the subclasses to the correlations were different in the HVs and patients with MS. As revealed by orthogonal partial least squares discriminant analyses, the serum levels of IDL were strong determinants of AOPPs in the HVs, whereas the serum levels of VLDL and the lipid content of LDL were strong determinants in both groups. We conclude that IDL, VLDL, and LDL facilitate, whereas HDL diminishes the bioavailability of serum AOPPs. The presence of MS and the lipid contents of the subclasses affect the relationship between lipoproteins and AOPPs.

## 1. Introduction

Metabolic syndrome (MS) is a pathophysiological condition which comprises arterial hypertension, central obesity, dyslipidemia with increased serum triglycerides, and decreased HDL cholesterol (HDL-C), as well as insulin resistance and hyperglycemia [1]. Oxidative stress, a condition that arises from a disbalance between the generation and elimination of reactive oxygen species (ROS), has been shown to be implicated in the pathophysiology of MS [2,3,4,5]. In MS, a pathological hyperactivation of various enzymes, such as nicotinamide adenine dinucleotide phosphate (NADPH) oxidase, xanthine oxidase, endothelial nitric oxide synthase, and myeloperoxidase, as well as a hyperactivation and dysfunction of the mitochondria, causes an overproduction of ROS and, consequently, a modification of and damage to various biological molecules and cellular structures [6].

Several studies have highlighted that advanced oxidation protein products (AOPPs) are appropriate parameters to determine oxidative stress in MS [7,8,9]. AOPPs are dityrosine-, pentosidine-, and carbonyl-containing cross-linked protein products formed primarily through the reaction of plasma proteins with chlorinated oxidants produced by myeloperoxidase in neutrophils [10,11]. It is generally accepted that the majority of plasma AOPPs originate from oxidized albumin, fibrinogen, and collagen [12,13,14]. AOPPs were first recognized as novel biomarkers for oxidative stress in the plasma of patients with uremia [15]. However, increased AOPP levels in plasma were also observed in various other pathophysiological states, including liver diseases, hypertriglyceridemia, and hypercholesterolemia, type 1 and type 2 diabetes (T2D), obesity, and MS, as well as atherosclerosis and coronary artery disease [11,12,13,16,17,18]. AOPPs are very stable compounds that are resistant to proteolytic degradation and reduction by antioxidants [11,19]. Through their interactions with the receptor of advanced glycation end-products and CD36, AOPPs induce the activities of various signaling molecules, such as protein kinase C, NADPH oxidase, and nuclear factor kB, which promote the generation of ROS, inflammation, and endothelial dysfunction [17,20,21]. Additionally, through its high affinity binding to the high-density lipoprotein (HDL) receptor scavenger receptor class B type 1 (SR-BI), AOPP-albumin strongly interferes with the plasma clearance of HDL cholesterol ester via SR-BI in mice [22], suggesting a link between AOPPs and the metabolism of lipoproteins.

Previous studies have reported a negative association between AOPPs and HDL cholesterol (HDL-C) as well as a positive association between AOPPs and triglyceride levels in plasma in adults with and without MS as well as children with severe obesity [7,8,23,24]. However, to our knowledge, no study has examined the associations between AOPPs and the lipoprotein subclasses in healthy subjects and patients with MS. Therefore, in the present study, we determined the serum levels of AOPPs as well as the serum levels and lipid contents of lipoprotein subclasses and examined their association in healthy volunteers (HVs) and patients with MS.

## 2. Materials and Methods

### 2.1. Study Design, Participants, and Laboratory Procedures

The study design, laboratory procedures, as well as inclusion and exclusion criteria have been described in our previous reports [25,26]. This study was approved by the local ethics committees of the Sisters of Charity University Hospital Centre, Zagreb, Croatia (EP 13125/17-4); the University of Zagreb, School of Medicine, Croatia; and the Medical University of Graz, Austria (31-532 ex 18/19). A signed informed consent form was obtained from all participants, and the study was performed in accordance with the principles of the Good Clinical Practice Guidelines and the Declaration of Helsinki [27].

### 2.2. Lipoprotein Profiling Using Nuclear Magnetic Resonance (NMR) Spectroscopy

Serum lipoprotein subclasses were measured on a Bruker 600 MHz Avance Neo NMR spectrometer (Bruker, Rheinstetten, Germany) using the Bruker IVDr lipoprotein subclass analysis protocol, as described previously [28,29].

### 2.3. Calculation of the Lipid Content of Lipoprotein Particles

The lipid contents of apolipoprotein (apo)B-containing lipoprotein particles, including very-low-density lipoprotein (VLDL), intermediate-density lipoprotein (IDL), and low-density lipoprotein (LDL), were calculated as the ratios between the serum levels of the respective lipid (mg/dL) and the serum levels of apoB in the apoB-containing lipoprotein (mg/dL). The lipid contents of HDL were calculated as the ratios between the serum levels of the respective lipid (mg/dL) and the serum levels of apo A–I in HDL (mg/dL).

### 2.4. Quantification of AOPPs

Serum levels of AOPPs were measured as described in [30] with slight modifications. All assays were carried out in triplicate with eight samples analyzed per microtiter plate. Serum was diluted 1:10 in phosphate-buffered saline (PBS), and 300 μL of the diluted serum samples were added to each well of the 96-well microtiter plate (Corning, Costar, Merck KGaA, Darmstadt, Germany). Chloramine-T hydrate (Merck KGaA, Darmstadt, Germany) standard solutions (300 μL; 0–100 μM) were loaded on the same plate, and PBS was used as a sample blank. Fifteen microliters of 1.16 mol/L potassium iodide (Merck KGaA, Darmstadt, Germany) was added to all wells, followed by incubation at room temperature for 2 min. Finally, 30 μL glacial acetic acid (Merck KGaA, Darmstadt, Germany) was added to all wells. Afterwards, the plate was centrifuged at 2934× *g* (in contrast to 5800× *g* in [30]) for 5 min, and 230 μL precipitated-protein-free supernatant was transferred to the free wells of the same 96-well microtiter plate (in contrast to transferring onto new plate in [30]). The absorbance was determined at 340 nm using Epoch^TM^ (Bio-Tek Instruments GmbH, Bad Friedrichshall, Germany). The absorbance of the sample blank was subtracted from all wells before further analysis. The serum concentrations of AOPPs are expressed in μmol/L chloramine T equivalents.

### 2.5. Statistical Analyses

Depending on the data distribution, absolute and relative frequencies were used for the description of qualitative variables, whereas quantitative variables were summarized using mean and standard deviations (SDs) or medians and interquartile ranges (q1 and q3). To assess differences between HV and MS patients, as well as between HV and MS patients with low and high AOPPs (according to the within-group medians), either Fisher’s exact test, *t* test, or Mann–Whitney U test was used. Correlation analyses using Spearman’s correlation coefficient were performed separately for HVs and patients with MS. The impacts of confounders, including age, sex, body mass index (BMI), C-reactive protein (CRP), serum protein levels (protein), T2D, and statins, on the associations between AOPPs and the serum levels and lipid contents of the lipoprotein subclasses were examined by partial correlation analysis using the following four adjustment models: Model 1: age, sex, and BMI; Model 2: age, sex, BMI, and C-reactive protein (CRP); Model 3: age, sex, BMI, and protein; and Model 4: age, sex, T2D, and statin.

Furthermore, the discriminatory power of the lipoproteins to separate people with high and low AOPPs was examined in HVs and patients with MS separately using orthogonal partial least squares discriminant analyses (OPLS-DAs) [31]. These analyses, together with the associated data consistency checks and 7-fold cross-validation [32], were performed using MetaboAnalyst version 5.0 [33].

A *p*-value of <0.05 was considered significant for differences in the demographic and clinical characteristics as well as standard laboratory data. However, when assessing differences in the serum levels and lipid contents of the lipoprotein subclasses between groups as well as for correlation analyses, a Bonferroni correction was applied to correct for multiple testing, and thus, a *p*-value of <0.0003 (=0.05/151) was considered significant. R version 4.1.0 was used for the statistical analyses.

## 3. Results

### 3.1. Patient Characteristics, Laboratory Data, and Serum Levels of AOPPs in HVs and Patients with MS 

The demographic and clinical characteristics as well as the standard laboratory data of the study groups have been described previously [25,26] and are presented in Tables S1 and S2. The serum levels of AOPPs were significantly higher in the patients with MS compared to the HVs (HV: 34.6 (28.9, 42.6) μmol/L vs. MS: 41.6 (30.9, 57.3) μmol/L, *p* = 0.004). The differences in lipoprotein parameters between HVs and patients with MS have also been described previously [25,26] and are presented in Tables S3–S8.

### 3.2. Correlation Analyses between AOPPs and the Serum Levels and Lipid Contents of VLDL

**Figure 1:**
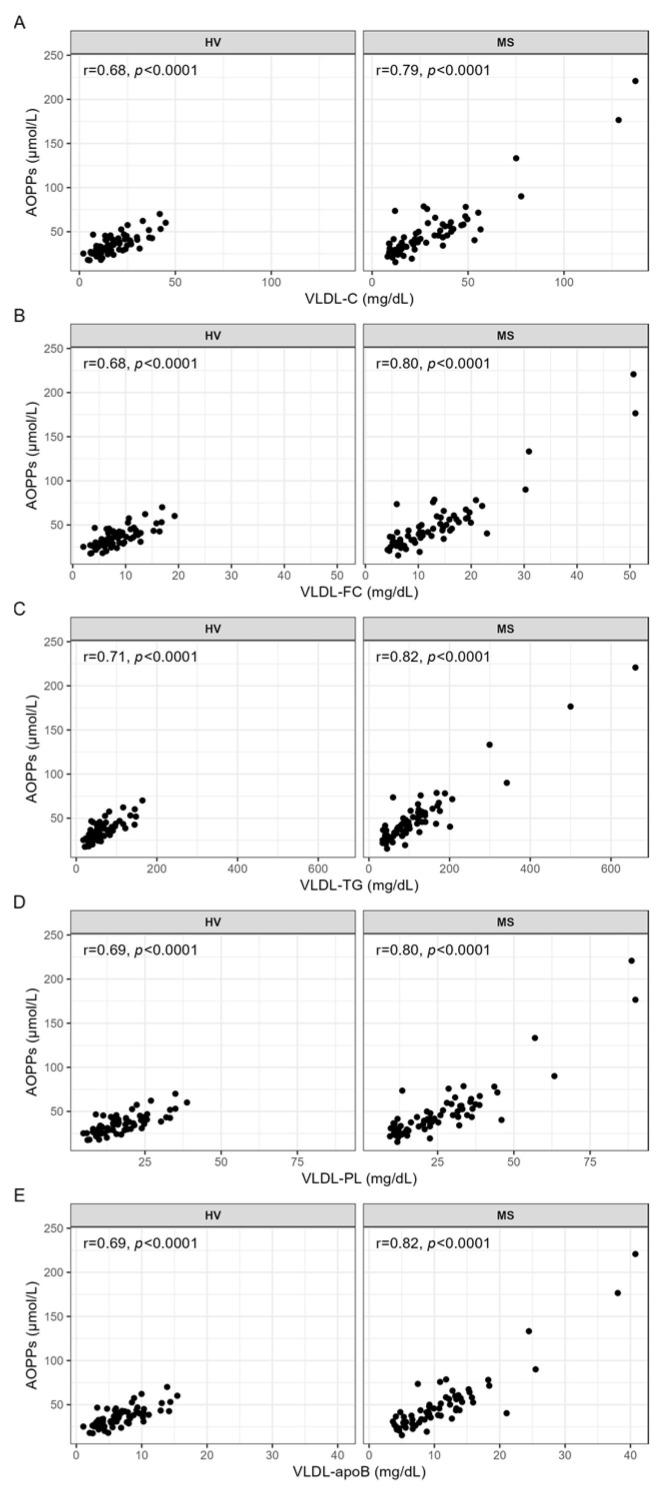
Correlation analyses between AOPPs and the serum levels of VLDL, carried out separately for HVs and patients with MS. Spearman correlation analyses were used to evaluate the associations between the serum levels of AOPPs and (**A**) VLDL-C, (**B**) VLDL-FC, (**C**) VLDL-TG, (**D**) VLDL-PL, and (**E**) VLDL-apoB. *p*-values < 0.0003 are considered statistically significant after a Bonferroni correction for multiple testing. AOPPs, advanced oxidation protein products; apoB, apolipoprotein B; C, cholesterol; FC, free cholesterol; HV, healthy volunteer; MS, patient with metabolic syndrome; PL, phospholipid; r, Spearman’s correlation coefficient; TG, triglyceride, VLDL, very-low-density lipoprotein.

In both the HVs and patients with MS, the serum levels of AOPPs were positively correlated with the serum levels of total cholesterol (VLDL-C), free cholesterol (VLDL-FC), triglycerides (VLDL-TG), phospholipids (VLDL-PL), and apoB (VLDL-apoB) in the total VLDL (Figure 1A–E) as well as in VLDL subclasses 1–4 (Table S9). These associations were stronger in the patients with MS than in the HVs (Figure 1 and Table S9).

**Figure 2:**
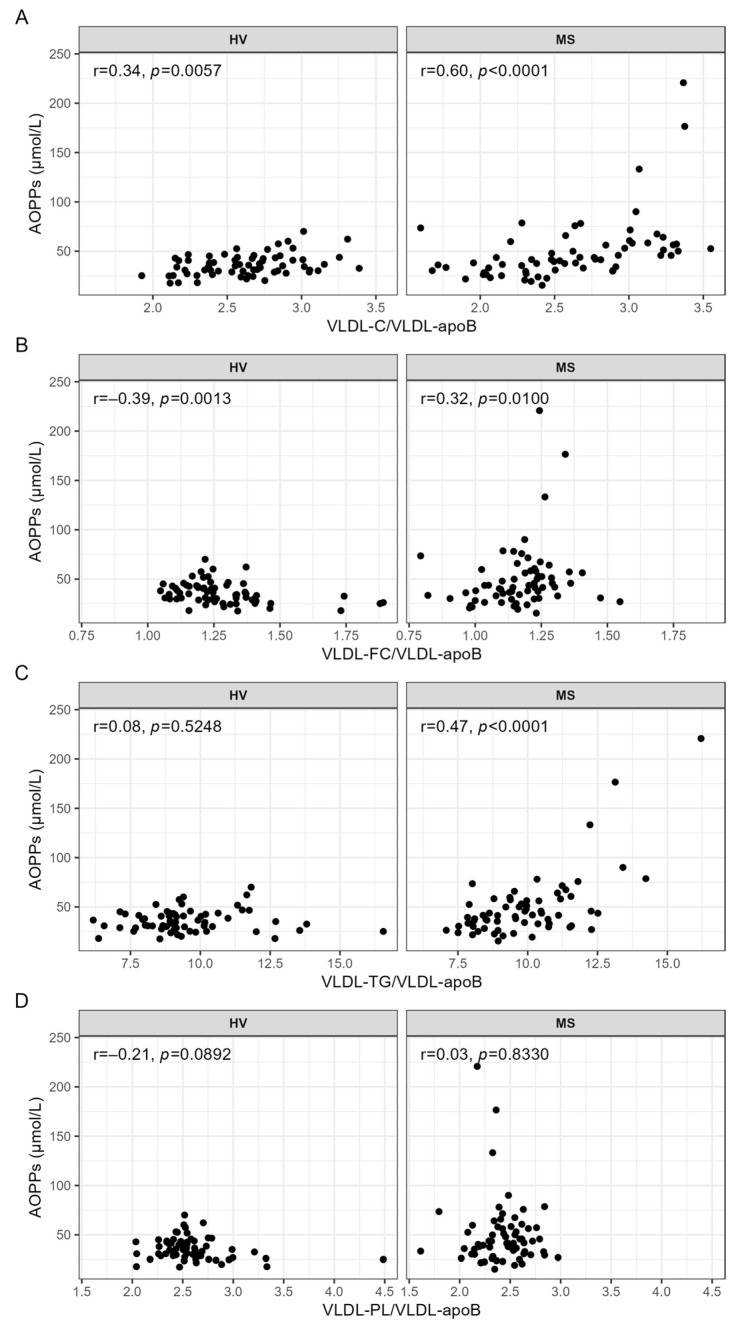
Correlation analyses between AOPPs and the lipid contents of VLDL, carried out separately for HVs and patients with MS. Spearman correlation analyses were used to evaluate the associations between the serum levels of AOPPs and (**A**) VLDL-C/VLDL-apoB, (**B**) VLDL-FC/VLDL-apoB, (**C**) VLDL-TG/VLDL-apoB, and (**D**) VLDL-PL/VLDL-apoB. *p*-values < 0.0003 are considered statistically significant after a Bonferroni correction for multiple testing. AOPPs, advanced oxidation protein products; apoB, apolipoprotein B; C, cholesterol; FC, free cholesterol; HV, healthy volunteer; MS, patient with metabolic syndrome; PL, phospholipid; r, Spearman’s correlation coefficient; TG, triglyceride, VLDL, very low-density lipoprotein.

While the serum levels of AOPPs were not correlated with either indicator of lipid contents of VLDL in the HVs (Figure 2A–D), the serum levels of AOPPs were significantly positively correlated with the cholesterol (VLDL-C/VLDL-apoB) (Figure 2A) and triglyceride (VLDL-TG/apoB) (Figure 2C) content of VLDL in the patients with MS.

As revealed by the partial correlation analyses, the associations between the serum levels of AOPPs and the VLDL parameters were not affected by age, sex, or BMI, nor by the addition of either CRP or protein, in the HVs or in the patients with MS (Tables S10 and S11). Since 41.5% of the patients with MS had T2D and 35.4% used statins, we also examined the impact of these confounders on the associations between the serum levels of AOPPs and the VLDL parameters in patients with MS. Adjusting for age, sex, T2D, and statin use only rendered an insignificant correlation between the serum levels of AOPPs and VLDL-TG/apoB (Table S11).

### 3.3. Correlation Analyses between AOPPs and the Serum Levels and Lipid Contents of IDL

**Figure 3:**
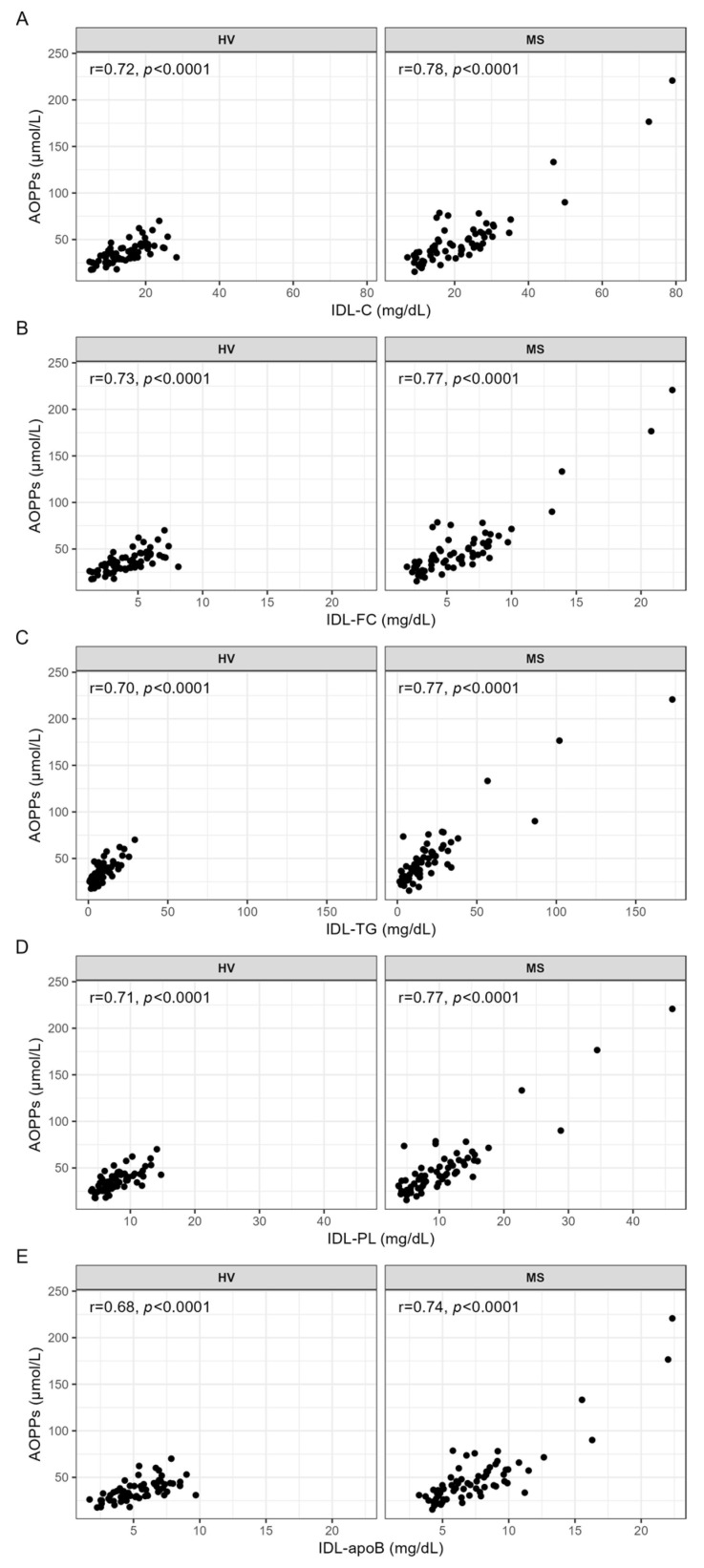
Correlation analyses between AOPPs and the serum levels of IDL, carried out separately for HVs and patients with MS. Spearman correlation analyses were used to evaluate the associations between the serum levels of AOPPs and (**A**) IDL-C, (**B**) IDL-FC, (**C**) IDL-TG, (**D**) IDL-PL, and (**E**) IDL-apoB. *p*-values < 0.0003 are considered statistically significant after a Bonferroni correction for multiple testing. AOPPs, advanced oxidation protein products; apoB, apolipoprotein B; C, cholesterol; FC, free cholesterol; HV, healthy volunteer; IDL, intermediate-density lipoprotein; MS, patient with metabolic syndrome; PL, phospholipid; r, Spearman’s correlation coefficient; TG, triglyceride.

The serum levels of AOPPs were strongly positively correlated with the serum levels of cholesterol (IDL-C), free cholesterol (IDL-FC), triglycerides (IDL-TG), phospholipids (IDL-PL), and apoB (IDL-apoB) in IDL (Figure 3A–E) in both the HVs and patients with MS.

**Figure 4:**
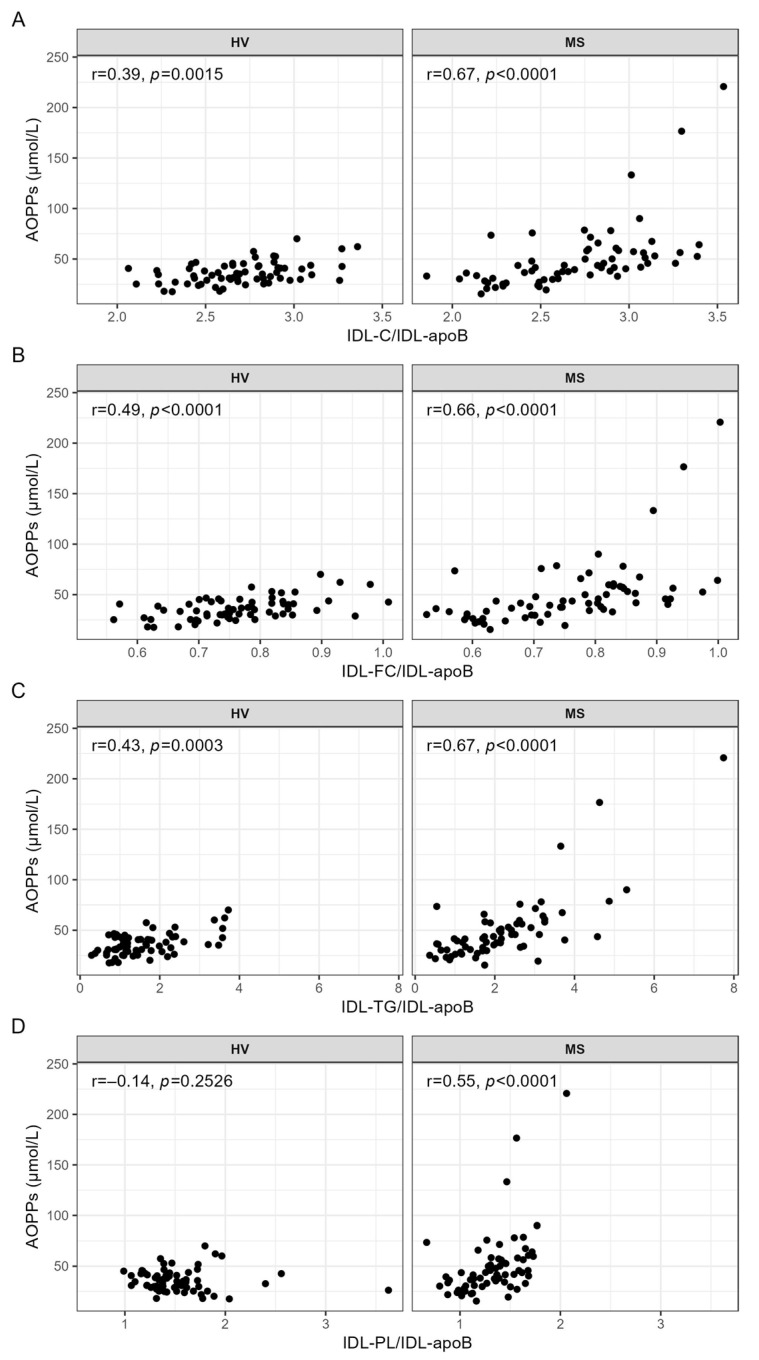
Correlation analyses between AOPPs and the lipid contents of IDL, carried out separately for the HVs and patients with MS. Spearman correlation analyses were used to evaluate the associations between the serum levels of AOPPs and (**A**) IDL-C/IDL-apoB, (**B**) IDL-FC/IDL-apoB, (**C**) IDL-TG/IDL-apoB, and (**D**) IDL-PL/IDL-apoB. *p*-values < 0.0003 are considered statistically significant after a Bonferroni correction for multiple testing. AOPPs, advanced oxidation protein products; apoB, apolipoprotein B; C, cholesterol; FC, free cholesterol; HV, healthy volunteer; IDL, intermediate-density lipoprotein; MS, patient with metabolic syndrome; PL, phospholipid; r, Spearman’s correlation coefficient; TG, triglyceride.

While the serum levels of AOPPs were strongly and significantly correlated with all ratios indicating the lipid contents of IDL in the patients with MS (Figure 4A–D), only IDL-FC/IDL-apoB (Figure 4B) was significantly (positively) correlated with AOPPs in the HVs.

All correlations remained significant after adjusting for the parameters from Model 1 to Model 4 mentioned above (Tables S12 and S13).

### 3.4. Correlation Analyses between AOPPs and the Serum Levels of LDL

As shown in Table 1, the serum levels of AOPPs were significantly positively correlated with the serum levels of cholesterol in small dense LDL subclasses 5 (LDL5-C) and 6 (LDL6-C) in the HVs, as well as small dense LDL subclass 6 (LDL6-C) in the patients with MS.

Additionally, the serum levels of AOPPs were significantly positively correlated with the serum levels of free cholesterol in LDL subclass 5 (LDL5-FC) in the HVs and subclass 6 (LDL6-FC) in the patients with MS. We found significant positive correlations between the serum levels of AOPPs and the serum levels of TG in the total LDL (LDL-TG) in both the HVs and patients with MS, as well as with LDL subclasses 4 (LDL4-TG) and 5 (LDL5-TG) in the HVs and LDL subclasses 1 (LDL1-TG), 5 (LDL5-TG), and 6 (LDL6-TG) in the patients with MS. Furthermore, the serum levels of AOPPs were significantly positively correlated with the serum levels of PL in small dense LDL subclass 5 (LDL5-PL) in the HVs and subclass 6 (LDL6-PL) in the patients with MS, as well as with the serum levels of apoB in subclasses 4–6 (LDL4-apoB, LDL5-apoB, LDL6-apoB) in the HVs and in subclass 6 (LDL6-apoB) in the patients with MS. However, no significant correlations were found between the serum levels of AOPPs and the serum levels of PL or apoB in the total LDL or in large buoyant subclasses 1–3 in the HVs nor in the patients with MS. While the association between the serum levels of AOPPs and LDL6-C turned insignificant after adjusting for age, sex, BMI, and CRP or protein in the HVs (Model 2 and Model 3), the associations between the serum levels of AOPPs and LDL5-FC, as well as LDL4-apoB, were rendered insignificant after adjusting for age, sex, and BMI (Model 1), as well as age, sex, BMI, and CRP or protein in the patients with MS (Model 2 and Model 3; Table S14). In contrast, adjusting for these confounders rendered an insignificant association between the serum levels of AOPPs and LDL1-TG in the HVs significant. In the patients with MS, all associations remained significant after adjustments (Table S15).

### 3.5. Correlation Analyses between AOPPs and the Lipid Content of LDL

The associations between the serum levels of AOPPs and the lipid contents of LDL are shown in Table 2. We found significant negative correlations between the serum levels of AOPPs and the cholesterol content of total LDL (LDL-C/LDL-apoB) in the HVs and patients with MS, as well as the cholesterol content of LDL subclass 2 (LDL2-C/LDL2-apoB) in the HVs and LDL subclasses 3 (LDL3-C/LDL3-apoB) and 4 (LDL4-C/LDL4-apoB) in the patients with MS. Furthermore, the serum levels of AOPPs were significantly negatively correlated with the FC content of the total LDL (LDL-FC/LDL-apoB) in the HVs and patients with MS, as well as with the FC content of LDL subclasses 3–6 in the HVs and LDL subclasses 5 (LDL5-FC/LDL5-apoB) and 6 (LDL6-FC/LDL6-apoB) in the patients with MS.

Significant negative correlations were also found between the serum levels of AOPPs and the PL content of total LDL (LDL-PL/LDL-apoB) in the HVs and patients with MS, as well as the PL content of LDL subclasses 2–6 in HVs and subclasses 1–6 in patients with MS. In contrast to the negative correlations between the serum levels of AOPPs and the cholesterol (LDL-C/LDL-apoB), free cholesterol (LDL-FC/LDL-apoB), and phospholipid (LDL-PL/LDL-apoB) contents of total LDL described above, we observed a significant positive correlation between AOPPs and the triglyceride content of large buoyant LDL subclass 1 (LDL1-TG/LDL1-apoB) in both HVs and patients with MS, but a significant positive correlation between AOPPs and the triglyceride content of small dense LDL subclass 5 (LDL5-TG/LDL5-apoB) was only observed in patients with MS. All significant associations between the serum levels of AOPPs and the indicators of the lipid contents of LDL remained significant after adjustment (Tables S16 and S17). However, an insignificant association between the serum levels of AOPPs and LDL2-TG/LDL2-apoB in the HVs turned significant after adjusting for Model 1 and Model 3 (Table S16). Additionally, the initially insignificant associations between the serum levels of AOPPs and LDL5-C/LDL5-apoB as well as LDL2-TG/LDL2-apoB in the patients with MS were rendered significant after adjusting for any of our four adjustment models (Table S17).

### 3.6. Correlation Analyses between AOPPs and the Serum Levels of HDL

As shown in Table 3, the serum levels of AOPPs were significantly negatively correlated with the serum levels of cholesterol in the total HDL (HDL-C) in both the HVs and patients with MS.

The serum levels of AOPPs were significantly negatively correlated with the serum levels of cholesterol in large buoyant HDL subclasses 1 (HDL1-C) and 2 (HDL2-C) in the HVs, but with small dense HDL subclasses 3 (HDL3-C) and 4 (HDL4-C) in the patients with MS. The serum levels of AOPPs were also significantly negatively correlated with the serum levels of free cholesterol in the total HDL (HDL-FC) in both the HVs and patients with MS, but were only significantly negatively correlated with the serum levels of free cholesterol in HDL subclasses 1 (HDL1-FC) and 2 (HDL2-FC) in the HVs. While significant positive correlations between AOPPs and the serum levels of triglycerides in small dense HDL subclasses 3 (HDL3-TG) and 4 (HDL4-TG) were observed in both the HVs and patients with MS, the serum levels of AOPPs were only significantly positively correlated with the serum levels of triglycerides in the total HDL (HDL-TG) and in HDL subclass 2 (HDL2-TG) in the patients with MS.

Furthermore, the serum levels of AOPPs were significantly negatively correlated with the serum levels of phospholipids in the total HDL (HDL-PL) in both the HVs and patients with MS, as well as with the serum levels of phospholipids in large buoyant HDL subclasses 1 (HDL1-PL) and 2 (HDL1-PL) in HVs, and with the serum levels of phospholipids in small dense subclass 4 (HDL4-PL) in patients with MS. Moreover, the serum levels of AOPPs were significantly negatively correlated with the serum levels of apoA-I in large buoyant HDL subclasses 1 (HDL1-apoA-I) and 2 (HDL2-apoA-I) in the HVs, and with the serum levels of apoA-I in the total HDL (HDL-apoA-I) as well as in small dense HDL subclass 4 (HDL4-apoA-I) in the patients with MS. The only significant correlation between the serum levels of AOPPs and the serum levels of apoA-II was found in the HVs, in which the serum levels of AOPPs were significantly negatively correlated with the serum levels of apoA-II in large buoyant HDL subclass 1 (HDL1-apoA-II).

Adjusting for age, sex, BMI, and CRP or protein rendered the correlations between AOPPs and HDL2-PL as well as between AOPPs and HDL1-apoA-II in the HVs insignificant (Table S18). The correlation between the serum levels of AOPPs and HDL2-FC in the HVs was also rendered insignificant upon adjustment (Table S18). In the patients with MS, the correlation between the serum levels of AOPPs and HDL3-C turned insignificant after adjusting for Model 4 only, whereas that between the serum levels of AOPPs and HDL-FC was generally rendered insignificant upon adjustment, including those between the serum levels of AOPPs and HDL-apoA-I and HDL4-apoA-I after adjustments for Models 1, 3, and 4 (Table S19).

### 3.7. Correlation Analyses between AOPPs and the Lipid Contents of HDL Particles

The correlations between the serum levels of AOPPs and the lipid contents of HDL are shown in Table 4.

We found significant negative correlations between the serum levels of AOPPs and the cholesterol (HDL-C/HDL-apoA-I) and phospholipid (HDL-PL/HDL-apoA-I) contents of total HDL in both the HVs and patients with MS. Furthermore, significant negative correlations between the serum levels of AOPPs and the cholesterol content of small dense HDL subclasses 3 (HDL3-C/HDL3-apoA-I) and 4 (HDL4-C/HDL4-apoA-I), as well as the phospholipid content of small dense HDL subclass 4 (HDL4-PL/HDL4-apoA-I), were observed in the patients with MS, while the serum levels of AOPPs were significantly positively correlated with the triglyceride content of HDL subclasses 1–4 in both the HVs and patients with MS (the associations were stronger in the patients with MS than in the HVs); a significant positive correlation between the serum levels of AOPPs and the triglyceride content of total HDL (HDL-TG/HDL-apoA-I) was only observed in the patients with MS, and a significant negative correlation between the serum levels of AOPPs and the free cholesterol content of total HDL (HDL-FC/HDL-apoA-I) was only found in the HVs.

In the HVs, adjusting for age, sex, BMI, and protein rendered the correlation between the serum levels of AOPPs and HDL-PL/HDL-apoA-I insignificant, whereas the initially insignificant correlation between the serum levels of AOPPs and HDL4-C/HDL4-apoA-I became significant after the same adjustment, as did the correlation between the serum levels of AOPPs and HDL-TG/HDL-apoA-I after any adjustment (Table S20).

### 3.8. Relative Contributions of Lipoprotein Parameters to Low and High AOPPs in HVs and Patients with MS 

**Figure 5:**
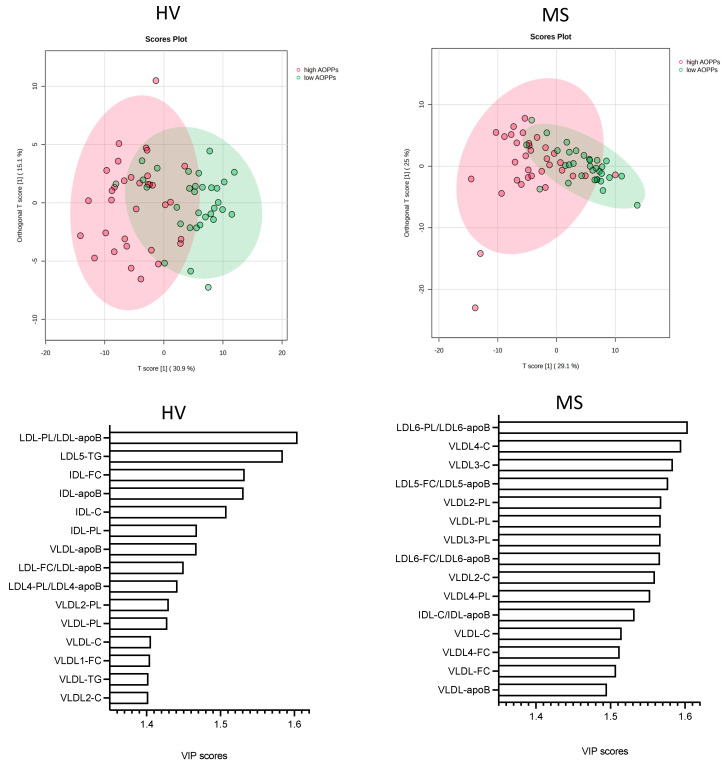
Associations of lipoprotein parameters with low and high AOPPs, determined separately for HVs and patients with MS. Differences in lipoprotein parameters between (**A**) HVs with AOPPs < 34.6 μmol/L (low AOPPs) and those with AOPPs ≥ 34.6 μmol/L (high AOPPs) as well as between (**B**) patients with MS with AOPPs < 41.6 μmol/L (low AOPPs) and those with AOPPs ≥ 41.6 μmol/L (high AOPPs) were examined with OPLS-DA. Top 15 VIP scores reflecting contribution of parameters to model in (**C**) HVs and (**D**) patients with MS. AOPPs, advanced oxidation protein products; apoB, apolipoprotein B; C, cholesterol; FC, free cholesterol; HV, healthy volunteer; IDL, intermediate-density lipoprotein; LDL, low-density lipoprotein; MS, patient with metabolic syndrome; OPLS-DA, orthogonal partial least squares discriminant analysis; PL, phospholipid; TG, triglyceride, VIP, variable of importance projection; VLDL, very-low-density lipoprotein.

After investigating the associations between the serum levels AOPPs and the lipoprotein parameters using correlation analyses, we performed multivariate analyses using OPLS-DA to examine the relative contributions of the lipoprotein parameters to the separation of samples into low and high AOPPs in the HVs and patients with MS. Low AOPPs samples in the HVs and patients with MS were those below the respective median (34.6 μmol/L for HV; 41.6 μmol/L for MS), whereas high AOPP samples were those ≥34.6 and ≥41.6 μmol/L, respectively. The differences in the serum levels and lipid contents of lipoproteins between participants with low and high AOPPs, calculated separately for HVs and patients with MS, are described in Results S1 and shown in Tables S22–S33. The OPLS-DA analysis revealed a partial clustering, but not a full separation of the lipoprotein parameters into high and low AOPPs in both the HVs and patients with MS (Figure 5A,B). The correlation coefficients in the HVs and patients with MS were R^2^Y = 0.542 (*p* < 0.001) and R^2^Y = 0.492 (*p* < 0.001), respectively, and the cross-validation scores were Q^2^ = 0.382 (*p* < 0.001) and Q^2^ = 0.362 (*p* < 0.001), respectively (Figure S1).

The relative contributions of the lipoprotein parameters to the separation of the samples into low and high serum levels of AOPPs, reflecting the strength of the associations between the lipoprotein parameters and the serum levels of AOPPs, were determined by variable of importance (VIP) scores (the VIP scores of all parameters in HVs and patients with MS are presented in Table S34). In both the HVs and patients with MS, the top fifteen lipoprotein parameters with the highest VIP scores comprised various IDL, LDL, and VLDL parameters, but none of the HDL parameters (Figure 5C,D). The top six parameters in HVs comprised two LDL parameters, namely the phospholipid content of total LDL (LDL-PL/LDL-apoB) and the serum levels of triglycerides in LDL subclass 5 (LDL5-TG), as well as the four IDL parameters including IDL-FC, IDL-apoB, IDL-C, and IDL-PL (Figure 5C). In the patients with MS, the top six parameters comprised the phospholipid content of LDL subclass 6 (LDL6-PL/LDL-apoB), the free cholesterol content of LDL subclass 5 (LDL5-FC/LDL5-apoB) (Figure 5D), as well as four VLDL parameters, namely the serum levels of total cholesterol in subclasses 3 and 4 (VLDL3-C, VLDL4-C) and the serum levels of phospholipids in total VLDL (VLDL-PL) and VLDL subclass 2 (VLDL2-PL). In the HVs, the residual parameters within the top fifteen comprised seven indicators of the serum levels of lipids and apoB in VLDL (VLDL-apoB, VLDL2-PL, VLDL-PL, VLDL-C, VLDL1-FC, VLDL-TG, and VLDL2-C), as well as two LDL parameters, namely the ratio indicating the free cholesterol content of total LDL (LDL-FC/LDL-apoB) and the ratio indicating the phospholipid content of LDL subclass 4 (LDL4-PL/LDL4-apoB). In the patients with MS, the residual parameters also comprised seven indicators of the serum levels of lipids and apoB in VLDL (VLDL3-PL, VLDL2-C, VLDL4-PL, VLDL-C, VLDL4-FC, VLDL-FC, and VLDL-apoB), as well as the ratio indicating the free cholesterol content of LDL subclass 6 (LDL6-FC/LDL6-apoB) and the ratio indicating the cholesterol content of IDL (IDL-C/IDL-apoB) (Figure 5C,D).

## 4. Discussion

Here, we describe, for the first time, the associations between the serum levels of AOPPs and the lipoprotein subclasses in HVs and patients with MS. Previous studies have reported AOPPs to have a negative association with HDL-cholesterol (HDL-C) and a positive association with triglyceride plasma levels in subjects without and with MS [7,8]. Considering the fact that HDL-C only reflects the serum levels of cholesterol carried by HDL, but not of the other HDL lipids and apolipoproteins, and that fasting serum triglyceride levels represent the sum of triglycerides carried by VLDL, IDL, LDL, and HDL without reflecting the relative contribution of the single lipoprotein classes (and subclasses), it is obvious that the available knowledge regarding associations between the serum levels of AOPPs and lipoproteins is limited. Additionally, the associations between the serum levels of AOPPs and the lipid contents of serum lipoproteins remain fully unexplored. Therefore, in the present study, we employed NMR spectroscopy and performed comprehensive serum lipoprotein profiling to determine the serum levels of lipids and apolipoproteins carried by various lipoproteins, as well as their subclasses. We also calculated the lipid contents of the lipoprotein particles as ratios of the lipids and apolipoproteins carried by the respective lipoproteins and examined the associations between AOPPs and lipoprotein parameters.

Here, we show that in both HVs and patients with MS, the IDL and VLDL parameters exhibit the most profound associations with AOPPs, followed by the LDL and HDL parameters. These findings highlight a high susceptibility to the oxidation of IDL and VLDL, as well as the importance of oxidized IDL and VLDL as carriers and sources of serum AOPPs. This may, at least in part, explain the established pro-atherogenic effects of triglyceride-rich lipoproteins and their remnants in cardiometabolic diseases [34]. The observed differences in the relative contribution of lipoprotein parameters as determinants of serum AOPPs between HVs and patients with MS most likely reflect the perturbations in the lipoprotein metabolism (and, consequently, the altered serum levels and lipid contents of circulating lipoproteins) and the increased oxidative stress in the context of the underlying MS pathophysiology. Along these lines, the lipid contents of VLDL and IDL were more profoundly associated with AOPPs in the patients with MS than in the HVs, suggesting that the lipid load of these lipoproteins (as well as of some LDL subclasses) is a more important modulator of the lipoprotein oxidation susceptibility under the pathophysiological conditions of MS than under healthy physiological conditions.

In agreement with a higher susceptibility to the oxidative modification of small dense compared to large buoyant LDL described in previous studies [35,36,37], we found positive associations between AOPPs and the serum levels of small dense LDL subclasses 4–6, but not large buoyant subclasses 1–3 (with the exception of LDL1-TG in MS). These associations were more profound and more resistant to the impact of confounders in patients with MS than in the HVs, conceivably reflecting a perturbed metabolism and an increased oxidation susceptibility of LDL in the setting of the underlying MS pathophysiology. This fits with the established proatherogenic activities of small dense LDL particles, which, upon accumulation in the vascular subendothelial space, trigger an inflammatory response and promote foam cell formation, the initial and key events in atherogenesis [38]. Differences in the oxidative susceptibility of the LDL subclasses have been attributed to the structural and compositional features of LDL particles. It has been shown that the decreased free cholesterol content, decreased antioxidants such as ubiquinol-10, as well as enrichment with polyunsaturated free fatty acids underlie the increased oxidative susceptibility of small dense relative to large buoyant LDL [39,40,41]. In accordance with this, the FC content of small dense LDL was lower than that of large buoyant LDL in both the HVs and patients with MS (Table S6), and, importantly, the free cholesterol contents of LDL subclasses 3–6 in the HVs and subclasses 5 and 6 in patients with MS were negatively associated with AOPPs (Table 2). We also found negative associations between the phospholipid content of the LDL subclasses and AOPPs, highlighting the importance of phospholipids as enhancers of the antioxidative capacity of LDL. As the principal constituents of the LDL surface, free cholesterol and phospholipids determine the structural and physical properties of the surface and, in turn, they affect the conformation and, conceivably, the oxidative susceptibility of apoB [40,41]. Of note, the positive associations of AOPPs with the triglyceride content of large buoyant LDL subclass 1 in the HVs and patients with MS as well as with small dense subclass 5 in patients with MS (Table 2) indicate that not only the lipids which constitute the LDL surface, but also the triglycerides in the core, modulate the oxidative susceptibility of LDL.

In agreement with the established antioxidative capacity of HDL-associated proteins, enzymes, and lipids [42,43], we found negative associations between the HDL subclasses and AOPPs, with striking differences between HVs and patients with MS regarding the relative contributions of the HDL subclasses to these associations, while in the HVs, rather large buoyant HDL subclasses were negatively associated with AOPPs, and small dense subclasses exhibited negative associations with AOPPs in patients with MS. These findings suggest that small dense HDL particles exert antioxidative protection only in the context of the MS pathophysiology but not in a healthy state. However, previous studies have found a stronger antioxidative capacity of small dense compared to large buoyant HDL particles isolated from healthy subjects as well as an impaired antioxidative capacity of HDL isolated from patients with MS [44,45,46,47]. This discrepancy most likely reflects the differences in the nature and design of our study and the other studies. More specifically, the associations between AOPPs and the HDL subclasses examined in our study reflect the relationships between the bioavailability of AOPPs and HDL in vivo; however, they do not provide direct evidence of the antioxidative activity of HDL. In contrast, the other experimental studies examined the antioxidative activity of isolated HDL, but under specific experimental conditions in vitro [44,45,46,47], and therefore, the measured HDL activities may not necessarily reflect the activities of HDL in the complex (patho)physiological environment in vivo. In both the HVs and patients with MS, several associations between AOPPs and the HDL parameters turned insignificant after adjusting for age, sex, BMI, and CRP, thus highlighting the role of these (patho)physiological confounders in the modulation of the composition and function of HDL [48,49,50,51].

In the present study, we found negative associations of AOPPs with the cholesterol and phospholipid contents as well as positive associations with the triglyceride content of HDL. These findings are in accordance with the results of experimental studies showing a negative effect of triglyceride and a positive effect of cholesterol and lysophospholipid enrichment of HDL on the antioxidative activity of HDL [46,52,53,54].

HDL comprises a subpopulation of particles that contain only apoA-I or both apoA-I and apoA-II, as well as a minor subpopulation that contains only apoA-II [55,56]. Accordingly, the negative correlations between AOPPs and HDL1-apoA-I as well as HDL1-apoA-II in the HVs observed in the present study (Table 3 most likely reflect the associations between AOPPs and the HDL particles that contain both apoA-I and apoA-II. In contrast, the observed negative correlations between AOPPs and HDL2-apoA-I in the HVs and HDL4-apoA-I in patients with MS (but no correlations between AOPPs and the corresponding subclasses of HDL-apoA-II) suggest associations of AOPPs with the HDL particles that contain only apoA-I.

Strengths and limitations: The major strength of our study is that it presents a comprehensive analysis of the serum levels and lipid contents of circulating lipoproteins, which allowed us to establish the relationships between these parameters and AOPPs. However, we need to acknowledge several limitations: Due to the design of the present study, we could not examine the causality for the relationship between AOPPs and the lipoprotein subclasses. Accordingly, the mechanistic relationship between AOPPs and the lipoprotein subclasses could not be addressed. Since we did not quantify AOPPs on the isolated lipoproteins, one can argue that the observed positive associations between AOPPs and the lipoproteins are not due to the AOPPs associated with the lipoproteins, but rather the AOPPs generated by the oxidation of other serum proteins whose levels are positively correlated with the measured lipoproteins. However, adjusting for serum proteins did not significantly affect the majority of the observed associations. Additionally, in a previous study, the authors isolated lipoproteins from patients with renal diseases and found the highest levels of AOPPs in VLDL, followed by LDL and HDL [57]. These findings substantiate our results, which show the most pronounced positive associations between AOPPs and IDL and VLDL, followed by LDL, as well as negative associations with HDL. These facts strongly support the notion that, in the present study, AOPPs that are physically associated with the measured lipoproteins primarily underlie the observed associations between lipoproteins and AOPPs. However, considering that through its antioxidative activity, HDL protects not only itself but also other serum components from oxidation, it is likely that, in the case of HDL, the observed associations reflect the associations between HDL and the AOPPs associated physically with HDL, but also the AOPPs that arose upon the oxidation of other serum proteins. Since AOPPs reflect only the oxidative modification of serum proteins, future studies will need to examine the relationship between serum lipoproteins and other biomarkers of oxidative stress. Furthermore, the impact of other pathophysiological constellations, such as kidney, liver, or heart failure, on the associations between serum lipoproteins and biomarkers of oxidative stress will be addressed in the future.

## 5. Conclusions

Based on our results, we conclude that IDL, VLDL, and LDL facilitate whereas HDL diminishes the bioavailability of serum AOPPs. The presence of MS and the lipid contents of the subclasses affect the relationship between AOPPs and lipoproteins. Accordingly, lowering apoB-containing lipoproteins and concomitantly boosting the HDL serum levels, as well as modulating the lipoprotein lipid composition through life-style-driven or pharmacological treatments, may help reduce oxidative stress and its detrimental effects on cells, tissues, and organs, particularly the cardiovascular systems in HVs as well as patients with MS.

## Figures and Tables

**Table 1:** Correlation analyses of AOPPs with the serum levels of the LDL subclasses, carried out separately for the HVs and patients with MS.

	AOPPs (μmol/L)			
	HV (N = 65)		MS (N = 65)	
Variable (mg/dL)	r	*p*	r	*p*
LDL-C	0.26	0.0395	0.04	0.7305
LDL1-C	−0.03	0.8415	0.00	0.9841
LDL2-C	−0.30	0.0152	−0.30	0.0156
LDL3-C	−0.08	0.5151	−0.29	0.0181
LDL4-C	0.39	0.0011	−0.15	0.2323
LDL5-C	0.55	**<0.0001**	0.23	0.0658
LDL6-C	0.46	**0.0001**	0.72	**<0.0001**
LDL-FC	0.14	0.2657	−0.06	0.6482
LDL1-FC	0.00	0.9920	0.07	0.5599
LDL2-FC	−0.33	0.0074	−0.23	0.0632
LDL3-FC	−0.22	0.0835	−0.27	0.0288
LDL4-FC	0.27	0.0311	−0.17	0.1678
LDL5-FC	0.48	**0.0001**	0.16	0.1947
LDL6-FC	0.36	0.0032	0.70	**<0.0001**
LDL-TG	0.53	**<0.0001**	0.65	**<0.0001**
LDL1-TG	0.41	0.0008	0.55	**<0.0001**
LDL2-TG	0.12	0.3380	0.22	0.0732
LDL3-TG	0.00	0.9866	−0.07	0.5641
LDL4-TG	0.49	**<0.0001**	0.26	0.0335
LDL5-TG	0.58	**<0.0001**	0.63	**<0.0001**
LDL6-TG	0.33	0.0077	0.71	**<0.0001**
LDL-PL	0.23	0.0604	−0.02	0.8738
LDL1-PL	−0.02	0.8717	−0.02	0.8937
LDL2-PL	−0.30	0.0136	−0.34	0.0053
LDL3-PL	−0.10	0.4451	−0.31	0.0117
LDL4-PL	0.40	0.0008	−0.14	0.2602
LDL5-PL	0.54	**<0.0001**	0.20	0.1044
LDL6-PL	0.39	0.0012	0.70	**<0.0001**
LDL-apoB	0.41	0.0007	0.33	0.0084
LDL1-apoB	0.00	0.9700	0.06	0.6062
LDL2-apoB	−0.23	0.0660	−0.21	0.0912
LDL3-apoB	−0.02	0.8483	−0.25	0.0480
LDL4-apoB	0.47	**0.0001**	−0.05	0.6826
LDL5-apoB	0.58	**<0.0001**	0.34	0.0056
LDL6-apoB	0.52	**<0.0001**	0.76	**<0.0001**

The associations of the serum levels of AOPPs with the serum levels of lipids and apoB in the total LDL and the LDL subclasses were evaluated with Spearman correlation analyses. *p*-values < 0.0003 are considered statistically significant after a Bonferroni correction for multiple testing and are depicted in bold. AOPPs; advanced oxidation protein products; ApoB, apolipoprotein B; C, cholesterol; FC, free cholesterol; HV, healthy volunteer; LDL, low-density lipoprotein; MS, patient with metabolic syndrome; PL, phospholipid; r, Spearman’s correlation coefficient; TG, triglyceride.

**Table 2:** Correlation analyses of AOPPs with the lipid content of LDL particles, carried out separately for the HVs and patients with MS.

	AOPPs (μmol/L)			
	HV (N = 65)		MS (N = 65)	
	r	*p*	r	*p*
LDL-C/LDL-apoB	−0.49	**<0.0001**	−0.65	**<0.0001**
LDL1-C/LDL1-apoB	−0.14	0.2666	−0.19	0.1236
LDL2-C/LDL2-apoB	−0.51	**<0.0001**	−0.39	0.0016
LDL3-C/LDL3-apoB	−0.39	0.0015	−0.47	**0.0001**
LDL4-C/LDL4-apoB	−0.37	0.0025	−0.51	**<0.0001**
LDL5-C/LDL5-apoB	−0.24	0.0535	−0.44	0.0003
LDL6-C/LDL6-apoB	−0.27	0.0266	−0.40	0.0011
LDL-FC/LDL-apoB	−0.63	**<0.0001**	−0.71	**<0.0001**
LDL1-FC/LDL1-apoB	−0.12	0.3373	−0.15	0.2379
LDL2-FC/LDL2-apoB	−0.33	0.0081	−0.06	0.6157
LDL3-FC/LDL3-apoB	−0.48	**<0.0001**	−0.12	0.3210
LDL4-FC/LDL4-apoB	−0.60	**<0.0001**	−0.31	0.0146
LDL5-FC/LDL5-apoB	−0.68	**<0.0001**	−0.76	**<0.0001**
LDL6-FC/LDL6-apoB	−0.54	**<0.0001**	−0.75	**<0.0001**
LDL-TG/LDL-apoB	0.28	0.0227	0.39	0.0016
LDL1-TG/LDL1-apoB	0.53	**<0.0001**	0.51	**<0.0001**
LDL2-TG/LDL2-apoB	0.42	0.0005	0.43	0.0005
LDL3-TG/LDL3-apoB	−0.06	0.6281	0.30	0.0147
LDL4-TG/LDL4-apoB	0.21	0.0924	0.36	0.0038
LDL5-TG/LDL5-apoB	0.17	0.1756	0.48	**0.0001**
LDL6-TG/LDL6-apoB	−0.30	0.0164	−0.22	0.0772
LDL-PL/LDL-apoB	−0.66	**<0.0001**	−0.81	**<0.0001**
LDL1-PL/LDL1-apoB	−0.33	0.0075	−0.51	**<0.0001**
LDL2-PL/LDL2-apoB	−0.58	**<0.0001**	−0.63	**<0.0001**
LDL3-PL/LDL3-apoB	−0.57	**<0.0001**	−0.69	**<0.0001**
LDL4-PL/LDL4-apoB	−0.60	**<0.0001**	−0.68	**<0.0001**
LDL5-PL/LDL5-apoB	−0.65	**<0.0001**	−0.81	**<0.0001**
LDL6-PL/LDL6-apoB	−0.69	**<0.0001**	−0.79	**<0.0001**

Spearman correlation analyses were used to evaluate the associations between AOPPs and the lipid contents of LDL. *p*-values < 0.0003 are considered statistically significant after a Bonferroni correction for multiple testing and are depicted in bold. AOPPs, advanced oxidation protein products; ApoB, apolipoprotein B; C, cholesterol; FC, free cholesterol; HV, healthy volunteer; LDL, low-density lipoprotein; MS, patient with metabolic syndrome; PL, phospholipid; r, Spearman’s correlation coefficient; TG, triglyceride.

**Table 3:** Correlation analyses of AOPPs with the serum levels of HDL, performed separately for the HVs and patients with MS.

	AOPPs (μmol/L)			
	HV (N = 65)		MS (N = 65)	
Variable (mg/dL)	r	*p*	r	*p*
HDL-C	−0.55	**<0.0001**	−0.64	**<0.0001**
HDL1-C	−0.53	**<0.0001**	−0.05	0.6910
HDL2-C	−0.53	**<0.0001**	−0.29	0.0177
HDL3-C	−0.38	0.0017	−0.45	**0.0002**
HDL4-C	0.01	0.9070	−0.55	**<0.0001**
HDL-FC	−0.57	**<0.0001**	−0.45	**0.0002**
HDL1-FC	−0.56	**<0.0001**	−0.20	0.1132
HDL2-FC	−0.48	**0.0001**	−0.31	0.0108
HDL3-FC	−0.16	0.2175	−0.38	0.0015
HDL4-FC	0.09	0.4816	−0.40	0.0009
HDL-TG	0.27	0.0307	0.48	**0.0001**
HDL1-TG	−0.11	0.3783	0.43	0.0004
HDL2-TG	0.22	0.0777	0.45	**0.0002**
HDL3-TG	0.46	**0.0001**	0.47	**0.0001**
HDL4-TG	0.61	**<0.0001**	0.54	**<0.0001**
HDL-PL	−0.53	**<0.0001**	−0.54	**<0.0001**
HDL1-PL	−0.54	**<0.0001**	−0.16	0.2028
HDL2-PL	−0.47	**0.0001**	−0.21	0.0873
HDL3-PL	−0.27	0.0284	−0.41	0.0006
HDL4-PL	0.04	0.7253	−0.59	**<0.0001**
HDL-apoA-I	−0.44	0.0003	−0.45	**0.0002**
HDL1-apoA-I	−0.53	**<0.0001**	−0.11	0.3726
HDL2-apoA-I	−0.50	**<0.0001**	−0.28	0.0225
HDL3-apoA-I	−0.27	0.0292	−0.22	0.0779
HDL4-apoA-I	0.16	0.1998	−0.45	**0.0002**
HDL-apoA-II	0.05	0.7215	−0.10	0.4130
HDL1-apoA-II	−0.46	**0.0001**	0.03	0.8264
HDL2-apoA-II	−0.20	0.1130	0.13	0.3209
HDL3-apoA-II	0.20	0.1068	0.10	0.4126
HDL4-apoA-II	0.26	0.0391	−0.32	0.0088

Spearman correlation analyses were used to evaluate the associations between AOPPs and HDL subclasses. *p*-values < 0.0003 are considered statistically significant after a Bonferroni correction for multiple testing and are depicted in bold. AOPPs, advanced oxidation protein products; ApoA-I, apolipoprotein A-I; apoA-II, apolipoprotein A-II; C, cholesterol; FC, free cholesterol; HV, healthy volunteer; HDL, high-density lipoprotein; MS, patient with metabolic syndrome; PL, phospholipid; r, Spearman’s correlation coefficient; TG, triglyceride.

**Table 4:** Correlation analyses of AOPPs with the lipid contents of HDL particles, performed separately for HVs and patients with MS.

	AOPPs (μmol/L)			
	HV (N = 65)		MS (N = 65)	
Variable	r	*p*	r	*p*
HDL-C/HDL-apoA-I	−0.60	**<0.0001**	−0.57	**<0.0001**
HDL1-C/HDL1-apoA-I	0.34	0.0056	0.17	0.1702
HDL2-C/HDL2-apoA-I	−0.28	0.0222	−0.02	0.8962
HDL3-C/HDL3-apoA-I	−0.40	0.0009	−0.52	**<0.0001**
HDL4-C/HDL4-apoA-I	−0.32	0.0091	−0.63	**<0.0001**
HDL-FC/HDL-apoA-I	−0.59	**<0.0001**	−0.27	0.0279
HDL1-FC/HDL1-apoA-I	0.36	0.0035	−0.09	0.4651
HDL2-FC/HDL2-apoA-I	0.06	0.6221	−0.03	0.8004
HDL3-FC/HDL3-apoA-I	0.07	0.5528	−0.19	0.1278
HDL4-FC/HDL4-apoA-I	0.00	0.9694	−0.29	0.0206
HDL-TG/HDL-apoA-I	0.39	0.0012	0.66	**<0.0001**
HDL1-TG/HDL1-apoA-I	0.55	**<0.0001**	0.71	**<0.0001**
HDL2-TG/HDL2-apoA-I	0.45	**0.0001**	0.70	**<0.0001**
HDL3-TG/HDL3-apoA-I	0.54	**<0.0001**	0.73	**<0.0001**
HDL4-TG/HDL4-apoA-I	0.55	**<0.0001**	0.69	**<0.0001**
HDL-PL/HDL-apoA-I	−0.52	**<0.0001**	−0.45	**0.0002**
HDL1-PL/HDL1-apoA-I	0.34	0.0056	−0.13	0.3132
HDL2-PL/HDL2-apoA-I	−0.25	0.0443	0.05	0.6656
HDL3-PL/HDL3-apoA-I	−0.14	0.2657	−0.31	0.0125
HDL4-PL/HDL4-apoA-I	−0.25	0.0435	−0.66	**<0.0001**

The associations between AOPPs and the lipid contents of HDL particles were evaluated with Spearman correlation analyses. *p*-values < 0.0003 are considered statistically significant after a Bonferroni testing for multiple comparison and are depicted in bold. AOPPs, advanced oxidation protein products; ApoA-I, apolipoprotein A-I; C, cholesterol; FC, free cholesterol; HV, healthy volunteer; HDL, high-density lipoprotein; MS, patient with metabolic syndrome; PL, phospholipid; r, Spearman’s correlation coefficient; TG, triglyceride.

## Data Availability

The data are available within the article and Supplementary Materials. The raw data are available upon request.

## Supplementary Materials

The following supporting information can be downloaded at https://www.mdpi.com/article/10.3390/antiox13030339/s1, Table S1: Differences in demographic and clinical characteristics between HVs and patients with MS; Table S2: Differences in laboratory data between HVs and patients with MS; Table S3: Differences in serum levels and lipid contents of VLDL particles between HVs and patients with MS; Table S4: Differences in serum levels and lipid content of IDL particles between HVs and patients with MS; Table S5: Differences in serum levels of total LDL and LDL subclasses between HVs and patients with MS; Table S6: Differences in lipid contents of LDL particles between HVs and patients with MS; Table S7: Differences in serum levels of HDL subclasses between HVs and patients with MS; Table S8: Differences in lipid content of HDL particles between HVs and patients with MS; Table S9: Correlation analyses of AOPPs with the serum levels and lipid contents of VLDL in HVs and patients with MS; Table S10: Partial correlation analyses of AOPPs with the serum levels and lipid contents of VLDL in HVs; Table S11: Partial correlation analyses of AOPPs with the serum levels and lipid contents of VLDL in patients with MS; Table S12: Partial correlation analyses of AOPPs with the serum levels and lipid contents of IDL in HVs; Table S13: Partial correlation analyses of AOPPs with the serum levels and lipid contents of IDL in patients with MS; Table S14: Partial correlation analyses of AOPPs and lipid contents of LDL in HVs; Table S15: Partial correlation analyses of AOPPs with the serum levels and lipid contents of LDL in patients with MS; Table S16: Partial correlation analyses between AOPPs and the lipid contents of LDL subclasses in HVs; Table S17: Partial correlation analyses between AOPPs and the lipid contents of LDL subclasses in patients with MS; Table S18: Partial correlation analyses between AOPPs and the serum levels of total HDL and HDL subclasses in HVs; Table S19: Partial correlation analyses between AOPPs and the serum levels of total HDL and HDL subclasses in patients with MS; Table S20: Partial correlation analyses between AOPPs and the lipid contents of HDL subclasses in HVs; Table S21: Partial correlation analyses between AOPPs and the lipid contents of HDL subclasses in patients with MS; Table S22: Differences in serum levels and lipid contents of VLDL particles between HVs with low and high AOPPs; Table S23: Differences in serum levels and lipid contents of VLDL particles between patients with MS with low and high AOPPs; Table S24: Differences in serum levels and lipid contents of IDL particles between HVs with low and high AOPPs; Table S25: Differences in serum levels and lipid contents of IDL particles between patients with MS with low and high AOPPs; Table S26: Differences in serum levels of total LDL and LDL subclasses between HVs with low and high AOPPs; Table S27: Differences in serum levels of total LDL and LDL subclasses between patients with MS with low and high AOPPs; Table S28: Differences in lipid contents of LDL between HVs with low and high AOPPs; Table S29: Differences in lipid contents of LDL between patients with MS with low and high AOPPs; Table S30: Differences in serum levels of total HDL and HDL subclasses between HVs with low and high AOPPs; Table S31: Differences in serum levels of total HDL and HDL subclasses between patients with MS with low and high AOPPs; Table S32: Differences in lipid contents of total HDL and HDL subclasses between HVs with low and high AOPPs; Table S33: Differences in lipid contents of total HDL and HDL subclasses between patients with MS with low and high AOPPs; Table S34: VIP scores in HVs and patients with MS; Figure S1: Permutation validity test of OPLS-DA in (A) HVs and (B) patients with MS; Results S1: Lipoprotein parameters in HV and patients with MS with low and high AOPPs.
