# Supplementary material for: Advanced Oxidation Protein Products Are Strongly Associated with the Serum Levels and Lipid Contents of Lipoprotein Subclasses in Healthy Volunteers and Patients with Metabolic Syndrome
Source: Antioxidants (Basel). 2024 Mar 11;13(3):339. doi: 10.3390/antiox13030339 (PMC10968302; doi:10.3390/antiox13030339)
Supplement: Supplementary file 1 [file antioxidants-13-00339-s001.zip › Results S1.pdf]

Lipoprotein parameters in HV and patients with MS with low and high AOPPs

After a Bonferroni correction for multiple testing, the serum levels of cholesterol, free cholesterol, triglycerides, phospholipoids, and apoB in the total VLDL and VLDL subclasses 1-4, as well as in IDL were significantly higher in subjects with high, compared to those with low AOPPs in both HV and patients with MS (Tables S20 -S23). While the ratios indicating the cholesterol, free cholesterol, triglyceride, and phospholipid content of VLDL and IDL were similar in HV with high and low AOPPs, the cholesterol contents of VLDL as well as cholesterol, free cholesterol, triglyceride, and phospholipid contents of IDL were significantly higher in patients with MS with high compared to those with low AOPPs (Tables S21 and S23).

With exception of the serum levels of triglycerides and apoB in LDL subclass 5, all other indicators of the serum levels of cholesterol, free cholesterol, triglycerides, phospholipids, and apoB, in total LDL as well as in the LDL subclasses 1-6 were similar in HV with low and high AOPPs (Table S24). In patients with MS, serum levels of cholesterol, free cholesterol, triglycerides, phospholipids, and apoB in the LDL subclass 6 were significantly higher in patients with high compared to those with low AOPPs (Table S25). Also the serum levels of triglycerides in total LDL (LDL-TG) as well as in the LDL subclass 5 (LDL5-TG) were significantly higher in patients with MS with high compared to those with low AOPPs (Table S25).

Cholesterol (LDLC/LDL-apoB) and free cholesterol contents of total LDL as well as cholesterol content of the LDL subclass 2 and free cholesterol content of the LDL subclasses 4-6 were significantly higher in HV with low compared to HV with high AOPPs (Table S26). Additionally, phospholipid contents of total LDL, as well as of the LDL subclasses 2-6 were significantly higher, whereas triglyceride content of the LDL subclass 1 was significantly lower in HV with low compared to HV with high AOPPs (Table S26). In MS patients, cholesterol and free cholesterol contents of total LDL (LDL-C/LDL-apoB), as well as free cholesterol content of the LDL subclasses 5 and 6 were significantly higher in low compared to high AOPPs (Table S27). While triglyceride contents of total LDL and of the LDL subclasses were similar, phospholipid contents of total LDL and of the LDL subclasses 2-6 were significantly higher in patients with MS with low compared to the patients with high AOPPs (Table S27).

Serum levels of cholesterol, free cholesterol, phospholipids, and apoA-I in total HDL and the HDL subclasses 1 and 2 (as well as serum levels of cholesterol in the HDL subclass 3 and of apoA-II in the HDL subclass 1) were significantly higher in HV with low compared to those with high AOPPs, whereas serum levels of triglyceride in the HDL subclass 4 were significantly higher in HV with high compared to those with low AOPPs (Table S28). In patients with MS, serum levels of cholesterol in total HDL and in the HDL subclass 4 as well as serum levels of phospholipids in the HDL subclass 4 were significantly higher, whereas serum levels of triglycerides in the HDL subclasses 3 and 4 were significantly lower in MS patients with low compared to those with high AOPPs (Table S29).

While cholesterol and free cholesterol contents of total HDL were significantly higher, triglyceride contents of the HDL subclasses 1, 3, and 4 were significantly lower in the HV with low compared to the HV with high AOPPs (Table S30). The cholesterol contents of total HDL and HDL subclasses 3 and 4 as well as the phospholipid contents of HDL subclass 4 were significantly higher, whereas the triglyceride contents of total HDL and HDL subclasses 1-4 were significantly lower in patients with MS with low compared to the patients with high AOPPs (Table S31).
