# Supplementary material for: Advanced Oxidation Protein Products Are Strongly Associated with the Serum Levels and Lipid Contents of Lipoprotein Subclasses in Healthy Volunteers and Patients with Metabolic Syndrome
Source: Antioxidants (Basel). 2024 Mar 11;13(3):339. doi: 10.3390/antiox13030339 (PMC10968302; doi:10.3390/antiox13030339)
Supplement: Supplementary file 1 [file antioxidants-13-00339-s001.zip › Table S1.pdf]

Table S1. Differences in demographic and clinical characteristics between HV and patients with MS.

Variable	All (N=130)	HV (N=65)	MS (N=65)	p
Age (years)	56.0 (50.0, 60.0)	56.0 (50.0, 59.0)	57.0 (50.0, 60.0)	0.440
Sex (female)	62 (47.7%)	31 (47.7%)	31 (47.7%)	1.000
Body weight (kg)	87.5 (75.2, 102.8)	77.0 (68.0, 88.0)	98.0 (86.0, 113.5)	**<0.001**
Body height (m)	1.74 ± 0.10	1.75 ± 0.10	1.73 ± 0.11	0.243
BMI (kg/m^2)	28.8 (25.1, 32.7)	25.1 (23.7, 28.1)	32.6 (29.8, 35.9)	**<0.001**
Waist circumference (cm)	103.1 ± 16.5	92.2 ± 11.6	113.9 ± 13.2	**<0.001**
MAP (mmHg)	96.7 (88.3, 100.0)	88.3 (85.0, 96.7)	98.3 (96.7, 101.7)	**<0.001**
**Chronic diseases**				
Arterial hypertension	60 (46.2%)	0 (0.0%)	60 (92.3%)	**<0.001**
Diabetes mellitus type 2	27 (20.8%)	0 (0.0%)	27 (41.5%)	**<0.001**
Stable angina pectoris	2 (1.5%)	0 (0.0%)	2 (3.1%)	0.496
Atrial fibrillation	2 (1.5%)	0 (0.0%)	2 (3.1%)	0.496
CVI, TIA	1 (0.8%)	0 (0.0%)	1 (1.5%)	1.000
Intermittent claudications	4 (3.1%)	0 (0.0%)	4 (6.2%)	0.119
Deep venous thrombosis	6 (4.6%)	1 (1.5%)	5 (7.7%)	0.208
Pulmonary embolism	2 (1.5%)	0 (0.0%)	2 (3.1%)	0.496
**Functions and habits**				
Smoking	34 (26.2%)	16 (24.6%)	18 (27.7%)	0.842
Physical activity (≥3 times/week)	105 (80.8%)	58 (89.2%)	47 (72.3%)	**0.025**
Menstrual cycle (female)	18/62 (29.0%)	12/31 (38.7%)	6/31 (19.4%)	0.161

Data are presented as N (%), mean ± standard deviation, or median (q1, q3). Differences between HV and patients with MS were tested using Fisher's exact test, t test or Mann-Whitney U test. *p*-values < 0.05 are considered statistically significant and are depicted in bold. BMI, body mass index; cm, centimeter; CVI, cerebrovascular infarction; HV, healthy volunteer; kg, kilogram; m, meter; MAP, mean arterial pressure; MS, metabolic syndrome patient; N, number; TIA, transitory ischemic attack.
