# Supplementary material for: Advanced Oxidation Protein Products Are Strongly Associated with the Serum Levels and Lipid Contents of Lipoprotein Subclasses in Healthy Volunteers and Patients with Metabolic Syndrome
Source: Antioxidants (Basel). 2024 Mar 11;13(3):339. doi: 10.3390/antiox13030339 (PMC10968302; doi:10.3390/antiox13030339)
Supplement: Supplementary file 1 [file antioxidants-13-00339-s001.zip › Table S10.pdf]

Table S10. Partial correlation analyses of AOPPs with the serum levels and lipid content of VLDL in HV.

Variable	AOPPs (µmol/L)					
	Model 1		Model 2		Model 3	
	r	p	r	p	r	p
VLDL-C	0.69	**<0.0001**	0.68	**<0.0001**	0.70	**<0.0001**
VLDL1-C	0.64	**<0.0001**	0.65	**<0.0001**	0.66	**<0.0001**
VLDL2-C	0.68	**<0.0001**	0.67	**<0.0001**	0.69	**<0.0001**
VLDL3-C	0.71	**<0.0001**	0.71	**<0.0001**	0.73	**<0.0001**
VLDL4-C	0.69	**<0.0001**	0.68	**<0.0001**	0.69	**<0.0001**
VLDL5-C	0.14	0.2835	0.14	0.2712	0.13	0.3098
VLDL-FC	0.68	**<0.0001**	0.69	**<0.0001**	0.69	**<0.0001**
VLDL1-FC	0.71	**<0.0001**	0.71	**<0.0001**	0.72	**<0.0001**
VLDL2-FC	0.67	**<0.0001**	0.67	**<0.0001**	0.68	**<0.0001**
VLDL3-FC	0.68	**<0.0001**	0.68	**<0.0001**	0.70	**<0.0001**
VLDL4-FC	0.67	**<0.0001**	0.67	**<0.0001**	0.68	**<0.0001**
VLDL5-FC	0.31	0.0149	0.31	0.0137	0.30	0.0189
VLDL-TG	0.70	**<0.0001**	0.71	**<0.0001**	0.70	**<0.0001**
VLDL1-TG	0.65	**<0.0001**	0.67	**<0.0001**	0.65	**<0.0001**
VLDL2-TG	0.65	**<0.0001**	0.65	**<0.0001**	0.66	**<0.0001**
VLDL3-TG	0.66	**<0.0001**	0.66	**<0.0001**	0.66	**<0.0001**
VLDL4-TG	0.64	**<0.0001**	0.64	**<0.0001**	0.64	**<0.0001**
VLDL5-TG	0.12	0.3385	0.13	0.3043	0.13	0.3304
VLDL-PL	0.69	**<0.0001**	0.69	**<0.0001**	0.70	**<0.0001**
VLDL1-PL	0.65	**<0.0001**	0.66	**<0.0001**	0.66	**<0.0001**
VLDL2-PL	0.68	**<0.0001**	0.68	**<0.0001**	0.69	**<0.0001**
VLDL3-PL	0.69	**<0.0001**	0.69	**<0.0001**	0.71	**<0.0001**
VLDL4-PL	0.66	**<0.0001**	0.66	**<0.0001**	0.67	**<0.0001**
VLDL5-PL	0.29	0.0247	0.29	0.0223	0.30	0.0200
VLDL-apoB	0.70	**<0.0001**	0.70	**<0.0001**	0.70	**<0.0001**
VLDL-C/VLDL-apoB	0.35	0.0058	0.33	0.0106	0.36	0.0045
VLDL-FC/VLDL-apoB	-0.39	0.0020	-0.38	0.0022	-0.38	0.0023
VLDL-TG/VLDL-apoB	0.01	0.9553	0.03	0.8153	0.02	0.8509
VLDL-PL/VLDL-apoB	-0.25	0.0466	-0.24	0.0611	-0.22	0.0929

Spearman correlation analyses were used to evaluate associations between the serum levels of AOPPs and VLDL parameters. Model 1: Adjusted for age, sex, BMI. Model 2: Adjusted for age, sex, BMI, and CRP. Model 3: Adjusted for age, sex, BMI, and protein. *p*-values < 0.0003 are considered statistically significant after a Bonferroni correction for multiple comparison and are depicted in bold. Serum levels of lipids and apoB in VLDL are given in mg/dL. AOPPs, advanced oxidation protein products; apoB, apolipoprotein B; BMI, body mass index; C-cholesterol; CRP, C-reactive protein; FC-free cholesterol; HV, healthy volunteer; PL, phospholipid; r, Spearman's correlation coefficient; TG, triglyceride, VLDL, very low-density lipoprotein.
