# Supplementary material for: Advanced Oxidation Protein Products Are Strongly Associated with the Serum Levels and Lipid Contents of Lipoprotein Subclasses in Healthy Volunteers and Patients with Metabolic Syndrome
Source: Antioxidants (Basel). 2024 Mar 11;13(3):339. doi: 10.3390/antiox13030339 (PMC10968302; doi:10.3390/antiox13030339)
Supplement: Supplementary file 1 [file antioxidants-13-00339-s001.zip › Table S11.pdf]

Table S11. Partial correlation analyses of AOPPs with the serum levels and lipid content of VLDL in patients with MS.

	**AOPPs (μmol/L)**							
	**Model 1**		**Model 2**		**Model 3**		**Model 4**	
**Variable**	**r**	**p**	**r**	**p**	**r**	**p**	**r**	**p**
VLDL-C	0.80	**<0.0001**	0.80	**<0.0001**	0.80	**<0.0001**	0.80	**<0.0001**
VLDL1-C	0.78	**<0.0001**	0.78	**<0.0001**	0.79	**<0.0001**	0.78	**<0.0001**
VLDL2-C	0.79	**<0.0001**	0.79	**<0.0001**	0.80	**<0.0001**	0.79	**<0.0001**
VLDL3-C	0.81	**<0.0001**	0.81	**<0.0001**	0.81	**<0.0001**	0.80	**<0.0001**
VLDL4-C	0.74	**<0.0001**	0.74	**<0.0001**	0.74	**<0.0001**	0.73	**<0.0001**
VLDL5-C	0.04	0.7384	0.04	0.7363	0.04	0.7390	0.07	0.5992
VLDL-FC	0.80	**<0.0001**	0.80	**<0.0001**	0.81	**<0.0001**	0.81	**<0.0001**
VLDL1-FC	0.78	**<0.0001**	0.79	**<0.0001**	0.79	**<0.0001**	0.78	**<0.0001**
VLDL2-FC	0.81	**<0.0001**	0.81	**<0.0001**	0.81	**<0.0001**	0.81	**<0.0001**
VLDL3-FC	0.83	**<0.0001**	0.83	**<0.0001**	0.84	**<0.0001**	0.83	**<0.0001**
VLDL4-FC	0.74	**<0.0001**	0.74	**<0.0001**	0.74	**<0.0001**	0.74	**<0.0001**
VLDL5-FC	0.41	0.0009	0.41	0.0010	0.41	0.0010	0.44	0.0004
VLDL-TG	0.83	**<0.0001**	0.83	**<0.0001**	0.83	**<0.0001**	0.83	**<0.0001**
VLDL1-TG	0.80	**<0.0001**	0.80	**<0.0001**	0.80	**<0.0001**	0.80	**<0.0001**
VLDL2-TG	0.81	**<0.0001**	0.81	**<0.0001**	0.81	**<0.0001**	0.81	**<0.0001**
VLDL3-TG	0.82	**<0.0001**	0.82	**<0.0001**	0.82	**<0.0001**	0.81	**<0.0001**
VLDL4-TG	0.76	**<0.0001**	0.76	**<0.0001**	0.76	**<0.0001**	0.76	**<0.0001**
VLDL5-TG	0.29	0.0227	0.29	0.0241	0.29	0.0240	0.36	0.0048
VLDL-PL	0.81	**<0.0001**	0.81	**<0.0001**	0.81	**<0.0001**	0.81	**<0.0001**
VLDL1-PL	0.80	**<0.0001**	0.80	**<0.0001**	0.81	**<0.0001**	0.80	**<0.0001**
VLDL2-PL	0.82	**<0.0001**	0.82	**<0.0001**	0.82	**<0.0001**	0.82	**<0.0001**
VLDL3-PL	0.82	**<0.0001**	0.82	**<0.0001**	0.82	**<0.0001**	0.81	**<0.0001**
VLDL4-PL	0.75	**<0.0001**	0.75	**<0.0001**	0.75	**<0.0001**	0.75	**<0.0001**
VLDL5-PL	0.27	0.0328	0.27	0.0338	0.27	0.0344	0.30	0.0200
VLDL-apoB	0.82	**<0.0001**	0.82	**<0.0001**	0.82	**<0.0001**	0.83	**<0.0001**
VLDL-C/VLDL-apoB	0.60	**<0.0001**	0.60	**<0.0001**	0.63	**<0.0001**	0.59	**<0.0001**

VLDL-FC/VLDL-apoB	0.31	0.0156	0.31	0.0157	0.32	0.0113	0.30	0.0173
VLDL-TG/VLDL-apoB	0.46	**0.0002**	0.47	**0.0001**	0.47	**0.0001**	0.45	0.0003
VLDL-PL/VLDL-apoB	-0.01	0.9349	-0.01	0.9164	-0.01	0.9160	-0.08	0.5597

Spearman correlation analyses were used to evaluate the associations between the serum levels of AOPPs and VLDL parameters. Model 1: Adjusted for age, sex, BMI. Model 2: Adjusted for age, sex, BMI, and CRP. Model 3: Adjusted for age, sex, BMI, and protein. Model 4: Adjusted for age, sex, T2D, and statin. *p*-values < 0.0003 are considered statistically significant after a Bonferroni correction for multiple comparison and are depicted in bold. Serum levels of lipids and apoB in VLDL are given in mg/dL. AOPPs, advanced oxidation protein products; apoB, apolipoprotein B; BMI, body mass index; C-cholesterol; CRP, C-reactive protein; FC-free cholesterol; MS, metabolic syndrome; PL, phospholipid; r, Spearman's correlation coefficient; T2D, type 2 diabetes mellitus; TG, triglyceride; VLDL, very low-density lipoprotein.
