# Supplementary material for: Advanced Oxidation Protein Products Are Strongly Associated with the Serum Levels and Lipid Contents of Lipoprotein Subclasses in Healthy Volunteers and Patients with Metabolic Syndrome
Source: Antioxidants (Basel). 2024 Mar 11;13(3):339. doi: 10.3390/antiox13030339 (PMC10968302; doi:10.3390/antiox13030339)
Supplement: Supplementary file 1 [file antioxidants-13-00339-s001.zip › Table S12.pdf]

Table S12. Partial correlation analyses of AOPPs with the serum levels and lipid content of IDL in HV.

	AOPPs (µmol/L)					
	**Model 1**		**Model 2**		**Model 3**	
**Variable**	**r**	**p**	**r**	**p**	**r**	**p**
IDL-C	0.71	**<0.0001**	0.71	**<0.0001**	0.71	**<0.0001**
IDL-FC	0.73	**<0.0001**	0.72	**<0.0001**	0.73	**<0.0001**
IDL-TG	0.69	**<0.0001**	0.70	**<0.0001**	0.70	**<0.0001**
IDL-PL	0.70	**<0.0001**	0.69	**<0.0001**	0.70	**<0.0001**
IDL-apoB	0.68	**<0.0001**	0.67	**<0.0001**	0.66	**<0.0001**
IDL-C/IDL-apoB	0.36	0.0040	0.36	0.0041	0.37	0.0035
IDL-FC/IDL-apoB	0.49	**0.0001**	0.49	**0.0001**	0.49	**0.0001**
IDL-TG/IDL-apoB	0.40	0.0013	0.44	0.0004	0.42	0.0007
IDL-PL/IDL-apoB	-0.17	0.1970	-0.15	0.2429	-0.13	0.3358

Spearman correlation analyses were used to evaluate the associations between the serum levels of AOPPs and the serum levels of IDL parameters. Model 1: Adjusted for age, sex, BMI. Model 2: Adjusted for age, sex, BMI, and CRP. Model 3: Adjusted for age, sex, BMI, and protein. *p*-values < 0.0003 are considered statistically significant after a Bonferroni correction for multiple comparison and are depicted in bold. Serum levels of lipids and apoB in IDL are given in mg/dL. AOPPs, advanced oxidation protein products; apoB, apolipoprotein B; BMI, body mass index; C, cholesterol; CRP, C-reactive protein; FC, free cholesterol; HV, healthy volunteer; IDL, intermediate-density lipoprotein; PL, phospholipid; r, Spearman's correlation coefficient; TG, triglyceride.
