# Supplementary material for: Advanced Oxidation Protein Products Are Strongly Associated with the Serum Levels and Lipid Contents of Lipoprotein Subclasses in Healthy Volunteers and Patients with Metabolic Syndrome
Source: Antioxidants (Basel). 2024 Mar 11;13(3):339. doi: 10.3390/antiox13030339 (PMC10968302; doi:10.3390/antiox13030339)
Supplement: Supplementary file 1 [file antioxidants-13-00339-s001.zip › Table S14.pdf]

Table S14. Partial correlation analyses of AOPPs with the serum levels and lipid content of LDL in HV.

	AOPPs (µmol/L)					
	Model 1		Model 2		Model 3	
**Variable (mg/dL)**	**r**	**p**	**r**	**p**	**r**	**p**
LDL-C	0.24	0.0632	0.23	0.0805	0.20	0.1155
LDL1-C	0.05	0.6941	0.04	0.7705	-0.01	0.9390
LDL2-C	-0.26	0.0395	-0.27	0.0340	-0.30	0.0186
LDL3-C	-0.08	0.5617	-0.09	0.4851	-0.11	0.4157
LDL4-C	0.35	0.0048	0.34	0.0068	0.34	0.0074
LDL5-C	0.53	**<0.0001**	0.53	**<0.0001**	0.53	**<0.0001**
LDL6-C	0.46	**0.0002**	0.45	0.0003	0.43	0.0005
LDL-FC	0.14	0.2806	0.13	0.3085	0.10	0.4462
LDL1-FC	0.08	0.5286	0.06	0.6215	0.03	0.8376
LDL2-FC	-0.29	0.0235	-0.29	0.0216	-0.34	0.0076
LDL3-FC	-0.19	0.1494	-0.19	0.1377	-0.21	0.0974
LDL4-FC	0.22	0.0804	0.22	0.0958	0.21	0.1073
LDL5-FC	0.44	0.0003	0.44	0.0004	0.44	0.0004
LDL6-FC	0.35	0.0049	0.34	0.0068	0.33	0.0090
LDL-TG	0.55	**<0.0001**	0.54	**<0.0001**	0.55	**<0.0001**
LDL1-TG	0.51	**<0.0001**	0.50	**<0.0001**	0.48	**0.0001**
LDL2-TG	0.23	0.0743	0.21	0.1023	0.20	0.1220
LDL3-TG	0.05	0.6964	0.04	0.7645	0.01	0.9570
LDL4-TG	0.48	**0.0001**	0.46	**0.0002**	0.50	**<0.0001**
LDL5-TG	0.55	**<0.0001**	0.55	**<0.0001**	0.58	**<0.0001**
LDL6-TG	0.32	0.0124	0.31	0.0140	0.33	0.0087
LDL-PL	0.23	0.0770	0.21	0.1028	0.19	0.1366
LDL1-PL	0.07	0.6147	0.05	0.7202	0.00	0.9988
LDL2-PL	-0.26	0.0393	-0.28	0.0313	-0.29	0.0212
LDL3-PL	-0.08	0.5280	-0.10	0.4492	-0.11	0.4012
LDL4-PL	0.37	0.0034	0.35	0.0051	0.36	0.0048
LDL5-PL	0.51	**<0.0001**	0.51	**<0.0001**	0.52	**<0.0001**
LDL6-PL	0.39	0.0018	0.38	0.0022	0.37	0.0029
LDL-apoB	0.39	0.0017	0.38	0.0027	0.36	0.0040
LDL1-apoB	0.09	0.5047	0.07	0.6084	0.02	0.8622
LDL2-apoB	-0.19	0.1489	-0.20	0.1259	-0.23	0.0754
LDL3-apoB	-0.01	0.9417	-0.03	0.8405	-0.04	0.7746
LDL4-apoB	0.43	0.0004	0.42	0.0007	0.43	0.0005
LDL5-apoB	0.56	**<0.0001**	0.56	**<0.0001**	0.57	**<0.0001**
LDL6-apoB	0.52	**<0.0001**	0.52	**<0.0001**	0.50	**<0.0001**

Spearman correlation analyses were used to evaluate the associations between the serum levels of AOPPs and LDL parameters. Model 1: Adjusted for age, sex, BMI. Model 2: Adjusted for age, sex, BMI, and CRP. Model 3: Adjusted for age, sex, BMI, and protein. p-values < 0.0003 are considered statistically significant after a Bonferroni correction for multiple comparison and are depicted in bold. AOPPs, advanced oxidation protein products; apoB, apolipoprotein B; BMI, body mass index; C-

cholesterol; CRP, C-reactive protein; FC-free cholesterol; HV, healthy volunteer; PL, phospholipid; r, Spearman's correlation coefficient; TG, triglyceride; VLDL, very low-density lipoprotein.
