# Supplementary material for: Advanced Oxidation Protein Products Are Strongly Associated with the Serum Levels and Lipid Contents of Lipoprotein Subclasses in Healthy Volunteers and Patients with Metabolic Syndrome
Source: Antioxidants (Basel). 2024 Mar 11;13(3):339. doi: 10.3390/antiox13030339 (PMC10968302; doi:10.3390/antiox13030339)
Supplement: Supplementary file 1 [file antioxidants-13-00339-s001.zip › Table S15.pdf]

Table S15. Partial correlation analyses of AOPPs with the serum levels and lipid content of LDL in patients with MS.

	AOPPs (µmol/L)							
	**Model 1**		**Model 2**		**Model 3**		**Model 4**	
**Variable (mg/dL)**	**r**	**p**	**r**	**p**	**r**	**p**	**r**	**p**
LDL-C	0.01	0.9232	0.01	0.9219	0.01	0.9270	0.02	0.8664
LDL1-C	0.00	0.9970	0.00	0.9962	0.00	0.9978	0.03	0.8069
LDL2-C	-0.33	0.0098	-0.33	0.0106	-0.33	0.0102	-0.32	0.0117
LDL3-C	-0.32	0.0103	-0.32	0.0111	-0.33	0.0104	-0.32	0.0119
LDL4-C	-0.19	0.1290	-0.20	0.1309	-0.20	0.1274	-0.19	0.1356
LDL5-C	0.20	0.1126	0.20	0.1176	0.20	0.1157	0.19	0.1453
LDL6-C	0.72	**<0.0001**	0.72	**<0.0001**	0.72	**<0.0001**	0.73	**<0.0001**
LDL-FC	-0.08	0.5353	-0.08	0.5386	-0.08	0.5370	-0.07	0.5703
LDL1-FC	0.08	0.5334	0.08	0.5364	0.08	0.5337	0.12	0.3581
LDL2-FC	-0.25	0.0472	-0.25	0.0500	-0.25	0.0490	-0.22	0.0921
LDL3-FC	-0.29	0.0220	-0.29	0.0231	-0.29	0.0223	-0.26	0.0455
LDL4-FC	-0.21	0.1028	-0.21	0.1050	-0.21	0.1025	-0.19	0.1358
LDL5-FC	0.14	0.2794	0.14	0.2867	0.14	0.2841	0.13	0.3121
LDL6-FC	0.69	**<0.0001**	**0.69**	**<0.0001**	0.69	**<0.0001**	0.69	**<0.0001**
LDL-TG	0.65	**<0.0001**	**0.66**	**<0.0001**	0.65	**<0.0001**	0.68	**<0.0001**
LDL1-TG	0.58	**<0.0001**	**0.58**	**<0.0001**	0.58	**<0.0001**	0.64	**<0.0001**
LDL2-TG	0.22	0.0843	0.22	0.0818	0.22	0.0862	0.28	0.0294
LDL3-TG	-0.08	0.5242	-0.08	0.5408	-0.08	0.5282	-0.04	0.7337
LDL4-TG	0.25	0.0517	0.26	0.0471	0.25	0.0540	0.27	0.0382
LDL5-TG	0.62	**<0.0001**	**0.63**	**<0.0001**	0.62	**<0.0001**	0.65	**<0.0001**
LDL6-TG	0.71	**<0.0001**	**0.71**	**<0.0001**	0.71	**<0.0001**	0.70	**<0.0001**
LDL-PL	-0.05	0.7006	-0.05	0.7086	-0.05	0.6997	-0.04	0.7571
LDL1-PL	-0.02	0.9043	-0.02	0.9085	-0.02	0.9047	0.02	0.8724
LDL2-PL	-0.37	0.0033	-0.37	0.0036	-0.37	0.0036	-0.36	0.0046
LDL3-PL	-0.34	0.0070	-0.34	0.0076	-0.34	0.0071	-0.33	0.0090
LDL4-PL	-0.19	0.1447	-0.19	0.1466	-0.19	0.1422	-0.18	0.1563

LDL5-PL	0.17	0.1758	0.17	0.1819	0.17	0.1791	0.17	0.1998
LDL6-PL	0.70	**<0.0001**	0.70	**<0.0001**	0.70	**<0.0001**	0.71	**<0.0001**
LDL-apoB	0.30	0.0173	0.30	0.0179	0.30	0.0181	0.33	0.0089
LDL1-apoB	0.07	0.5835	0.07	0.5834	0.07	0.5847	0.11	0.4100
LDL2-apoB	-0.24	0.0619	-0.24	0.0650	-0.24	0.0631	-0.22	0.0814
LDL3-apoB	-0.28	0.0286	-0.28	0.0305	-0.28	0.0290	-0.27	0.0351
LDL4-apoB	-0.09	0.4709	-0.09	0.4723	-0.10	0.4641	-0.09	0.5032
LDL5-apoB	0.32	0.0119	0.32	0.0128	0.32	0.0125	0.31	0.0166
LDL6-apoB	0.76	**<0.0001**	0.76	**<0.0001**	0.76	**<0.0001**	0.77	**<0.0001**

Spearman correlation analyses were used to evaluate the associations between the serum levels of AOPPs and LDL parameters. Model 1: Adjusted for age, sex, BMI. Model 2: Adjusted for age, sex, BMI, and CRP. Model 3: Adjusted for age, sex, BMI, and protein. Model 4: Adjusted for age, sex, T2D, and statin. p-values <0.0003 are considered statistically significant after a Bonferroni correction for multiple comparison and are depicted in bold. AOPPs, advanced oxidation protein products; apoB, apolipoprotein B; BMI, body mass index; C-cholesterol; CRP, C-reactive protein; FC, free cholesterol; LDL, low-density lipoprotein; MS, metabolic syndrome; PL, phospholipid; r, Spearman's correlation coefficient; T2D, type 2 diabetes mellitus; TG, triglyceride.
