# Supplementary material for: Advanced Oxidation Protein Products Are Strongly Associated with the Serum Levels and Lipid Contents of Lipoprotein Subclasses in Healthy Volunteers and Patients with Metabolic Syndrome
Source: Antioxidants (Basel). 2024 Mar 11;13(3):339. doi: 10.3390/antiox13030339 (PMC10968302; doi:10.3390/antiox13030339)
Supplement: Supplementary file 1 [file antioxidants-13-00339-s001.zip › Table S16.pdf]

Table S16. Partial correlation analyses between AOPPs and the lipid content of the LDL subclasses in HV.

	AOPPs (µmol/L)					
	**Model 1**		**Model 2**		**Model 3**	
	**r**	**p**	**r**	**p**	**r**	**p**
LDL-C/LDL-apoB	-0.48	**0.0001**	-0.48	**0.0001**	-0.51	**<0.0001**
LDL1-C/LDL1-apoB	-0.20	0.1101	-0.19	0.1359	-0.22	0.0883
LDL2-C/LDL2-apoB	-0.51	**<0.0001**	-0.50	**<0.0001**	-0.54	**<0.0001**
LDL3-C/LDL3-apoB	-0.42	0.0006	-0.41	0.0010	-0.49	**0.0001**
LDL4-C/LDL4-apoB	-0.41	0.0008	-0.40	0.0014	-0.49	**0.0001**
LDL5-C/LDL5-apoB	-0.38	0.0024	-0.37	0.0035	-0.41	0.0012
LDL6-C/LDL6-apoB	-0.33	0.0079	-0.35	0.0058	-0.40	0.0016
LDL-FC/LDL-apoB	-0.61	**<0.0001**	-0.60	**<0.0001**	-0.63	**<0.0001**
LDL1-FC/LDL1-apoB	-0.18	0.1735	-0.17	0.1774	-0.17	0.2031
LDL2-FC/LDL2-apoB	-0.33	0.0084	-0.31	0.0149	-0.34	0.0077
LDL3-FC/LDL3-apoB	-0.47	**0.0001**	-0.45	**0.0003**	-0.49	**0.0001**
LDL4-FC/LDL4-apoB	-0.58	**<0.0001**	-0.57	**<0.0001**	-0.62	**<0.0001**
LDL5-FC/LDL5-apoB	-0.66	**<0.0001**	-0.65	**<0.0001**	-0.67	**<0.0001**
LDL6-FC/LDL6-apoB	-0.51	**<0.0001**	-0.52	**<0.0001**	-0.52	**<0.0001**
LDL-TG/LDL-apoB	0.38	0.0021	0.37	0.0030	0.43	0.0005
LDL1-TG/LDL1-apoB	0.55	**<0.0001**	0.56	**<0.0001**	0.56	**<0.0001**
LDL2-TG/LDL2-apoB	0.47	**0.0001**	0.45	0.0003	0.49	**0.0001**
LDL3-TG/LDL3-apoB	-0.02	0.8613	-0.01	0.9351	-0.03	0.8205
LDL4-TG/LDL4-apoB	0.28	0.0262	0.27	0.0346	0.32	0.0112
LDL5-TG/LDL5-apoB	0.24	0.0631	0.23	0.0746	0.31	0.0161
LDL6-TG/LDL6-apoB	-0.28	0.0300	-0.29	0.0240	-0.24	0.0589
LDL-PL/LDL-apoB	-0.64	**<0.0001**	-0.64	**<0.0001**	-0.64	**<0.0001**
LDL1-PL/LDL1-apoB	-0.37	0.0028	-0.36	0.0045	-0.38	0.0029
LDL2-PL/LDL2-apoB	-0.58	**<0.0001**	-0.57	**<0.0001**	-0.57	**<0.0001**
LDL3-PL/LDL3-apoB	-0.57	**<0.0001**	-0.56	**<0.0001**	-0.61	**<0.0001**
LDL4-PL/LDL4-apoB	-0.58	**<0.0001**	-0.57	**<0.0001**	-0.64	**<0.0001**
LDL5-PL/LDL5-apoB	-0.64	**<0.0001**	-0.63	**<0.0001**	-0.63	**<0.0001**
LDL6-PL/LDL6-apoB	-0.67	**<0.0001**	-0.68	**<0.0001**	-0.66	**<0.0001**

Spearman correlation analyses were used to evaluate the associations between the serum levels of AOPPs and LDL parameters. Model 1: Adjusted for age, sex, BMI. Model 2: Adjusted for age, sex, BMI, and CRP. Model 3: Adjusted for age, sex, BMI, and protein. *p*-values < 0.0003 are considered statistically significant after a Bonferroni correction for multiple comparison and are depicted in bold. AOPPs, advanced oxidation protein products; apoB, apolipoprotein B; BMI, body mass index; C-cholesterol; CRP, C-reactive protein; FC, free cholesterol; HV, healthy volunteer; LDL, low-density lipoprotein; PL, phospholipid; r, Spearman's correlation coefficient; TG, triglyceride.
