# Supplementary material for: Advanced Oxidation Protein Products Are Strongly Associated with the Serum Levels and Lipid Contents of Lipoprotein Subclasses in Healthy Volunteers and Patients with Metabolic Syndrome
Source: Antioxidants (Basel). 2024 Mar 11;13(3):339. doi: 10.3390/antiox13030339 (PMC10968302; doi:10.3390/antiox13030339)
Supplement: Supplementary file 1 [file antioxidants-13-00339-s001.zip › Table S17.pdf]

Table S17. Partial correlation analyses between AOPPs and the lipid content of LDL subclasses in patients with MS.

	AOPPs (μmol/L)							
	**Model 1**		**Model 2**		**Model 3**		**Model 4**	
**Variable**	**r**	**p**	**r**	**p**	**r**	**p**	**r**	**p**
LDL-C/LDL-apoB	-0.66	**<0.0001**	-0.67	**<0.0001**	-0.66	**<0.0001**	-0.67	**<0.0001**
LDL1-C/LDL1-apoB	-0.23	0.0684	-0.23	0.0684	-0.24	0.0644	-0.25	0.0500
LDL2-C/LDL2-apoB	-0.40	0.0014	-0.40	0.0014	-0.40	0.0014	-0.41	0.0010
LDL3-C/LDL3-apoB	-0.50	**<0.0001**	-0.50	**<0.0001**	-0.51	**<0.0001**	-0.50	**<0.0001**
LDL4-C/LDL4-apoB	-0.52	**<0.0001**	-0.52	**<0.0001**	-0.51	**<0.0001**	-0.49	**0.0001**
LDL5-C/LDL5-apoB	-0.46	**0.0001**	-0.48	**0.0001**	-0.47	**0.0002**	-0.46	**0.0002**
LDL6-C/LDL6-apoB	-0.38	0.0023	-0.38	0.0025	-0.38	0.0024	-0.40	0.0013
LDL-FC/LDL-apoB	**-0.70**	**<0.0001**	-0.72	**<0.0001**	-0.70	**<0.0001**	-0.70	**<0.0001**
LDL1-FC/LDL1-apoB	-0.16	0.2053	-0.18	0.1715	-0.16	0.2082	-0.16	0.2061
LDL2-FC/LDL2-apoB	-0.03	0.8156	-0.04	0.7818	-0.03	0.8216	0.01	0.9195
LDL3-FC/LDL3-apoB	-0.09	0.4745	-0.10	0.4393	-0.09	0.4813	0.00	0.9979
LDL4-FC/LDL4-apoB	-0.29	0.0262	-0.29	0.0274	-0.31	0.0195	-0.28	0.0305
LDL5-FC/LDL5-apoB	-0.76	**<0.0001**	-0.76	**<0.0001**	-0.76	**<0.0001**	-0.75	**<0.0001**
LDL6-FC/LDL6-apoB	-0.75	**<0.0001**	-0.75	**<0.0001**	-0.75	**<0.0001**	-0.76	**<0.0001**
LDL-TG/LDL-apoB	0.41	0.0009	0.41	0.0009	0.42	0.0009	0.43	0.0005
LDL1-TG/LDL1-apoB	0.52	**<0.0001**	0.52	**<0.0001**	0.53	**<0.0001**	0.56	**<0.0001**
LDL2-TG/LDL2-apoB	0.46	**0.0002**	0.46	**0.0002**	0.46	**0.0002**	0.49	**0.0001**
LDL3-TG/LDL3-apoB	0.32	0.0101	0.33	0.0098	0.33	0.0100	0.35	0.0059
LDL4-TG/LDL4-apoB	0.39	0.0025	0.40	0.0019	0.38	0.0030	0.40	0.0020
LDL5-TG/LDL5-apoB	0.49	**0.0001**	0.51	**<0.0001**	0.49	**0.0001**	0.52	**<0.0001**
LDL6-TG/LDL6-apoB	-0.21	0.1032	-0.21	0.1076	-0.21	0.1065	-0.23	0.0789
LDL-PL/LDL-apoB	-0.81	**<0.0001**	-0.82	**<0.0001**	-0.81	**<0.0001**	-0.82	**<0.0001**
LDL1-PL/LDL1-apoB	-0.54	**<0.0001**	-0.55	**<0.0001**	-0.55	**<0.0001**	-0.55	**<0.0001**
LDL2-PL/LDL2-apoB	-0.62	**<0.0001**	-0.63	**<0.0001**	-0.63	**<0.0001**	-0.63	**<0.0001**
LDL3-PL/LDL3-apoB	-0.69	**<0.0001**	-0.70	**<0.0001**	-0.70	**<0.0001**	-0.69	**<0.0001**
LDL4-PL/LDL4-apoB	-0.68	**<0.0001**	-0.68	**<0.0001**	-0.68	**<0.0001**	-0.66	**<0.0001**

LDL5-PL/LDL5-apoB	-0.83	**<0.0001**	-0.83	**<0.0001**	-0.83	**<0.0001**	-0.82	**<0.0001**
LDL6-PL/LDL6-apoB	-0.78	**<0.0001**	-0.79	**<0.0001**	-0.78	**<0.0001**	-0.80	**<0.0001**

Spearman correlation analyses were used to evaluate the associations between the serum levels of AOPPs and LDL parameters. Model 1: Adjusted for age, sex, BMI. Model 2: Adjusted for age, sex, BMI, and CRP. Model 3: Adjusted for age, sex, BMI, and protein. Model 4: Adjusted for age, sex, T2D, and statin. *p*-values < 0.0003 are considered statistically significant after a Bonferroni correction for multiple comparison and are depicted in bold. AOPPs, advanced oxidation protein products; apoB, apolipoprotein B; BMI, body mass index; C-cholesterol; CRP, C-reactive protein; FC, free cholesterol; LDL, low-density lipoprotein; MS, metabolic syndrome; PL, phospholipid; r, Spearman's correlation coefficient; T2D, type 2 diabetes mellitus; TG, triglyceride.
