# Supplementary material for: Advanced Oxidation Protein Products Are Strongly Associated with the Serum Levels and Lipid Contents of Lipoprotein Subclasses in Healthy Volunteers and Patients with Metabolic Syndrome
Source: Antioxidants (Basel). 2024 Mar 11;13(3):339. doi: 10.3390/antiox13030339 (PMC10968302; doi:10.3390/antiox13030339)
Supplement: Supplementary file 1 [file antioxidants-13-00339-s001.zip › Table S18.pdf]

Table S18. Partial correlation analyses between AOPPs and the serum levels of total HDL and HDL subclasses in HV.

	**AOPPs (µmol/L)**					
	**Model 1**		**Model 2**		**Model 3**	
**Variable (mg/dL)**	**r**	**p**	**r**	**p**	**r**	**p**
HDL-C	-0.54	**<0.0001**	-0.54	**<0.0001**	-0.58	**<0.0001**
HDL1-C	-0.50	**<0.0001**	-0.49	**0.0001**	-0.51	**<0.0001**
HDL2-C	-0.50	**<0.0001**	-0.51	**<0.0001**	-0.49	**0.0001**
HDL3-C	-0.35	0.0060	-0.35	0.0056	-0.34	0.0072
HDL4-C	-0.02	0.8592	-0.01	0.9372	-0.12	0.3691
HDL-FC	-0.55	**<0.0001**	-0.55	**<0.0001**	-0.57	**<0.0001**
HDL1-FC	-0.54	**<0.0001**	-0.53	**<0.0001**	-0.55	**<0.0001**
HDL2-FC	-0.45	0.0003	-0.47	**0.0001**	-0.43	0.0005
HDL3-FC	-0.11	0.3982	-0.12	0.3636	-0.15	0.2567
HDL4-FC	0.08	0.5502	0.08	0.5372	0.01	0.9252
HDL-TG	0.40	0.0014	0.40	0.0015	0.43	0.0006
HDL1-TG	0.00	0.9734	0.01	0.9301	0.05	0.6807
HDL2-TG	0.34	0.0066	0.34	0.0080	0.40	0.0014
HDL3-TG	0.54	**<0.0001**	0.54	**<0.0001**	0.59	**<0.0001**
HDL4-TG	0.61	**<0.0001**	0.62	**<0.0001**	0.61	**<0.0001**
HDL-PL	-0.51	**<0.0001**	-0.52	**<0.0001**	-0.51	**<0.0001**
HDL1-PL	-0.51	**<0.0001**	-0.51	**<0.0001**	-0.50	**<0.0001**
HDL2-PL	-0.43	0.0006	-0.44	0.0004	-0.39	0.0017
HDL3-PL	-0.20	0.1119	-0.22	0.0951	-0.18	0.1661
HDL4-PL	0.04	0.7344	0.04	0.7559	-0.01	0.9136
HDL-apoA-I	-0.40	0.0013	-0.39	0.0017	-0.42	0.0007
HDL1-apoA-I	-0.50	**<0.0001**	-0.49	**0.0001**	-0.49	**0.0001**
HDL2-apoA-I	-0.48	**0.0001**	-0.48	**0.0001**	-0.47	**0.0001**
HDL3-apoA-I	-0.20	0.1125	-0.21	0.1009	-0.19	0.1324
HDL4-apoA-I	0.14	0.2914	0.15	0.2561	0.06	0.6450
HDL-apoA-II	0.06	0.6416	0.05	0.6897	0.02	0.8664
HDL1-apoA-II	-0.42	0.0007	-0.42	0.0009	-0.38	0.0022
HDL2-apoA-II	-0.13	0.3238	-0.13	0.3083	-0.07	0.5807
HDL3-apoA-II	0.25	0.0536	0.24	0.0661	0.28	0.0317
HDL4-apoA-II	0.22	0.0926	0.22	0.0823	0.14	0.2704

Spearman correlation analyses were used to evaluate the associations between the serum levels of AOPPs and the serum levels of lipids and apolipoproteins in total HDL and HDL subclasses in HV. Model 1: Adjusted for age, sex, BMI. Model 2: Adjusted for age, sex, BMI, and CRP. Model 3: Adjusted for age, sex, BMI, and protein. P-values <0.0003 are considered statistically significant after a Bonferroni correction for multiple comparison and are depicted in bold. AOPPs, advanced oxidation protein products; apoA-I, apolipoprotein A-I; apoA-II, apolipoprotein A-II; BMI, body mass index; CRP, C-reactive protein; HDL, high-density lipoprotein; HV, healthy volunteer; PL, phospholipid; r, Spearman's correlation coefficient; TG, triglyceride.
