# Supplementary material for: Advanced Oxidation Protein Products Are Strongly Associated with the Serum Levels and Lipid Contents of Lipoprotein Subclasses in Healthy Volunteers and Patients with Metabolic Syndrome
Source: Antioxidants (Basel). 2024 Mar 11;13(3):339. doi: 10.3390/antiox13030339 (PMC10968302; doi:10.3390/antiox13030339)
Supplement: Supplementary file 1 [file antioxidants-13-00339-s001.zip › Table S19.pdf]

Table S19. Partial correlation analyses between AOPPs and the serum levels of total HDL and HDL subclasses in patients with MS.

	AOPPs (μmol/L)							
	**Model 1**		**Model 2**		**Model 3**		**Model 4**	
**Variable (mg/dL)**	**r**	**p**	**r**	**p**	**r**	**p**	**r**	**p**
HDL-C	-0.63	**<0.0001**	-0.65	**<0.0001**	-0.63	**<0.0001**	-0.62	**<0.0001**
HDL1-C	-0.02	0.8471	-0.03	0.8411	-0.02	0.8559	0.05	0.7235
HDL2-C	-0.30	0.0196	-0.30	0.0203	-0.30	0.0197	-0.25	0.0547
HDL3-C	-0.45	**0.0002**	-0.46	**0.0002**	-0.46	**0.0002**	-0.44	0.0004
HDL4-C	-0.55	**<0.0001**	-0.57	**<0.0001**	-0.55	**<0.0001**	-0.55	**<0.0001**
HDL-FC	-0.44	0.0003	-0.45	0.0003	-0.45	0.0003	-0.39	0.0019
HDL1-FC	-0.21	0.0957	-0.22	0.0903	-0.22	0.0937	-0.16	0.2104
HDL2-FC	-0.33	0.0085	-0.33	0.0087	-0.34	0.0065	-0.33	0.0085
HDL3-FC	-0.40	0.0013	-0.40	0.0012	-0.41	0.0011	-0.42	0.0008
HDL4-FC	-0.40	0.0012	-0.41	0.0009	-0.41	0.0012	-0.43	0.0006
HDL-TG	0.50	**<0.0001**	0.50	**<0.0001**	0.50	**<0.0001**	0.53	**<0.0001**
HDL1-TG	0.48	**0.0001**	0.48	**0.0001**	0.48	**0.0001**	0.52	**<0.0001**
HDL2-TG	0.47	**0.0001**	0.47	**0.0001**	0.47	**0.0001**	0.49	**0.0001**
HDL3-TG	0.47	**0.0001**	0.47	**0.0001**	0.47	**0.0001**	0.49	**0.0001**
HDL4-TG	0.55	**<0.0001**	0.55	**<0.0001**	0.55	**<0.0001**	0.60	**<0.0001**
HDL-PL	-0.55	**<0.0001**	-0.55	**<0.0001**	-0.55	**<0.0001**	-0.54	**<0.0001**
HDL1-PL	-0.15	0.2606	-0.14	0.2657	-0.14	0.2657	-0.09	0.5032
HDL2-PL	-0.22	0.0893	-0.22	0.0919	-0.22	0.0859	-0.18	0.1699
HDL3-PL	-0.42	0.0007	-0.42	0.0007	-0.42	0.0007	-0.43	0.0006
HDL4-PL	-0.60	**<0.0001**	-0.61	**<0.0001**	-0.60	**<0.0001**	-0.61	**<0.0001**
HDL-apoA-I	-0.44	0.0003	-0.46	**0.0002**	-0.44	0.0003	-0.42	0.0007
HDL1-apoA-I	-0.10	0.4454	-0.10	0.4450	-0.10	0.4517	-0.03	0.8360
HDL2-apoA-I	-0.29	0.0201	-0.30	0.0195	-0.30	0.0193	-0.25	0.0562
HDL3-apoA-I	-0.23	0.0742	-0.23	0.0710	-0.24	0.0643	-0.19	0.1446
HDL4-apoA-I	-0.45	0.0003	-0.47	**0.0001**	-0.45	0.0003	-0.44	0.0003
HDL-apoA-II	-0.12	0.3406	-0.14	0.2895	-0.13	0.3300	-0.13	0.3346

HDL1-apoA-II	0.01	0.9133	0.01	0.9151	0.01	0.9264	0.04	0.7506
HDL2-apoA-II	0.10	0.4504	0.10	0.4576	0.10	0.4548	0.08	0.5180
HDL3-apoA-II	0.07	0.5696	0.07	0.5853	0.07	0.5749	0.08	0.5354
HDL4-apoA-II	-0.34	0.0067	-0.37	0.0036	-0.34	0.0072	-0.37	0.0032

Spearman correlation analyses were used to evaluate the associations between the serum levels of AOPPs and the serum levels of lipids and apolipoproteins in total HDL and HDL subclasses in patients with MS. Model 1: Adjusted for age, sex, BMI. Model 2: Adjusted for age, sex, BMI, and CRP. Model 3: Adjusted for age, sex, BMI, and protein. Model 4: Adjusted for age, sex, T2D, and statin. p-values < 0.0003 are considered statistically significant after a Bonferroni correction for multiple comparison and are depicted in bold. AOPPs, advanced oxidation protein products; apoA-I, apolipoprotein A-I; apoA-II, apolipoprotein A-II; BMI, body mass index; CRP, C-reactive protein; HDL, high-density lipoprotein; MS, metabolic syndrome; PL, phospholipid; r, Spearman's correlation coefficient; T2D; type 2 diabetes mellitus; TG, triglyceride.
