# Supplementary material for: Advanced Oxidation Protein Products Are Strongly Associated with the Serum Levels and Lipid Contents of Lipoprotein Subclasses in Healthy Volunteers and Patients with Metabolic Syndrome
Source: Antioxidants (Basel). 2024 Mar 11;13(3):339. doi: 10.3390/antiox13030339 (PMC10968302; doi:10.3390/antiox13030339)
Supplement: Supplementary file 1 [file antioxidants-13-00339-s001.zip › Table S2.pdf]

Table S2. Differences in laboratory data between HV and patients with MS.

Variable	All (N=130)	HV (N=65)	MS (N=65)	p
AOPPs (μmol/L)	37.8 (29.9, 46.9)	34.6 (28.9, 42.6)	41.6 (30.9, 57.3)	**0.004**
Triglycerides (mmol/L)	1.3 (0.9, 1.9)	1.0 (0.8, 1.4)	1.6 (1.1, 2.2)	**<0.001**
Total cholesterol (mmol/L)	5.3 (4.7, 6.1)	5.5 (5.1, 6.0)	5.0 (4.3, 6.2)	0.057
LDL-C (mmol/L)	3.2 (2.5, 3.7)	3.3 (2.8, 3.7)	3.0 (2.3, 3.7)	0.077
HDL-C (mmol/L)	1.4 (1.1, 1.7)	1.6 (1.4, 1.8)	1.2 (1.0, 1.4)	**<0.001**
Glucose (mmol/L)	5.3 (4.9, 5.7)	4.9 (4.8, 5.2)	5.7 (5.3, 6.5)	**<0.001**
Protein (g/L)	73.0 (70.0, 76.0)	72.0 (69.0, 75.0)	75.0 (71.0, 77.0)	**0.002**
Albumin (g/L)	48.0 (46.0, 49.0)	47.0 (46.0, 49.0)	48.0 (45.0, 49.0)	0.465
CRP (μg/mL)	1.8 (0.8, 3.7)	1.2 (0.6, 2.3)	2.4 (1.2, 5.5)	**<0.001**
IL-6 (pg/mL)	3.0 (2.1, 5.3)	2.3 (1.7, 3.0)	4.1 (2.7, 6.8)	**<0.001**
Bilirubin (μmol/L)	8.5 (6.0, 11.6)	9.6 (7.4, 13.3)	7.4 (5.5, 10.4)	**0.012**
AST (U/L)	23.0 (20.0, 27.0)	23.0 (20.0, 25.0)	23.0 (19.0, 32.0)	0.244
ALT (U/L)	24.0 (19.0, 36.0)	22.0 (18.0, 29.0)	30.0 (22.0, 43.0)	**<0.001**
AP (U/L)	61.0 (51.0, 73.0)	60.0 (49.0, 70.0)	65.0 (52.0, 81.0)	0.065
GGT (U/L)	24.5 (15.2, 38.0)	16.0 (13.0, 30.0)	31.0 (21.0, 44.0)	**<0.001**
CK (U/L)	124.5 (83.0, 186.8)	115.0 (81.0, 153.0)	133.0 (86.0, 226.0)	**0.048**
LDH (U/L)	172.0 (150.5, 192.0)	168.0 (147.0, 191.0)	176.0 (158.0, 193.0)	0.365
Urea (mmol/L)	5.3 (4.5, 6.3)	5.0 (4.2, 6.0)	5.6 (4.8, 6.5)	**0.004**
Urate (μmol/L)	297.5 (249.9, 345.1)	273.7 (232.0, 327.2)	315.3 (279.7, 362.9)	**<0.001**
Creatinine (μmol/L)	77.9 (67.3, 87.6)	77.9 (69.0, 89.4)	76.6 (65.5, 87.0)	0.414
eGFR (mL/min/1.73 m^2)	88.0 (78.0, 97.1)	87.5 (77.2, 93.6)	88.9 (79.1, 98.0)	0.358
Sodium (mmol/L)	139.0 (138.0, 141.0)	140.0 (138.0, 141.0)	139.0 (138.0, 140.0)	**0.041**
Potassium (mmol/L)	4.2 (4.1, 4.6)	4.3 (4.1, 4.5)	4.2 (4.1, 4.6)	0.703
Chloride (mmol/L)	100.0 (98.2, 102.8)	101.0 (99.0, 103.0)	100.0 (98.0, 101.0)	**0.006**

Data are presented as median (q1, q3). Differences between HV and patients with MS were tested using the Mann-Whitney U test. *p*-values < 0.05 are considered statistically significant and are depicted in bold. LDL-C and eGFR data were available for 60 and 64 MS patients, respectively. ALT, alanine aminotransferase; AP, alkaline phosphatase; AST, aspartate aminotransferase; CK, creatine kinase; CRP, C-reactive protein; eGFR, estimated glomerular filtration rate; EL, endothelial lipase; g, gram; GGT, gamma-glutamyl transpeptidase; HV, healthy volunteer; HDL-C, high-density lipoprotein cholesterol; IL-6, interleukin 6; LDH, lactate dehydrogenase; LDL-C, low-density lipoprotein cholesterol; MS, metabolic syndrome patient.
