# Supplementary material for: Advanced Oxidation Protein Products Are Strongly Associated with the Serum Levels and Lipid Contents of Lipoprotein Subclasses in Healthy Volunteers and Patients with Metabolic Syndrome
Source: Antioxidants (Basel). 2024 Mar 11;13(3):339. doi: 10.3390/antiox13030339 (PMC10968302; doi:10.3390/antiox13030339)
Supplement: Supplementary file 1 [file antioxidants-13-00339-s001.zip › Table S20.pdf]

Table S20. Partial correlation analyses between AOPPs and the lipid content of HDL subclasses in HV.

	AOPPs (µmol/L)					
	Model 1		Model 2		Model 3	
**Variable**	**r**	**p**	**r**	**p**	**r**	**p**
HDL-C / HDL-apoA-I	-0.58	**<0.0001**	-0.58	**<0.0001**	-0.61	**<0.0001**
HDL1-C / HDL1-apoA-I	0.30	0.0193	0.28	0.0272	0.23	0.0765
HDL2-C / HDL2-apoA-I	-0.27	0.0320	-0.27	0.0333	-0.27	0.0360
HDL3-C / HDL3-apoA-I	-0.43	0.0004	-0.44	0.0004	-0.45	0.0003
HDL4-C / HDL4-apoA-I	-0.39	0.0019	-0.38	0.0023	-0.47	0.0001
HDL-FC / HDL-apoA-I	-0.56	**<0.0001**	-0.55	**<0.0001**	-0.56	**<0.0001**
HDL1-FC / HDL1-apoA-I	0.30	0.0166	0.30	0.0169	0.26	0.0445
HDL2-FC / HDL2-apoA-I	0.04	0.7422	0.01	0.9165	0.04	0.7808
HDL3-FC / HDL3-apoA-I	0.04	0.7710	0.03	0.8039	-0.05	0.6876
HDL4-FC / HDL4-apoA-I	0.03	0.8331	0.02	0.8843	0.00	0.9984
HDL-TG / HDL-apoA-I	0.46	**0.0002**	0.46	**0.0002**	0.52	**<0.0001**
HDL1-TG / HDL1-apoA-I	0.58	**<0.0001**	0.59	**<0.0001**	0.62	**<0.0001**
HDL2-TG / HDL2-apoA-I	0.51	**<0.0001**	0.50	**<0.0001**	0.57	**<0.0001**
HDL3-TG / HDL3-apoA-I	0.58	**<0.0001**	0.58	**<0.0001**	0.63	**<0.0001**
HDL4-TG / HDL4-apoA-I	0.58	**<0.0001**	0.58	**<0.0001**	0.62	**<0.0001**
HDL-PL / HDL-apoA-I	-0.48	**0.0001**	-0.50	**<0.0001**	-0.43	0.0005
HDL1-PL / HDL1-apoA-I	0.35	0.0051	0.33	0.0104	0.35	0.0056
HDL2-PL / HDL2-apoA-I	-0.20	0.1227	-0.23	0.0763	-0.13	0.3024
HDL3-PL / HDL3-apoA-I	-0.11	0.4078	-0.14	0.2955	-0.06	0.6677
HDL4-PL / HDL4-apoA-I	-0.21	0.1009	-0.22	0.0949	-0.17	0.1919

Spearman correlation analyses were used to evaluate the associations between the serum levels of AOPPs and ratios indicating lipid content of HDL subclasses in HV. Model 1: Adjusted for age, sex, BMI. Model 2: Adjusted for age, sex, BMI, and CRP. Model 3: Adjusted for age, sex, BMI, and protein. *p*-values < 0.0003 are considered statistically significant after a Bonferroni correction for multiple comparison and are depicted in bold. AOPPs, advanced oxidation protein products; apoA-I, apolipoprotein A-I; BMI, body mass index; CRP, C-reactive protein; HDL, high-density lipoprotein; HV, healthy volunteer; PL, phospholipid; r, Spearman's correlation coefficient; TG, triglyceride.
