# Supplementary material for: Advanced Oxidation Protein Products Are Strongly Associated with the Serum Levels and Lipid Contents of Lipoprotein Subclasses in Healthy Volunteers and Patients with Metabolic Syndrome
Source: Antioxidants (Basel). 2024 Mar 11;13(3):339. doi: 10.3390/antiox13030339 (PMC10968302; doi:10.3390/antiox13030339)
Supplement: Supplementary file 1 [file antioxidants-13-00339-s001.zip › Table S21.pdf]

Table S21. Partial correlation analyses between AOPPs and the lipid content of HDL subclasses in patients with MS.

	AOPPs (μmol/L)							
	**Model 1**		**Model 2**		**Model 3**		**Model 4**	
**Variable**	**r**	**p**	**r**	**p**	**r**	**p**	**r**	**p**
HDL-C / HDL-apoA-I	-0.55	**<0.0001**	-0.56	**<0.0001**	-0.56	**<0.0001**	-0.57	**<0.0001**
HDL1-C / HDL1-apoA-I	0.19	0.1378	0.19	0.1433	0.19	0.1402	0.20	0.1228
HDL2-C / HDL2-apoA-I	0.02	0.8886	0.02	0.8798	0.02	0.8890	0.01	0.9186
HDL3-C / HDL3-apoA-I	-0.51	**<0.0001**	-0.51	**<0.0001**	-0.51	**<0.0001**	-0.54	**<0.0001**
HDL4-C / HDL4-apoA-I	-0.63	**<0.0001**	-0.65	**<0.0001**	-0.63	**<0.0001**	-0.66	**<0.0001**
HDL-FC / HDL-apoA-I	-0.26	0.0425	-0.26	0.0447	-0.26	0.0409	-0.17	0.1843
HDL1-FC / HDL1-apoA-I	-0.14	0.2930	-0.14	0.2938	-0.14	0.2723	-0.17	0.1958
HDL2-FC / HDL2-apoA-I	-0.05	0.6989	-0.05	0.6978	-0.05	0.6890	-0.13	0.3350
HDL3-FC / HDL3-apoA-I	-0.20	0.1235	-0.20	0.1222	-0.20	0.1217	-0.25	0.0522
HDL4-FC / HDL4-apoA-I	-0.29	0.0214	-0.29	0.0226	-0.30	0.0205	-0.33	0.0101
HDL-TG / HDL-apoA-I	0.67	**<0.0001**	0.67	**<0.0001**	0.67	**<0.0001**	0.68	**<0.0001**
HDL1-TG / HDL1-apoA-I	0.71	**<0.0001**	0.72	**<0.0001**	0.72	**<0.0001**	0.72	**<0.0001**
HDL2-TG / HDL2-apoA-I	0.70	**<0.0001**	0.71	**<0.0001**	0.70	**<0.0001**	0.72	**<0.0001**
HDL3-TG / HDL3-apoA-I	0.73	**<0.0001**	0.74	**<0.0001**	0.73	**<0.0001**	0.74	**<0.0001**
HDL4-TG / HDL4-apoA-I	0.68	**<0.0001**	0.69	**<0.0001**	0.68	**<0.0001**	0.70	**<0.0001**
HDL-PL / HDL-apoA-I	-0.46	**0.0002**	-0.46	**0.0002**	-0.46	**0.0002**	-0.47	**0.0001**
HDL1-PL / HDL1-apoA-I	-0.13	0.3000	-0.13	0.3116	-0.13	0.3003	-0.19	0.1409
HDL2-PL / HDL2-apoA-I	0.07	0.5814	0.08	0.5416	0.07	0.5886	0.04	0.7441
HDL3-PL / HDL3-apoA-I	-0.32	0.0110	-0.32	0.0117	-0.32	0.0117	-0.47	**0.0001**
HDL4-PL / HDL4-apoA-I	-0.67	**<0.0001**	-0.67	**<0.0001**	-0.68	**<0.0001**	-0.72	**<0.0001**

Partial spearman correlation analyses were used to evaluate the associations between the serum levels of AOPPs and ratios indicating lipid content of HDL subclasses in patients with MS. Model 1: Adjusted for age, sex, BMI. Model 2: Adjusted for age, sex, BMI, and CRP. Model 3: Adjusted for age, sex, BMI, and protein. Model 4: Adjusted for age, sex, statin, and T2D. P-values <0.0003 are considered statistically significant after a Bonferroni correction for multiple

comparison and are depicted in bold. AOPPs, advanced oxidation protein products; apoA-I, apolipoprotein A-I; BMI, body mass index; CRP, C-reactive protein; HDL, high-density lipoprotein; MS, metabolic syndrome; PL, phospholipid; r, Spearman's correlation coefficient; T2D, type 2 diabetes mellitus; TG, triglyceride.
