# Supplementary material for: Advanced Oxidation Protein Products Are Strongly Associated with the Serum Levels and Lipid Contents of Lipoprotein Subclasses in Healthy Volunteers and Patients with Metabolic Syndrome
Source: Antioxidants (Basel). 2024 Mar 11;13(3):339. doi: 10.3390/antiox13030339 (PMC10968302; doi:10.3390/antiox13030339)
Supplement: Supplementary file 1 [file antioxidants-13-00339-s001.zip › Table S23.pdf]

Table S23. Differences in the serum levels and lipid content of VLDL particles between patients with MS with low and high AOPPs.

	MS			
**Variable**	**Low AOPPs (N=33)**	**High AOPPs (N=32)**	**ALL MS (N=65)**	**p**
VLDL-C	14.5 (10.6, 21.5)	38.6 (31.7, 48.6)	24.2 (14.1, 39.1)	**< 0.0001**
VLDL1-C	5.2 (3.7, 7.3)	15.5 (12.2, 20.1)	9.2 (5.0, 15.9)	**< 0.0001**
VLDL2-C	1.8 (1.2, 2.7)	6.0 (4.7, 8.1)	3.2 (1.7, 6.1)	**< 0.0001**
VLDL3-C	2.2 (1.7, 4.3)	7.5 (5.3, 9.4)	4.6 (2.2, 7.5)	**< 0.0001**
VLDL4-C	4.5 (3.0, 5.5)	8.6 (6.6, 10.7)	6.5 (4.3, 8.8)	**< 0.0001**
VLDL5-C	1.0 (0.8, 1.6)	1.3 (0.8, 1.6)	1.2 (0.8, 1.6)	0.4426
VLDL-FC	6.7 (5.9, 10.2)	15.9 (13.4, 19.4)	10.8 (6.6, 16.0)	**< 0.0001**
VLDL1-FC	1.6 (1.0, 2.5)	5.0 (4.1, 7.3)	3.2 (1.4, 5.0)	**< 0.0001**
VLDL2-FC	0.7 (0.4, 1.1)	2.6 (2.1, 3.8)	1.4 (0.7, 2.6)	**< 0.0001**
VLDL3-FC	0.9 (0.7, 1.8)	3.3 (2.7, 4.2)	2.1 (0.9, 3.3)	**< 0.0001**
VLDL4-FC	1.9 (1.4, 2.9)	4.3 (3.2, 5.0)	3.0 (1.8, 4.3)	**< 0.0001**
VLDL5-FC	0.6 (0.4, 0.9)	0.9 (0.6, 1.2)	0.8 (0.5, 1.0)	0.0022
VLDL-TG	58.8 (43.0, 82.4)	131.7 (116.9, 170.8)	92.3 (58.8, 131.9)	**< 0.0001**
VLDL1-TG	26.2 (20.3, 35.0)	69.9 (50.1, 102.9)	47.5 (26.2, 72.8)	**< 0.0001**
VLDL2-TG	8.2 (5.6, 12.2)	20.7 (18.3, 26.3)	13.3 (8.2, 21.1)	**< 0.0001**
VLDL3-TG	6.6 (4.4, 11.0)	19.1 (15.0, 22.5)	12.2 (6.6, 19.2)	**< 0.0001**
VLDL4-TG	8.0 (5.1, 10.3)	12.8 (10.6, 15.2)	10.3 (8.0, 13.6)	**< 0.0001**
VLDL5-TG	2.8 (2.6, 3.3)	3.2 (2.7, 3.8)	3.0 (2.6, 3.6)	0.1065
VLDL-PL	14.9 (11.8, 21.7)	33.0 (28.4, 37.8)	22.8 (14.3, 33.1)	**< 0.0001**
VLDL1-PL	4.1 (2.9, 5.7)	11.2 (9.3, 16.4)	7.5 (3.9, 11.4)	**< 0.0001**
VLDL2-PL	1.9 (1.5, 3.0)	5.3 (4.7, 6.9)	3.3 (1.9, 5.5)	**< 0.0001**
VLDL3-PL	2.4 (1.7, 4.0)	6.6 (5.1, 7.4)	4.4 (2.4, 6.7)	**< 0.0001**
VLDL4-PL	3.9 (2.9, 5.1)	6.7 (5.6, 8.6)	5.2 (3.9, 7.0)	**< 0.0001**
VLDL5-PL	1.5 (1.1, 2.0)	1.9 (1.6, 2.3)	1.8 (1.2, 2.1)	0.0166
VLDL-apoB	6.6 (5.0, 8.8)	13.3 (11.0, 15.5)	9.2 (6.6, 13.4)	**< 0.0001**
VLDL-C/VLDL-apoB	2.34 (2.06, 2.50)	3.01 (2.66, 3.23)	2.57 (2.28, 3.00)	**< 0.0001**
VLDL-FC/VLDL-apoB	1.14 (1.04, 1.22)	1.22 (1.16, 1.27)	1.19 (1.10, 1.24)	0.0054
VLDL-TG/VLDL-apoB	8.92 (8.10, 10.16)	10.37 (9.65, 11.61)	9.77 (8.63, 11.06)	0.0004
VLDL-PL/VLDL-apoB	2.43 (2.21, 2.56)	2.45 (2.34, 2.62)	2.44 (2.30, 2.60)	0.3315

Data are presented as median (q1, q3). Differences between patients with MS with low and high AOPPs were tested using the Mann-Whitney U test. AOPPs levels below the median (<41.6 μmol/L) were defined as low and those ≥41.6 μmol/L were defined as high AOPPs. Serum levels of lipids and apoB in VLDL are given in mg/dL. *p*-values < 0.0003 are considered statistically significant after a Bonferroni correction for multiple testing and are depicted in bold. AOPPs, advanced oxidation protein products; apoB, apolipoprotein B; C, cholesterol; FC, free cholesterol; HV, healthy volunteer; VLDL, very low-density lipoprotein; MS, metabolic syndrome; PL, phospholipid; TG, triglyceride.
