# Supplementary material for: Advanced Oxidation Protein Products Are Strongly Associated with the Serum Levels and Lipid Contents of Lipoprotein Subclasses in Healthy Volunteers and Patients with Metabolic Syndrome
Source: Antioxidants (Basel). 2024 Mar 11;13(3):339. doi: 10.3390/antiox13030339 (PMC10968302; doi:10.3390/antiox13030339)
Supplement: Supplementary file 1 [file antioxidants-13-00339-s001.zip › Table S24.pdf]

Table S24. Differences in the serum levels and lipid content of IDL particles between HV with low and high AOPPs.

	HV			
**Variable**	**Low AOPPs (N=33)**	**High AOPPs (N=32)**	**ALL HV (N=65)**	**p**
IDL-C	11.2 (8.8, 14.5)	18.7 (15.0, 20.6)	14.5 (10.0, 18.9)	**< 0.0001**
IDL-FC	3.1 (2.4, 4.0)	5.2 (4.3, 6.0)	4.0 (2.9, 5.4)	**< 0.0001**
IDL-TG	4.6 (3.4, 6.3)	10.6 (7.3, 16.4)	7.1 (4.1, 11.6)	**< 0.0001**
IDL-PL	6.3 (5.3, 7.1)	9.2 (7.4, 10.9)	7.3 (6.1, 9.4)	**< 0.0001**
IDL-apoB	4.2 (3.2, 5.2)	6.7 (5.4, 7.3)	5.4 (4.0, 6.9)	**< 0.0001**
IDL-C/IDL-apoB	2.60 (2.44, 2.83)	2.79 (2.64, 2.91)	2.68 (2.49, 2.89)	0.0266
IDL-FC/IDL-apoB	0.73 (0.69, 0.77)	0.79 (0.75, 0.85)	0.76 (0.70, 0.82)	0.0017
IDL-TG/IDL-apoB	1.10 (0.86, 1.44)	1.72 (1.17, 2.45)	1.41 (0.91, 2.06)	0.0004
IDL-PL/IDL-apoB	1.49 (1.31, 1.68)	1.41 (1.28, 1.62)	1.42 (1.31, 1.63)	0.4545

Data are presented as median (q1, q3). Differences between HV with low and high AOPPs were tested using the Mann-Whitney U test. AOPPs levels below the median (<34.6 μmol/L) were defined as low and those ≥34.6 μmol/L were defined as high AOPPs. Serum levels of lipids and apoB in IDL are given in mg/dL. *p*-values < 0.0003 are considered statistically significant after a Bonferroni correction for multiple testing and are depicted in bold. AOPPs, advanced oxidation protein products; apoB, apolipoprotein B; C, cholesterol; FC, free cholesterol; HV, healthy volunteer; IDL, intermediate-density lipoprotein; PL, phospholipid; TG, triglyceride.
