# Supplementary material for: Advanced Oxidation Protein Products Are Strongly Associated with the Serum Levels and Lipid Contents of Lipoprotein Subclasses in Healthy Volunteers and Patients with Metabolic Syndrome
Source: Antioxidants (Basel). 2024 Mar 11;13(3):339. doi: 10.3390/antiox13030339 (PMC10968302; doi:10.3390/antiox13030339)
Supplement: Supplementary file 1 [file antioxidants-13-00339-s001.zip › Table S25.pdf]

Table S25. Differences in the serum levels and lipid content of IDL particles between patients with MS with low and high AOPPs.

	MS			
**Variable**	**Low AOPPs (N=33)**	**High AOPPs (N=32)**	**ALL MS (N=65)**	**p**
IDL-C	13.3 (10.4, 17.5)	26.3 (19.3, 30.3)	18.3 (13.3, 26.6)	**< 0.0001**
IDL-FC	3.8 (2.8, 4.9)	7.6 (5.5, 8.3)	5.3 (3.8, 7.8)	**< 0.0001**
IDL-TG	7.4 (4.5, 11.1)	22.4 (17.2, 29.6)	13.8 (7.3, 22.5)	**< 0.0001**
IDL-PL	6.8 (5.0, 7.6)	12.7 (10.6, 15.2)	9.4 (6.5, 12.7)	**< 0.0001**
IDL-apoB	5.1 (4.6, 6.6)	8.5 (7.0, 9.9)	6.9 (5.1, 9.0)	**< 0.0001**
IDL-C/IDL-apoB	2.48 (2.23, 2.62)	2.93 (2.77, 3.11)	2.75 (2.45, 2.94)	**< 0.0001**
IDL-FC/IDL-apoB	0.69 (0.61, 0.75)	0.84 (0.78, 0.88)	0.76 (0.66, 0.84)	**< 0.0001**
IDL-TG/IDL-apoB	1.26 (0.90, 1.79)	2.62 (2.14, 3.25)	1.97 (1.25, 2.72)	**< 0.0001**
IDL-PL/IDL-apoB	1.14 (1.04, 1.37)	1.46 (1.31, 1.63)	1.35 (1.12, 1.54)	**< 0.0001**

Data are presented as median (q1, q3). Differences between patients with MS with low and high AOPPs were tested using the Mann-Whitney U test. AOPPs levels below the median (<41.6 μmol/L) were defined as low and those ≥41.6 μmol/L were defined as high AOPPs. Serum levels of lipids and apoB in IDL are given in mg/dL. *p*-values < 0.0003 are considered statistically significant after a Bonferroni correction for multiple testing and are depicted in bold. AOPPs, advanced oxidation protein products; apoB, apolipoprotein B; C, cholesterol; FC, free cholesterol; IDL, intermediate-density lipoprotein; MS, metabolic syndrome; PL, phospholipid; TG, triglyceride.
