# Supplementary material for: Advanced Oxidation Protein Products Are Strongly Associated with the Serum Levels and Lipid Contents of Lipoprotein Subclasses in Healthy Volunteers and Patients with Metabolic Syndrome
Source: Antioxidants (Basel). 2024 Mar 11;13(3):339. doi: 10.3390/antiox13030339 (PMC10968302; doi:10.3390/antiox13030339)
Supplement: Supplementary file 1 [file antioxidants-13-00339-s001.zip › Table S26.pdf]

Table S26. Differences in the serum levels of total LDL and LDL subclasses between HV with low and high AOPPs.

	HV			
**Variable (mg/dL)**	**Low AOPPs (N=33)**	**High AOPPs (N=32)**	**ALL HV (N=65)**	**p**
LDL-C	132.8 (120.1, 153.1)	142.9 (123.5, 157.7)	137.4 (121.8, 154.3)	0.1851
LDL1-C	31.5 (27.6, 36.7)	29.8 (25.3, 36.2)	30.4 (26.5, 36.7)	0.5461
LDL2-C	26.7 (25.7, 32.0)	24.3 (16.2, 28.3)	25.9 (20.1, 30.7)	0.0169
LDL3-C	24.4 (21.7, 29.1)	23.0 (19.5, 29.0)	23.8 (21.0, 29.1)	0.3722
LDL4-C	17.4 (14.6, 22.3)	22.0 (18.4, 26.1)	20.3 (15.8, 24.4)	0.0369
LDL5-C	13.4 (10.8, 17.9)	18.3 (15.7, 25.2)	15.9 (12.4, 21.9)	0.0007
LDL6-C	17.3 (16.4, 20.2)	21.5 (17.7, 27.4)	19.2 (16.5, 22.8)	0.0057
LDL-FC	41.7 (38.0, 47.0)	42.7 (38.0, 49.7)	42.2 (38.0, 47.3)	0.5505
LDL1-FC	9.9 (8.8, 11.3)	9.3 (8.1, 11.1)	9.5 (8.4, 11.3)	0.5861
LDL2-FC	8.9 (8.5, 10.2)	7.7 (5.4, 9.1)	8.6 (6.7, 9.8)	0.0047
LDL3-FC	8.3 (7.4, 9.6)	7.6 (6.1, 8.8)	7.9 (7.1, 9.2)	0.0588
LDL4-FC	6.3 (5.2, 7.2)	6.7 (5.7, 7.8)	6.6 (5.3, 7.6)	0.3155
LDL5-FC	4.6 (3.9, 5.9)	6.0 (4.9, 7.1)	5.3 (4.2, 6.5)	0.0079
LDL6-FC	5.5 (5.2, 6.2)	6.2 (4.9, 7.2)	5.8 (4.9, 6.5)	0.0754
LDL-TG	17.2 (15.1, 20.0)	22.2 (20.3, 24.7)	20.0 (16.5, 23.6)	0.0004
LDL1-TG	5.7 (4.7, 6.8)	6.8 (6.0, 8.3)	6.4 (5.1, 7.6)	0.0056
LDL2-TG	2.7 (2.1, 2.9)	2.6 (2.3, 3.3)	2.7 (2.1, 3.2)	0.5245
LDL3-TG	2.5 (2.1, 2.8)	2.6 (2.0, 3.0)	2.5 (2.1, 3.0)	0.9372
LDL4-TG	1.9 (1.4, 2.5)	2.8 (2.5, 3.2)	2.5 (1.6, 3.0)	0.0010
LDL5-TG	1.6 (1.0, 2.0)	2.8 (2.1, 3.5)	2.0 (1.4, 3.0)	**< 0.0001**
LDL6-TG	2.7 (2.1, 3.0)	3.1 (2.5, 4.0)	2.8 (2.3, 3.4)	0.0166
LDL-PL	74.4 (66.9, 83.1)	79.6 (70.6, 86.7)	75.9 (67.9, 85.4)	0.2078
LDL1-PL	17.3 (14.7, 19.9)	16.4 (13.9, 19.0)	16.6 (14.7, 19.7)	0.5549
LDL2-PL	14.8 (13.7, 16.9)	13.2 (8.8, 15.0)	14.0 (11.1, 16.2)	0.0192
LDL3-PL	13.4 (11.9, 15.6)	12.2 (10.6, 15.3)	13.2 (11.3, 15.6)	0.3061
LDL4-PL	9.3 (8.1, 12.0)	12.0 (10.2, 14.0)	11.4 (9.0, 13.1)	0.0304
LDL5-PL	7.5 (6.2, 9.6)	9.8 (8.5, 13.4)	8.7 (7.0, 11.8)	0.0007
LDL6-PL	10.5 (9.5, 11.8)	12.1 (10.4, 14.9)	11.2 (10.0, 12.8)	0.0199
LDL-apoB	76.6 (70.2, 87.8)	89.7 (80.8, 98.4)	82.3 (72.8, 94.9)	0.0122
LDL1-apoB	15.7 (13.6, 18.5)	15.0 (13.1, 17.9)	15.3 (13.4, 18.4)	0.6508
LDL2-apoB	14.9 (13.3, 16.7)	13.5 (9.2, 15.0)	13.8 (11.2, 16.4)	0.0672
LDL3-apoB	13.8 (12.4, 16.3)	13.7 (11.4, 16.5)	13.7 (12.0, 16.4)	0.6366
LDL4-apoB	10.5 (9.2, 14.1)	14.2 (11.7, 16.9)	13.4 (9.7, 15.3)	0.0085
LDL5-apoB	9.1 (7.4, 12.2)	13.0 (10.5, 18.0)	11.3 (8.4, 15.2)	**0.0002**
LDL6-apoB	14.0 (13.2, 16.4)	17.8 (14.9, 22.6)	15.8 (13.4, 18.0)	0.0014

Data are presented as median (q1, q3). Differences between HV with low and high AOPPs were tested using the Mann-Whitney U test. AOPPs levels below the median (<34.6 μmol/L) were defined as low and those ≥34.6 μmol/L were defined as high AOPPs. *p*-values < 0.0003 are considered statistically significant after a Bonferroni correction for multiple testing and are depicted in bold. AOPPs, advanced oxidation protein products; apoB, apolipoprotein B; C, cholesterol; FC, free cholesterol; HV, healthy volunteer; LDL, low-density lipoprotein; PL, phospholipid; TG, triglyceride.
