# Supplementary material for: Advanced Oxidation Protein Products Are Strongly Associated with the Serum Levels and Lipid Contents of Lipoprotein Subclasses in Healthy Volunteers and Patients with Metabolic Syndrome
Source: Antioxidants (Basel). 2024 Mar 11;13(3):339. doi: 10.3390/antiox13030339 (PMC10968302; doi:10.3390/antiox13030339)
Supplement: Supplementary file 1 [file antioxidants-13-00339-s001.zip › Table S27.pdf]

Table S27. Differences in the serum levels of total LDL and LDL subclasses between patients with MS with low and high AOPPs.

	MS			
**Variable (mg/dL)**	**Low AOPPs (N=33)**	**High AOPPs (N=32)**	**ALL MS (N=65)**	**p**
LDL-C	118.7 (90.4, 131.6)	116.6 (89.8, 152.3)	117.1 (90.4, 148.1)	0.5203
LDL1-C	24.4 (21.5, 29.9)	25.1 (21.0, 31.2)	24.5 (21.3, 30.1)	0.7930
LDL2-C	19.7 (12.6, 23.0)	15.2 (10.4, 18.6)	17.4 (12.2, 22.3)	0.0182
LDL3-C	18.9 (12.1, 22.2)	15.1 (8.8, 21.0)	17.6 (9.9, 22.2)	0.0787
LDL4-C	17.6 (12.9, 21.2)	18.1 (9.7, 24.3)	17.9 (11.2, 24.1)	0.8235
LDL5-C	14.5 (12.9, 18.2)	20.9 (15.4, 26.0)	17.4 (13.1, 23.8)	0.0136
LDL6-C	17.6 (14.3, 20.6)	28.2 (23.0, 35.9)	22.1 (17.1, 28.8)	**< 0.0001**
LDL-FC	37.4 (29.1, 40.6)	36.6 (28.3, 45.4)	37.3 (28.8, 45.1)	0.9477
LDL1-FC	7.8 (7.3, 9.5)	8.5 (7.0, 10.5)	8.2 (7.1, 9.8)	0.5076
LDL2-FC	6.8 (5.6, 8.2)	5.6 (4.6, 6.9)	6.4 (4.9, 7.7)	0.0309
LDL3-FC	6.8 (4.9, 7.3)	5.5 (3.9, 7.3)	6.5 (4.3, 7.3)	0.0662
LDL4-FC	5.8 (4.8, 6.9)	5.8 (3.9, 7.4)	5.8 (4.5, 7.1)	0.5461
LDL5-FC	5.0 (4.5, 6.0)	6.1 (5.0, 7.7)	5.3 (4.6, 6.8)	0.0615
LDL6-FC	5.6 (4.8, 6.4)	7.6 (6.4, 9.6)	6.4 (5.3, 7.6)	**< 0.0001**
LDL-TG	20.9 (18.1, 23.8)	27.9 (23.9, 31.8)	23.8 (20.5, 28.3)	**< 0.0001**
LDL1-TG	6.5 (5.7, 8.2)	8.1 (7.1, 10.1)	7.5 (6.3, 8.6)	0.0007
LDL2-TG	2.5 (2.1, 3.2)	2.6 (2.2, 3.2)	2.6 (2.1, 3.2)	0.3617
LDL3-TG	2.5 (2.0, 3.0)	2.3 (1.8, 2.9)	2.4 (1.9, 3.0)	0.6650
LDL4-TG	2.7 (2.0, 3.1)	3.5 (2.6, 4.6)	2.9 (2.1, 3.6)	0.0124
LDL5-TG	2.3 (1.9, 2.7)	4.1 (3.3, 5.0)	3.1 (2.2, 4.3)	**< 0.0001**
LDL6-TG	3.4 (2.6, 3.9)	4.7 (3.9, 5.7)	3.9 (2.9, 4.7)	**< 0.0001**
LDL-PL	66.6 (52.3, 74.9)	65.7 (49.6, 83.5)	66.6 (51.5, 80.2)	0.7528
LDL1-PL	14.1 (12.5, 16.9)	14.3 (12.1, 17.5)	14.3 (12.1, 16.9)	0.8956
LDL2-PL	11.0 (7.8, 12.7)	8.4 (6.4, 10.1)	9.6 (7.0, 12.4)	0.0089
LDL3-PL	10.8 (7.3, 12.5)	8.8 (5.8, 11.5)	10.1 (6.3, 12.4)	0.0643
LDL4-PL	10.0 (7.7, 11.4)	10.3 (6.0, 13.3)	10.2 (6.7, 12.9)	0.8956
LDL5-PL	8.3 (7.2, 10.2)	11.4 (8.0, 13.9)	9.6 (7.5, 12.6)	0.0160
LDL6-PL	10.6 (8.8, 12.6)	15.2 (13.1, 18.6)	12.6 (10.2, 15.3)	**< 0.0001**
LDL-apoB	73.4 (61.2, 83.6)	88.4 (71.4, 107.8)	78.5 (65.0, 92.1)	0.0163
LDL1-apoB	13.8 (11.2, 16.1)	14.1 (11.7, 16.7)	13.9 (11.5, 16.3)	0.5997
LDL2-apoB	11.1 (7.7, 13.0)	9.2 (6.9, 11.2)	9.9 (7.3, 12.8)	0.0692
LDL3-apoB	11.5 (8.2, 13.2)	10.1 (6.3, 13.4)	10.8 (6.9, 13.2)	0.1545
LDL4-apoB	11.7 (9.3, 13.3)	13.5 (7.2, 16.9)	12.3 (8.4, 16.2)	0.5771
LDL5-apoB	10.5 (9.7, 12.8)	15.6 (12.0, 19.4)	12.8 (9.9, 17.7)	0.0011
LDL6-apoB	15.2 (11.8, 16.2)	23.7 (19.8, 30.7)	18.0 (14.1, 23.9)	**< 0.0001**

Data are presented as median (q1, q3). Differences between patients with MS with low and high AOPPs were tested using the Mann-Whitney U test. AOPPs levels below the median (<41.6 μmol/L) were defined as low and those ≥41.6 μmol/L were defined as high AOPPs. *p*-values < 0.0003 are considered statistically significant after a Bonferroni correction for multiple testing and are depicted in bold. AOPPs, advanced oxidation protein products; apoB, apolipoprotein B; C, cholesterol; FC, free cholesterol; LDL, low-density lipoprotein; MS, metabolic syndrome patient; PL, phospholipid; TG, triglyceride.
