# Supplementary material for: Advanced Oxidation Protein Products Are Strongly Associated with the Serum Levels and Lipid Contents of Lipoprotein Subclasses in Healthy Volunteers and Patients with Metabolic Syndrome
Source: Antioxidants (Basel). 2024 Mar 11;13(3):339. doi: 10.3390/antiox13030339 (PMC10968302; doi:10.3390/antiox13030339)
Supplement: Supplementary file 1 [file antioxidants-13-00339-s001.zip › Table S28.pdf]

Table S28. Differences in the lipid content of LDL between HV with low and high AOPPs.

	HV			
	**Low AOPPs (N=33)**	**High AOPPs (N=32)**	**ALL HV (N=65)**	**p**
LDL-C/LDL-apoB	1.71 (1.67, 1.75)	1.62 (1.52, 1.68)	1.68 (1.58, 1.73)	**< 0.0001**
LDL1-C/LDL1-apoB	1.96 (1.94, 1.99)	1.95 (1.89, 1.99)	1.96 (1.91, 1.99)	0.1851
LDL2-C/LDL2-apoB	1.88 (1.84, 1.91)	1.80 (1.73, 1.84)	1.84 (1.78, 1.88)	**< 0.0001**
LDL3-C/LDL3-apoB	1.77 (1.73, 1.80)	1.72 (1.66, 1.76)	1.74 (1.69, 1.78)	0.0015
LDL4-C/LDL4-apoB	1.62 (1.57, 1.67)	1.55 (1.47, 1.59)	1.58 (1.53, 1.64)	0.0016
LDL5-C/LDL5-apoB	1.44 (1.40, 1.49)	1.41 (1.38, 1.46)	1.43 (1.38, 1.48)	0.0796
LDL6-C/LDL6-apoB	1.24 (1.20, 1.28)	1.21 (1.16, 1.25)	1.23 (1.19, 1.27)	0.0461
LDL-FC/LDL-apoB	0.53 (0.52, 0.55)	0.49 (0.45, 0.51)	0.51 (0.49, 0.53)	**< 0.0001**
LDL1-FC/LDL1-apoB	0.62 (0.61, 0.63)	0.61 (0.59, 0.62)	0.61 (0.59, 0.63)	0.1065
LDL2-FC/LDL2-apoB	0.63 (0.61, 0.65)	0.59 (0.57, 0.63)	0.62 (0.58, 0.64)	0.0046
LDL3-FC/LDL3-apoB	0.60 (0.56, 0.63)	0.54 (0.52, 0.59)	0.57 (0.53, 0.61)	0.0003
LDL4-FC/LDL4-apoB	0.54 (0.51, 0.61)	0.47 (0.44, 0.50)	0.51 (0.46, 0.55)	**< 0.0001**
LDL5-FC/LDL5-apoB	0.51 (0.46, 0.56)	0.42 (0.40, 0.45)	0.46 (0.42, 0.52)	**< 0.0001**
LDL6-FC/LDL6-apoB	0.39 (0.38, 0.41)	0.34 (0.32, 0.37)	0.38 (0.34, 0.40)	**< 0.0001**
LDL-TG/LDL-apoB	0.23 (0.20, 0.26)	0.25 (0.22, 0.28)	0.24 (0.21, 0.27)	0.0394
LDL1-TG/LDL1-apoB	0.36 (0.33, 0.38)	0.43 (0.37, 0.48)	0.38 (0.35, 0.45)	**< 0.0001**
LDL2-TG/LDL2-apoB	0.18 (0.16, 0.21)	0.22 (0.18, 0.26)	0.19 (0.16, 0.23)	0.0010
LDL3-TG/LDL3-apoB	0.17 (0.15, 0.21)	0.18 (0.16, 0.20)	0.17 (0.15, 0.21)	0.6650
LDL4-TG/LDL4-apoB	0.16 (0.14, 0.21)	0.20 (0.16, 0.23)	0.18 (0.15, 0.21)	0.0382
LDL5-TG/LDL5-apoB	0.16 (0.14, 0.21)	0.19 (0.16, 0.23)	0.18 (0.15, 0.22)	0.0918
LDL6-TG/LDL6-apoB	0.18 (0.17, 0.20)	0.17 (0.16, 0.19)	0.18 (0.16, 0.20)	0.2376
LDL-PL/LDL-apoB	0.95 (0.93, 0.96)	0.89 (0.84, 0.91)	0.92 (0.88, 0.95)	**< 0.0001**
LDL1-PL/LDL1-apoB	1.07 (1.07, 1.09)	1.06 (1.04, 1.08)	1.07 (1.05, 1.09)	0.0039
LDL2-PL/LDL2-apoB	1.01 (1.00, 1.04)	0.98 (0.96, 1.00)	1.00 (0.98, 1.02)	**< 0.0001**
LDL3-PL/LDL3-apoB	0.96 (0.95, 0.98)	0.93 (0.92, 0.95)	0.95 (0.93, 0.96)	**< 0.0001**
LDL4-PL/LDL4-apoB	0.88 (0.87, 0.91)	0.85 (0.82, 0.86)	0.86 (0.84, 0.89)	**< 0.0001**
LDL5-PL/LDL5-apoB	0.80 (0.78, 0.83)	0.76 (0.75, 0.77)	0.78 (0.76, 0.81)	**< 0.0001**
LDL6-PL/LDL6-apoB	0.75 (0.71, 0.79)	0.68 (0.66, 0.71)	0.71 (0.68, 0.76)	**< 0.0001**

Data are presented as median (q1, q3). Differences between HV with low and high AOPPs were tested using the Mann-Whitney U test. AOPPs levels below the median (<34.6 μmol/L) were defined as low and those ≥34.6 μmol/L were defined as high AOPPs. *p*-values < 0.0003 are considered statistically significant after a Bonferroni correction for multiple testing and are depicted in bold. AOPPs, advanced oxidation protein products; apoB, apolipoprotein B; C, cholesterol; FC, free cholesterol; HV, healthy volunteer; LDL, low-density lipoprotein; PL, phospholipid; TG, triglyceride.
