# Supplementary material for: Advanced Oxidation Protein Products Are Strongly Associated with the Serum Levels and Lipid Contents of Lipoprotein Subclasses in Healthy Volunteers and Patients with Metabolic Syndrome
Source: Antioxidants (Basel). 2024 Mar 11;13(3):339. doi: 10.3390/antiox13030339 (PMC10968302; doi:10.3390/antiox13030339)
Supplement: Supplementary file 1 [file antioxidants-13-00339-s001.zip › Table S29.pdf]

Table S29. Differences in the lipid content of LDL between patients with MS with low and high AOPPs.

	MS			
**Variable**	**Low AOPPs (N=33)**	**High AOPPs (N=32)**	**ALL MS (N=65)**	**p**
LDL-C/LDL-apoB	1.58 (1.55, 1.64)	1.45 (1.29, 1.52)	1.54 (1.43, 1.61)	**< 0.0001**
LDL1-C/LDL1-apoB	1.80 (1.73, 1.89)	1.81 (1.65, 1.87)	1.80 (1.72, 1.89)	0.6555
LDL2-C/LDL2-apoB	1.75 (1.68, 1.81)	1.63 (1.54, 1.74)	1.71 (1.60, 1.78)	0.0034
LDL3-C/LDL3-apoB	1.61 (1.57, 1.71)	1.49 (1.32, 1.61)	1.58 (1.46, 1.68)	0.0012
LDL4-C/LDL4-apoB	1.50 (1.41, 1.56)	1.39 (1.23, 1.45)	1.44 (1.35, 1.53)	0.0007
LDL5-C/LDL5-apoB	1.39 (1.31, 1.43)	1.33 (1.25, 1.38)	1.36 (1.27, 1.41)	0.0097
LDL6-C/LDL6-apoB	1.21 (1.15, 1.26)	1.15 (1.10, 1.18)	1.18 (1.13, 1.22)	0.0018
LDL-FC/LDL-apoB	0.50 (0.49, 0.54)	0.45 (0.40, 0.47)	0.48 (0.44, 0.50)	**< 0.0001**
LDL1-FC/LDL1-apoB	0.60 (0.58, 0.62)	0.60 (0.56, 0.63)	0.60 (0.57, 0.63)	0.6746
LDL2-FC/LDL2-apoB	0.64 (0.59, 0.69)	0.62 (0.58, 0.66)	0.63 (0.59, 0.68)	0.3448
LDL3-FC/LDL3-apoB	0.59 (0.53, 0.64)	0.55 (0.50, 0.59)	0.57 (0.52, 0.63)	0.1280
LDL4-FC/LDL4-apoB	0.51 (0.47, 0.54)	0.45 (0.41, 0.49)	0.49 (0.45, 0.53)	0.0033
LDL5-FC/LDL5-apoB	0.46 (0.44, 0.49)	0.40 (0.35, 0.41)	0.42 (0.39, 0.46)	**< 0.0001**
LDL6-FC/LDL6-apoB	0.38 (0.35, 0.41)	0.32 (0.29, 0.34)	0.34 (0.31, 0.38)	**< 0.0001**
LDL-TG/LDL-apoB	0.29 (0.25, 0.34)	0.33 (0.29, 0.40)	0.31 (0.27, 0.37)	0.0175
LDL1-TG/LDL1-apoB	0.48 (0.40, 0.58)	0.60 (0.54, 0.76)	0.55 (0.43, 0.64)	0.0010
LDL2-TG/LDL2-apoB	0.23 (0.21, 0.26)	0.30 (0.24, 0.38)	0.25 (0.21, 0.32)	0.0033
LDL3-TG/LDL3-apoB	0.21 (0.19, 0.26)	0.25 (0.20, 0.36)	0.24 (0.19, 0.28)	0.0662
LDL4-TG/LDL4-apoB	0.22 (0.18, 0.27)	0.27 (0.23, 0.36)	0.25 (0.20, 0.31)	0.0144
LDL5-TG/LDL5-apoB	0.20 (0.18, 0.22)	0.24 (0.21, 0.30)	0.22 (0.19, 0.26)	0.0004
LDL6-TG/LDL6-apoB	0.22 (0.19, 0.24)	0.19 (0.17, 0.22)	0.20 (0.18, 0.24)	0.0722
LDL-PL/LDL-apoB	0.91 (0.87, 0.94)	0.81 (0.74, 0.85)	0.87 (0.81, 0.91)	**< 0.0001**
LDL1-PL/LDL1-apoB	1.06 (1.03, 1.07)	1.03 (0.98, 1.05)	1.04 (1.02, 1.07)	0.0113
LDL2-PL/LDL2-apoB	0.99 (0.96, 1.02)	0.94 (0.89, 0.96)	0.96 (0.92, 1.00)	**< 0.0001**
LDL3-PL/LDL3-apoB	0.93 (0.91, 0.96)	0.88 (0.78, 0.91)	0.91 (0.87, 0.94)	**< 0.0001**
LDL4-PL/LDL4-apoB	0.84 (0.82, 0.86)	0.78 (0.75, 0.80)	0.82 (0.77, 0.85)	**< 0.0001**
LDL5-PL/LDL5-apoB	0.78 (0.75, 0.79)	0.72 (0.69, 0.74)	0.74 (0.71, 0.78)	**< 0.0001**
LDL6-PL/LDL6-apoB	0.73 (0.68, 0.77)	0.64 (0.61, 0.66)	0.67 (0.64, 0.74)	**< 0.0001**

Data are presented as median (q1, q3). Differences between patients with MS with low and high AOPPs were tested using the Mann-Whitney U test. AOPPs levels below the median (<41.6 μmol/L) were defined as low and those ≥41.6 μmol/L were defined as high AOPPs. *p*-values < 0.0003 are considered statistically significant after a Bonferroni correction for multiple testing and are depicted in bold. AOPPs, advanced oxidation protein products; apoB, apolipoprotein B; C, cholesterol; FC, free cholesterol; LDL, low-density lipoprotein; MS, metabolic syndrome; PL, phospholipid; TG, triglyceride.
