# Supplementary material for: Advanced Oxidation Protein Products Are Strongly Associated with the Serum Levels and Lipid Contents of Lipoprotein Subclasses in Healthy Volunteers and Patients with Metabolic Syndrome
Source: Antioxidants (Basel). 2024 Mar 11;13(3):339. doi: 10.3390/antiox13030339 (PMC10968302; doi:10.3390/antiox13030339)
Supplement: Supplementary file 1 [file antioxidants-13-00339-s001.zip › Table S3.pdf]

Table S3. Differences in serum levels and lipid content of VLDL particles between HV and patients with MS.

Variable	All (N=130)	HV (N=65)	MS (N=65)	p
VLDL-C	20.5 (12.1, 32.2)	16.2 (11.2, 24.1)	24.2 (14.1, 39.1)	0.0005
VLDL1-C	6.3 (4.0, 12.3)	5.4 (3.1, 8.1)	9.2 (5.0, 15.9)	< **0.0001**
VLDL2-C	2.7 (1.6, 4.8)	2.3 (1.4, 3.5)	3.2 (1.7, 6.1)	0.0151
VLDL3-C	3.7 (2.0, 5.6)	2.8 (1.9, 4.5)	4.6 (2.2, 7.5)	0.0015
VLDL4-C	5.2 (3.7, 7.4)	4.5 (3.4, 6.1)	6.5 (4.3, 8.8)	0.0008
VLDL5-C	1.2 (0.8, 1.6)	1.1 (0.8, 1.5)	1.2 (0.8, 1.6)	0.9166
VLDL-FC	9.0 (6.1, 13.4)	7.6 (5.6, 10.6)	10.8 (6.6, 16.0)	0.0003
VLDL1-FC	2.3 (1.1, 4.1)	1.8 (0.9, 2.9)	3.2 (1.4, 5.0)	**0.0002**
VLDL2-FC	1.0 (0.5, 2.2)	0.9 (0.4, 1.5)	1.4 (0.7, 2.6)	0.0017
VLDL3-FC	1.5 (0.8, 2.6)	1.1 (0.7, 2.0)	2.1 (0.9, 3.3)	0.0003
VLDL4-FC	2.4 (1.5, 3.6)	2.0 (1.3, 2.7)	3.0 (1.8, 4.3)	0.0003
VLDL5-FC	0.6 (0.4, 1.0)	0.6 (0.4, 0.9)	0.8 (0.5, 1.0)	0.0533
VLDL-TG	69.3 (43.8, 116.4)	54.5 (39.0, 80.3)	92.3 (58.8, 131.9)	< **0.0001**
VLDL1-TG	29.2 (18.7, 55.4)	25.1 (13.8, 39.4)	47.5 (26.2, 72.8)	< **0.0001**
VLDL2-TG	10.3 (6.1, 18.1)	8.9 (5.0, 11.6)	13.3 (8.2, 21.1)	< **0.0001**
VLDL3-TG	9.7 (5.6, 14.9)	7.6 (4.8, 11.9)	12.2 (6.6, 19.2)	0.0003
VLDL4-TG	8.8 (5.9, 11.6)	6.8 (5.2, 9.2)	10.3 (8.0, 13.6)	< **0.0001**
VLDL5-TG	2.7 (2.4, 3.3)	2.6 (2.2, 2.9)	3.0 (2.6, 3.6)	**0.0001**
VLDL-PL	18.8 (12.4, 28.4)	15.6 (10.7, 21.5)	22.8 (14.3, 33.1)	< **0.0001**
VLDL1-PL	4.8 (2.9, 9.3)	4.0 (2.3, 6.5)	7.5 (3.9, 11.4)	< **0.0001**
VLDL2-PL	2.7 (1.6, 4.7)	2.2 (1.3, 3.1)	3.3 (1.9, 5.5)	0.0003
VLDL3-PL	3.5 (1.9, 5.2)	2.7 (1.7, 4.3)	4.4 (2.4, 6.7)	0.0003
VLDL4-PL	4.5 (3.1, 6.0)	3.8 (2.8, 4.9)	5.2 (3.9, 7.0)	< **0.0001**
VLDL5-PL	1.6 (1.2, 2.0)	1.5 (1.1, 2.0)	1.8 (1.2, 2.1)	0.0539
VLDL-apoB	7.9 (5.3, 11.0)	6.2 (4.1, 8.8)	9.2 (6.6, 13.4)	< **0.0001**
VLDL-C/VLDL-apoB	2.63 (2.31, 2.91)	2.64 (2.38, 2.84)	2.57 (2.28, 3.00)	0.7961
VLDL-FC/VLDL-apoB	1.22 (1.14, 1.29)	1.24 (1.17, 1.34)	1.19 (1.10, 1.24)	0.0004
VLDL-TG/VLDL-apoB	9.39 (8.60, 10.60)	9.14 (8.60, 10.16)	9.77 (8.63, 11.06)	0.1161
VLDL-PL/VLDL-apoB	2.50 (2.34, 2.62)	2.52 (2.42, 2.63)	2.44 (2.30, 2.60)	0.0160

Data are presented as median (q1, q3). Differences between HV and patients with MS were tested using the Mann-Whitney U test. Serum levels of lipids and apoB in VLDL are given in mg/dL. *p*-values < 0.0003 are considered statistically significant and are depicted in bold. ApoB, apolipoprotein B; C, cholesterol; FC, free cholesterol; HV, healthy volunteer; VLDL, very low-density lipoprotein; MS, metabolic syndrome patient; PL, phospholipid; TG, triglyceride.
