# Supplementary material for: Advanced Oxidation Protein Products Are Strongly Associated with the Serum Levels and Lipid Contents of Lipoprotein Subclasses in Healthy Volunteers and Patients with Metabolic Syndrome
Source: Antioxidants (Basel). 2024 Mar 11;13(3):339. doi: 10.3390/antiox13030339 (PMC10968302; doi:10.3390/antiox13030339)
Supplement: Supplementary file 1 [file antioxidants-13-00339-s001.zip › Table S30.pdf]

Table S30. Differences in the serum levels of total HDL and HDL subclasses between HV with low and high AOPPs.

	HV			
**Variable (mg/dL)**	**Low AOPPs (N=33)**	**High AOPPs (N=32)**	**ALL HV (N=65)**	**p**
HDL-C	74.1 (66.2, 82.4)	58.9 (52.7, 63.6)	65.2 (57.7, 74.5)	**< 0.0001**
HDL1-C	26.5 (18.8, 31.7)	16.6 (13.4, 18.4)	18.4 (15.1, 26.8)	**< 0.0001**
HDL2-C	11.8 (9.5, 13.3)	8.5 (7.4, 9.6)	9.5 (8.3, 12.6)	**< 0.0001**
HDL3-C	13.4 (11.9, 14.3)	11.2 (10.4, 12.3)	12.2 (10.7, 13.6)	**< 0.0001**
HDL4-C	22.6 (20.5, 24.0)	20.7 (17.7, 24.8)	22.3 (18.2, 24.7)	0.5637
HDL-FC	18.3 (16.1, 21.0)	14.3 (12.5, 15.6)	16.0 (14.1, 18.9)	**< 0.0001**
HDL1-FC	7.4 (5.3, 8.3)	5.0 (4.0, 5.5)	5.4 (4.8, 7.4)	**< 0.0001**
HDL2-FC	3.1 (2.7, 3.4)	2.4 (2.0, 2.7)	2.7 (2.4, 3.1)	**< 0.0001**
HDL3-FC	3.1 (2.8, 3.5)	2.8 (2.4, 3.2)	3.0 (2.6, 3.4)	0.0127
HDL4-FC	4.9 (4.2, 5.7)	4.9 (3.9, 5.7)	4.9 (4.0, 5.7)	0.6745
HDL-TG	9.7 (7.6, 11.5)	10.1 (9.2, 11.9)	9.9 (8.7, 11.8)	0.1564
HDL1-TG	3.5 (2.6, 4.7)	2.9 (2.5, 3.8)	3.0 (2.5, 4.2)	0.2008
HDL2-TG	1.6 (1.2, 2.1)	1.7 (1.4, 2.2)	1.6 (1.3, 2.1)	0.2375
HDL3-TG	1.9 (1.6, 2.3)	2.2 (2.1, 2.8)	2.1 (1.7, 2.5)	0.0049
HDL4-TG	2.8 (2.2, 3.6)	3.8 (3.4, 4.3)	3.4 (2.5, 3.9)	**< 0.0001**
HDL-PL	99.4 (89.7, 107.8)	80.6 (74.0, 89.1)	89.4 (79.2, 99.8)	**< 0.0001**
HDL1-PL	33.0 (21.6, 38.2)	19.1 (16.6, 22.2)	22.1 (18.6, 33.9)	**< 0.0001**
HDL2-PL	17.0 (14.2, 19.5)	13.1 (11.8, 14.5)	14.4 (12.6, 18.6)	**0.0001**
HDL3-PL	20.3 (18.3, 21.9)	18.1 (16.5, 19.6)	19.1 (17.4, 20.9)	0.0030
HDL4-PL	30.0 (28.0, 32.7)	29.3 (26.3, 32.4)	29.9 (26.4, 32.7)	0.6413
HDL-apoA-I	181.8 (167.8, 193.1)	159.2 (145.0, 168.5)	167.8 (155.7, 183.7)	**< 0.0001**
HDL1-apoA-I	44.3 (28.3, 52.7)	24.1 (19.7, 27.4)	27.5 (22.3, 45.4)	**< 0.0001**
HDL2-apoA-I	22.8 (19.7, 25.5)	18.0 (16.2, 19.4)	19.6 (17.2, 23.3)	**< 0.0001**
HDL3-apoA-I	33.0 (29.5, 35.6)	29.2 (27.1, 31.5)	30.8 (27.5, 33.5)	0.0018
HDL4-apoA-I	81.9 (77.1, 88.3)	82.0 (71.8, 92.6)	81.9 (72.7, 91.1)	0.8133
HDL-apoA-II	36.2 (33.9, 38.8)	35.3 (32.7, 38.0)	36.0 (33.6, 38.6)	0.2482
HDL1-apoA-II	3.7 (2.5, 4.7)	2.3 (1.8, 2.5)	2.5 (2.1, 4.1)	**0.0002**
HDL2-apoA-II	4.3 (3.6, 4.7)	3.9 (3.2, 4.1)	4.0 (3.5, 4.6)	0.0341
HDL3-apoA-II	7.3 (6.9, 8.1)	7.4 (6.9, 8.1)	7.3 (6.9, 8.1)	0.9948
HDL4-apoA-II	20.3 (18.4, 22.5)	21.5 (18.3, 23.9)	20.9 (18.4, 23.2)	0.3315

Data are presented as median (q1, q3). Differences between HV with low and high AOPPs were tested using the Mann-Whitney U test. AOPPs levels below the median (<34.6 µmol/L) were defined as low and those equal to or higher than ≥34.6 µmol/L were defined as high AOPPs. Serum levels of lipids and apolipoproteins in HDL are given in mg/dL. *p*-values < 0.0003 are considered statistically significant after a Bonferroni correction for multiple testing and are depicted in bold. AOPPs, advanced oxidation protein products; apoA-I, apolipoprotein A-I; apoA-II, apolipoprotein A-II; C, cholesterol; FC, free cholesterol; HDL, high-density lipoprotein; HV, healthy volunteer; PL, phospholipid; TG, triglyceride.
