# Supplementary material for: Advanced Oxidation Protein Products Are Strongly Associated with the Serum Levels and Lipid Contents of Lipoprotein Subclasses in Healthy Volunteers and Patients with Metabolic Syndrome
Source: Antioxidants (Basel). 2024 Mar 11;13(3):339. doi: 10.3390/antiox13030339 (PMC10968302; doi:10.3390/antiox13030339)
Supplement: Supplementary file 1 [file antioxidants-13-00339-s001.zip › Table S31.pdf]

Table S31. Differences in the serum levels of total HDL and HDL subclasses between patients with MS with low and high AOPPs.

	MS			
**Variable (mg/dL)**	**Low AOPPs (N=33)**	**High AOPPs (N=32)**	**ALL MS (N=65)**	**p**
HDL-C	58.6 (52.7, 67.8)	48.0 (43.7, 52.8)	52.7 (47.9, 60.6)	**< 0.0001**
HDL1-C	16.8 (13.1, 20.2)	14.9 (11.7, 18.0)	15.7 (12.9, 20.1)	0.1434
HDL2-C	8.7 (7.4, 10.2)	7.7 (6.5, 8.6)	8.2 (7.3, 9.7)	0.0124
HDL3-C	11.4 (10.0, 13.3)	9.8 (9.3, 10.6)	10.4 (9.6, 11.9)	0.0012
HDL4-C	20.0 (18.2, 23.9)	16.3 (12.5, 19.9)	19.0 (16.0, 22.7)	**0.0002**
HDL-FC	14.6 (12.5, 16.5)	13.0 (11.3, 13.8)	13.5 (11.8, 15.0)	0.0012
HDL1-FC	4.6 (4.2, 5.8)	4.1 (3.6, 4.7)	4.3 (3.7, 5.2)	0.0447
HDL2-FC	2.6 (2.1, 3.0)	2.2 (2.0, 2.4)	2.3 (2.0, 2.7)	0.0220
HDL3-FC	2.7 (2.3, 3.4)	2.3 (1.9, 2.8)	2.5 (2.1, 3.0)	0.0198
HDL4-FC	4.7 (4.0, 5.3)	4.0 (2.9, 4.7)	4.4 (3.8, 5.2)	0.0077
HDL-TG	10.3 (8.6, 11.6)	13.1 (11.2, 17.0)	11.4 (9.7, 13.6)	0.0004
HDL1-TG	3.0 (2.1, 3.9)	3.7 (3.0, 6.5)	3.6 (2.7, 4.6)	0.0136
HDL2-TG	1.8 (1.5, 2.1)	2.3 (2.0, 3.2)	2.0 (1.6, 2.5)	0.0008
HDL3-TG	2.2 (1.9, 2.7)	2.9 (2.6, 3.9)	2.7 (2.1, 3.1)	**0.0001**
HDL4-TG	3.5 (3.0, 3.7)	4.6 (3.9, 5.0)	3.7 (3.3, 4.7)	**< 0.0001**
HDL-PL	81.7 (72.4, 94.2)	69.5 (63.2, 78.9)	77.1 (67.5, 84.5)	0.0003
HDL1-PL	20.6 (16.8, 25.5)	17.4 (14.5, 20.9)	18.9 (14.8, 22.9)	0.0407
HDL2-PL	13.8 (11.6, 15.9)	12.1 (10.3, 14.3)	13.2 (10.9, 15.4)	0.0956
HDL3-PL	17.9 (16.4, 21.8)	15.8 (14.6, 18.4)	17.1 (15.2, 19.6)	0.0061
HDL4-PL	29.5 (26.0, 32.8)	23.7 (19.2, 28.0)	26.3 (23.1, 30.7)	**0.0002**
HDL-apoA-I	158.0 (147.2, 176.7)	143.5 (134.6, 153.7)	149.2 (138.1, 166.2)	0.0020
HDL1-apoA-I	26.5 (21.0, 31.0)	22.7 (17.9, 27.4)	24.9 (18.5, 30.1)	0.0996
HDL2-apoA-I	18.8 (16.5, 22.2)	16.7 (14.9, 18.6)	17.5 (15.2, 20.6)	0.0275
HDL3-apoA-I	30.3 (25.8, 34.7)	27.2 (24.2, 31.5)	28.8 (24.9, 32.5)	0.1065
HDL4-apoA-I	80.4 (73.5, 90.3)	71.6 (62.0, 80.8)	75.5 (68.3, 86.5)	0.0059
HDL-apoA-II	34.4 (31.5, 38.0)	33.0 (31.4, 36.7)	33.8 (31.5, 37.5)	0.5288
HDL1-apoA-II	2.1 (1.9, 2.9)	2.0 (1.5, 3.3)	2.1 (1.6, 3.0)	0.6088
HDL2-apoA-II	3.6 (3.0, 4.2)	3.7 (3.0, 5.0)	3.7 (3.0, 4.5)	0.5418
HDL3-apoA-II	7.1 (6.2, 8.2)	7.6 (6.4, 8.7)	7.1 (6.3, 8.4)	0.3793
HDL4-apoA-II	20.0 (17.9, 22.7)	18.4 (15.5, 20.8)	19.2 (17.0, 21.9)	0.0440

Data are presented as median (q1, q3). Differences between patients with MS with low and high AOPPs were tested using the Mann-Whitney U test. AOPPs levels below the median (<41.6 μmol/L) were defined as low and those ≥41.6 μmol/L were defined as high AOPPs. Serum levels of lipids and apolipoproteins in HDL are given in mg/dL. *p*-values < 0.0003 are considered statistically significant after a Bonferroni correction for multiple testing and are depicted in bold. AOPPs, advanced oxidation protein products; apoA-I, apolipoprotein A-I; apoA-I, apolipoprotein A-II; C, cholesterol; FC, free cholesterol; HDL, high-density lipoprotein; MS, metabolic syndrome; PL, phospholipid; TG, triglyceride.
