# Supplementary material for: Advanced Oxidation Protein Products Are Strongly Associated with the Serum Levels and Lipid Contents of Lipoprotein Subclasses in Healthy Volunteers and Patients with Metabolic Syndrome
Source: Antioxidants (Basel). 2024 Mar 11;13(3):339. doi: 10.3390/antiox13030339 (PMC10968302; doi:10.3390/antiox13030339)
Supplement: Supplementary file 1 [file antioxidants-13-00339-s001.zip › Table S32.pdf]

Table S32. Differences in the lipid content of total HDL and HDL subclasses between HV with low and high AOPPs.

	HV			
	**Low AOPPs (N=33)**	**High AOPPs (N=32)**	**ALL HV (N=65)**	**p**
HDL-C / HDL-apoA-I	0.41 (0.38, 0.42)	0.37 (0.35, 0.39)	0.39 (0.36, 0.41)	**< 0.0001**
HDL1-C / HDL1-apoA-I	0.61 (0.59, 0.66)	0.68 (0.63, 0.72)	0.64 (0.60, 0.69)	0.0069
HDL2-C / HDL2-apoA-I	0.51 (0.47, 0.54)	0.48 (0.44, 0.50)	0.49 (0.45, 0.53)	0.0369
HDL3-C / HDL3-apoA-I	0.40 (0.39, 0.41)	0.39 (0.38, 0.40)	0.40 (0.38, 0.41)	0.0018
HDL4-C / HDL4-apoA-I	0.27 (0.26, 0.28)	0.26 (0.25, 0.27)	0.26 (0.25, 0.28)	0.0090
HDL-FC / HDL-apoA-I	0.10 (0.10, 0.11)	0.09 (0.08, 0.10)	0.10 (0.09, 0.10)	**< 0.0001**
HDL1-FC / HDL1-apoA-I	0.18 (0.16, 0.20)	0.19 (0.18, 0.22)	0.18 (0.16, 0.21)	0.0257
HDL2-FC / HDL2-apoA-I	0.14 (0.13, 0.14)	0.13 (0.12, 0.14)	0.14 (0.13, 0.14)	0.5906
HDL3-FC / HDL3-apoA-I	0.10 (0.09, 0.10)	0.10 (0.09, 0.10)	0.10 (0.09, 0.10)	0.7528
HDL4-FC / HDL4-apoA-I	0.06 (0.06, 0.06)	0.06 (0.05, 0.06)	0.06 (0.05, 0.06)	0.2999
HDL-TG / HDL-apoA-I	0.05 (0.04, 0.06)	0.07 (0.06, 0.08)	0.06 (0.05, 0.07)	0.0023
HDL1-TG / HDL1-apoA-I	0.09 (0.08, 0.11)	0.13 (0.10, 0.14)	0.11 (0.08, 0.13)	**< 0.0001**
HDL2-TG / HDL2-apoA-I	0.07 (0.06, 0.09)	0.10 (0.08, 0.12)	0.08 (0.07, 0.11)	0.0008
HDL3-TG / HDL3-apoA-I	0.06 (0.05, 0.07)	0.08 (0.07, 0.09)	0.07 (0.06, 0.08)	**< 0.0001**
HDL4-TG / HDL4-apoA-I	0.03 (0.03, 0.04)	0.04 (0.04, 0.05)	0.04 (0.03, 0.05)	**< 0.0001**
HDL-PL / HDL-apoA-I	0.55 (0.52, 0.57)	0.52 (0.49, 0.53)	0.53 (0.50, 0.55)	0.0011
HDL1-PL / HDL1-apoA-I	0.75 (0.72, 0.77)	0.81 (0.76, 0.84)	0.77 (0.74, 0.82)	0.0033
HDL2-PL / HDL2-apoA-I	0.77 (0.70, 0.79)	0.71 (0.66, 0.77)	0.74 (0.68, 0.79)	0.1184
HDL3-PL / HDL3-apoA-I	0.62 (0.61, 0.63)	0.61 (0.60, 0.64)	0.62 (0.60, 0.63)	0.6842
HDL4-PL / HDL4-apoA-I	0.37 (0.36, 0.38)	0.36 (0.34, 0.37)	0.36 (0.35, 0.37)	0.1765

Data are presented as median (q1, q3). Differences between HV with low and high AOPPs were tested using the Mann-Whitney U test. AOPPs levels below the median (<34.6 μmol/L) were defined as low and those equal to ≥34.6 μmol/L were defined as high AOPPs. *p*-values < 0.0003 are considered statistically significant after a Bonferroni correction for multiple testing and are depicted in bold. AOPPs, advanced oxidation protein products; apoA-I, apolipoprotein A-I; C, cholesterol; FC, free cholesterol; HDL, high-density lipoprotein; HV, healthy volunteer; PL, phospholipid; TG, triglyceride.
