# Supplementary material for: Advanced Oxidation Protein Products Are Strongly Associated with the Serum Levels and Lipid Contents of Lipoprotein Subclasses in Healthy Volunteers and Patients with Metabolic Syndrome
Source: Antioxidants (Basel). 2024 Mar 11;13(3):339. doi: 10.3390/antiox13030339 (PMC10968302; doi:10.3390/antiox13030339)
Supplement: Supplementary file 1 [file antioxidants-13-00339-s001.zip › Table S33.pdf]

Table S33. Differences in the lipid content of the total HDL and HDL subclasses between patients with MS with low and high AOPPs.

	MS			
**Variable**	**Low AOPPs (N=33)**	**High AOPPs (N=32)**	**ALL MS (N=65)**	**p**
HDL-C / HDL-apoA-I	0.37 (0.36, 0.39)	0.34 (0.32, 0.36)	0.36 (0.33, 0.38)	**< 0.0001**
HDL1-C / HDL1-apoA-I	0.63 (0.60, 0.71)	0.68 (0.59, 0.74)	0.65 (0.60, 0.72)	0.4466
HDL2-C / HDL2-apoA-I	0.47 (0.45, 0.50)	0.47 (0.42, 0.50)	0.47 (0.43, 0.50)	0.4466
HDL3-C / HDL3-apoA-I	0.39 (0.38, 0.40)	0.36 (0.34, 0.38)	0.38 (0.36, 0.39)	**< 0.0001**
HDL4-C / HDL4-apoA-I	0.26 (0.24, 0.27)	0.23 (0.20, 0.24)	0.24 (0.23, 0.26)	**< 0.0001**
HDL-FC / HDL-apoA-I	0.09 (0.09, 0.10)	0.09 (0.08, 0.09)	0.09 (0.08, 0.10)	0.0209
HDL1-FC / HDL1-apoA-I	0.19 (0.16, 0.20)	0.18 (0.15, 0.22)	0.18 (0.16, 0.20)	0.7231
HDL2-FC / HDL2-apoA-I	0.13 (0.12, 0.14)	0.13 (0.12, 0.15)	0.13 (0.12, 0.14)	0.9686
HDL3-FC / HDL3-apoA-I	0.09 (0.08, 0.10)	0.09 (0.07, 0.10)	0.09 (0.08, 0.10)	0.1381
HDL4-FC / HDL4-apoA-I	0.06 (0.05, 0.06)	0.05 (0.05, 0.06)	0.06 (0.05, 0.06)	0.0571
HDL-TG / HDL-apoA-I	0.06 (0.06, 0.07)	0.09 (0.08, 0.12)	0.08 (0.06, 0.10)	**< 0.0001**
HDL1-TG / HDL1-apoA-I	0.12 (0.10, 0.13)	0.19 (0.16, 0.22)	0.15 (0.11, 0.19)	**< 0.0001**
HDL2-TG / HDL2-apoA-I	0.09 (0.08, 0.11)	0.15 (0.12, 0.18)	0.12 (0.09, 0.16)	**< 0.0001**
HDL3-TG / HDL3-apoA-I	0.07 (0.07, 0.09)	0.12 (0.10, 0.14)	0.09 (0.07, 0.12)	**< 0.0001**
HDL4-TG / HDL4-apoA-I	0.04 (0.04, 0.05)	0.06 (0.05, 0.08)	0.05 (0.04, 0.06)	**< 0.0001**
HDL-PL / HDL-apoA-I	0.52 (0.49, 0.55)	0.49 (0.46, 0.51)	0.51 (0.47, 0.54)	0.0016
HDL1-PL / HDL1-apoA-I	0.78 (0.75, 0.81)	0.79 (0.73, 0.83)	0.78 (0.74, 0.83)	0.5637
HDL2-PL / HDL2-apoA-I	0.74 (0.69, 0.76)	0.73 (0.68, 0.78)	0.74 (0.68, 0.76)	0.6746
HDL3-PL / HDL3-apoA-I	0.62 (0.60, 0.63)	0.60 (0.57, 0.62)	0.61 (0.59, 0.63)	0.0358
HDL4-PL / HDL4-apoA-I	0.36 (0.35, 0.38)	0.33 (0.32, 0.35)	0.35 (0.33, 0.37)	**< 0.0001**

Data are presented as median (q1, q3). Differences between patients with MS with low and high AOPPs were tested using the Mann-Whitney U test. AOPPs levels below the median (<41.6 μmol/L) were defined as low and those ≥41.6 μmol/L were defined as high AOPPs. *p*-values < 0.0003 are considered statistically significant after a Bonferroni correction for multiple testing and are depicted in bold. AOPPs, advanced oxidation protein products; apoA-I, apolipoprotein A-I; C, cholesterol; FC, free cholesterol; HDL, high-density lipoprotein; MS, metabolic syndrome; PL, phospholipid; TG, triglyceride.
