# Supplementary material for: Advanced Oxidation Protein Products Are Strongly Associated with the Serum Levels and Lipid Contents of Lipoprotein Subclasses in Healthy Volunteers and Patients with Metabolic Syndrome
Source: Antioxidants (Basel). 2024 Mar 11;13(3):339. doi: 10.3390/antiox13030339 (PMC10968302; doi:10.3390/antiox13030339)
Supplement: Supplementary file 1 [file antioxidants-13-00339-s001.zip › Table S34.pdf]

Table S34. VIP scores in HV and patients with MS

HV		MS	
Variable	VIP score	Variable	VIP score
LDL-PL/LDL-apoB	1.60	LDL6-PL/LDL6-apoB	1.60
LDL5-TG	1.58	VLDL4-C	1.59
IDL-FC	1.53	VLDL3-C	1.58
IDL-apoB	1.53	LDL5-FC/LDL5-apoB	1.58
IDL-C	1.51	VLDL2-PL	1.57
IDL-PL	1.47	VLDL-PL	1.57
VLDL-apoB	1.47	VLDL3-PL	1.57
LDL-FC/LDL-apoB	1.45	LDL6-FC/LDL6-apoB	1.57
LDL4-PL/LDL4-apoB	1.44	VLDL2-C	1.56
VLDL2-PL	1.43	VLDL4-PL	1.55
VLDL-PL	1.43	IDL-C/IDL-apoB	1.53
VLDL-C	1.41	VLDL-C	1.52
VLDL1-FC	1.40	VLDL4-FC	1.51
VLDL-TG	1.40	VLDL-FC	1.51
VLDL2-C	1.40	VLDL-apoB	1.50
VLDL-FC	1.39	IDL-C	1.49
LDL-TG	1.39	IDL-FC	1.49
LDL5-apoB	1.39	IDL-FC/IDL-apoB	1.48
VLDL3-C	1.38	LDL6-C	1.48
IDL-TG	1.38	VLDL3-FC	1.48
LDL3-PL/LDL3-apoB	1.38	LDL-PL/LDL-apoB	1.47
VLDL3-PL	1.38	IDL-PL	1.47
HDL4-TG	1.38	VLDL2-TG	1.46
VLDL1-PL	1.37	VLDL3-TG	1.46
VLDL4-C	1.37	LDL6-apoB	1.45
VLDL2-TG	1.37	VLDL2-FC	1.45
LDL4-TG	1.37	LDL5-TG	1.45
LDL6-PL/LDL6-apoB	1.35	LDL6-PL	1.43
VLDL4-PL	1.34	VLDL4-TG	1.43
VLDL2-FC	1.33	HDL-C/HDL-apoA-I	1.42
VLDL1-C	1.33	IDL-apoB	1.42
VLDL3-FC	1.33	HDL3-TG/HDL3-apoA-I	1.40
VLDL1-TG	1.33	VLDL1-FC	1.40
VLDL4-FC	1.33	VLDL-C/VLDL-apoB	1.40
HDL2-C	1.32	LDL-FC/LDL-apoB	1.39

HDL-C/HDL-apoA-I	1.32	VLDL-TG	1.37
LDL5-PL	1.31	LDL6-FC	1.36
LDL2-PL/LDL2-apoB	1.31	HDL4-TG	1.36
HDL-C	1.30	VLDL1-PL	1.35
LDL5-C	1.30	VLDL1-C	1.35
LDL-C/LDL-apoB	1.30	HDL1-TG/HDL1-apoA-I	1.33
VLDL3-TG	1.29	HDL2-TG/HDL2-apoA-I	1.32
LDL1-TG/LDL1-apoB	1.29	IDL-TG/IDL-apoB	1.31
LDL5-FC/LDL5-apoB	1.28	LDL2-PL/LDL2-apoB	1.30
HDL4-TG/HDL4-apoA-I	1.26	HDL-C	1.30
LDL6-FC/LDL6-apoB	1.24	VLDL1-TG	1.27
VLDL4-TG	1.24	IDL-TG	1.26
HDL-PL	1.23	LDL-TG	1.19
HDL-FC	1.23	IDL-PL/IDL-apoB	1.19
HDL3-TG/HDL3-apoA-I	1.22	HDL4-C/HDL4-apoA-I	1.19
HDL2-PL	1.20	LDL-C/LDL-apoB	1.18
HDL2-FC	1.19	HDL3-C/HDL3-apoA-I	1.16
LDL6-apoB	1.19	HDL-TG/HDL-apoA-I	1.16
HDL1-FC	1.18	LDL6-TG	1.16
HDL1-TG/HDL1-apoA-I	1.18	HDL-PL	1.14
LDL2-C/LDL2-apoB	1.16	LDL5-PL/LDL5-apoB	1.10
LDL5-FC	1.15	HDL-PL/HDL-apoA-I	1.07
HDL-FC/HDL-apoA-I	1.15	LDL5-apoB	1.06
LDL3-FC/LDL3-apoB	1.14	LDL4-FC/LDL4-apoB	1.05
LDL4-apoB	1.14	HDL3-TG	1.04
HDL1-apoA-I	1.14	HDL4-PL/HDL4-apoA-I	1.03
HDL2-apoA-I	1.13	HDL4-C	1.03
HDL-apoA-I	1.13	LDL1-TG	1.02
HDL1-PL	1.13	HDL4-PL	1.01
HDL1-C	1.12	LDL4-PL/LDL4-apoB	0.98
LDL2-TG/LDL2-apoB	1.10	VLDL5-FC	0.98
LDL6-C	1.08	LDL-apoB	0.97
VLDL-FC/VLDL-apoB	1.05	HDL-TG	0.96
LDL-apoB	1.05	LDL3-PL/LDL3-apoB	0.95
HDL-PL/HDL-apoA-I	1.02	VLDL-TG/VLDL-apoB	0.94
HDL3-TG	0.99	HDL-apoA-I	0.94
LDL4-FC/LDL4-apoB	0.99	LDL5-TG/LDL5-apoB	0.91

HDL1-apoA-II	0.99	HDL-FC	0.89
LDL1-TG	0.98	HDL3-PL/HDL3-apoA-I	0.88
LDL4-PL	0.98	LDL6-C/LDL6-apoB	0.87
LDL4-C/LDL4-apoB	0.97	HDL2-TG	0.87
LDL6-PL	0.97	LDL4-C/LDL4-apoB	0.85
HDL3-C	0.97	HDL3-C	0.85
HDL1-FC/HDL1-apoA-I	0.96	LDL2-TG/LDL2-apoB	0.85
LDL6-TG	0.95	LDL5-C	0.83
HDL1-PL/HDL1-apoA-I	0.94	VLDL5-PL	0.83
LDL4-C	0.94	HDL4-apoA-I	0.82
LDL3-C/LDL3-apoB	0.93	VLDL5-TG	0.78
HDL2-TG/HDL2-apoA-I	0.91	HDL3-PL	0.78
HDL-TG/HDL-apoA-I	0.90	LDL1-PL/LDL1-apoB	0.78
HDL1-C/HDL1-apoA-I	0.87	LDL5-PL	0.77
HDL3-C/HDL3-apoA-I	0.85	HDL1-TG	0.77
HDL2-C/HDL2-apoA-I	0.85	LDL6-TG/LDL6-apoB	0.76
LDL6-FC	0.83	HDL1-PL	0.75
LDL1-PL/LDL1-apoB	0.81	LDL2-C/LDL2-apoB	0.75
IDL-TG/IDL-apoB	0.81	HDL2-FC	0.74
LDL-TG/LDL-apoB	0.78	HDL2-C	0.72
HDL3-apoA-I	0.75	LDL5-C/LDL5-apoB	0.70
IDL-FC/IDL-apoB	0.75	HDL4-FC	0.70
HDL3-PL	0.73	LDL4-TG	0.69
LDL2-FC	0.72	LDL-TG/LDL-apoB	0.69
HDL2-PL/HDL2-apoA-I	0.71	LDL1-TG/LDL1-apoB	0.68
LDL4-FC	0.69	HDL2-apoA-I	0.66
LDL2-C	0.68	LDL3-C/LDL3-apoB	0.66
LDL2-PL	0.68	HDL3-FC	0.65
LDL5-PL/LDL5-apoB	0.68	HDL4-FC/HDL4-apoA-I	0.64
VLDL-PL/VLDL-apoB	0.68	HDL1-apoA-I	0.62
LDL2-FC/LDL2-apoB	0.66	VLDL-FC/VLDL-apoB	0.62
HDL4-C/HDL4-apoA-I	0.65	HDL1-FC	0.62
LDL-PL	0.62	HDL2-PL	0.60
LDL-C	0.61	LDL2-TG	0.60
LDL6-C/LDL6-apoB	0.60	LDL5-FC	0.58
VLDL5-PL	0.60	LDL3-TG/LDL3-apoB	0.56

LDL4-TG/LDL4-apoB	0.58	LDL2-PL	0.55
VLDL5-FC	0.58	HDL1-C	0.54
IDL-C/IDL-apoB	0.55	HDL4-apoA-II	0.52
LDL2-apoB	0.55	HDL4-TG/HDL4-apoA-I	0.50
HDL2-apoA-II	0.54	HDL-FC/HDL-apoA-I	0.47
HDL4-apoA-II	0.54	LDL2-C	0.45
HDL3-FC	0.52	LDL-C	0.44
LDL5-TG/LDL5-apoB	0.51	LDL2-FC	0.44
IDL-PL/IDL-apoB	0.50	HDL3-apoA-II	0.41
LDL1-C/LDL1-apoB	0.50	HDL3-apoA-I	0.40
VLDL-C/VLDL-apoB	0.50	LDL3-PL	0.38
HDL-TG	0.43	HDL2-apoA-II	0.37
LDL-FC	0.42	HDL3-FC/HDL3-apoA-I	0.36
LDL2-TG	0.41	LDL1-C/LDL1-apoB	0.36
LDL3-FC	0.41	HDL1-FC/HDL1-apoA-I	0.34
LDL3-TG/LDL3-apoB	0.39	LDL3-FC	0.32
LDL6-TG/LDL6-apoB	0.38	VLDL5-C	0.32
HDL1-TG	0.38	LDL-PL	0.31
HDL4-PL/HDL4-apoA-I	0.38	LDL3-FC/LDL3-apoB	0.31
VLDL5-C	0.37	LDL3-C	0.31
LDL1-FC/LDL1-apoB	0.34	LDL2-apoB	0.31
VLDL5-TG	0.32	HDL2-C/HDL2-apoA-I	0.30
HDL4-apoA-I	0.29	VLDL-PL/VLDL-apoB	0.27
LDL3-TG	0.28	HDL1-C/HDL1-apoA-I	0.27
HDL3-PL/HDL3-apoA-I	0.27	LDL3-apoB	0.23
LDL5-C/LDL5-apoB	0.25	LDL2-FC/LDL2-apoB	0.23
HDL3-apoA-II	0.25	LDL4-TG/LDL4-apoB	0.19
HDL2-TG	0.25	LDL1-FC/LDL1-apoB	0.17
VLDL-TG/VLDL-apoB	0.22	LDL1-FC	0.17
HDL2-FC/HDL2-apoA-I	0.22	LDL4-apoB	0.17
LDL1-apoB	0.16	LDL4-FC	0.17
HDL4-FC	0.14	LDL1-apoB	0.15
LDL3-C	0.14	LDL-FC	0.15
LDL3-PL	0.13	HDL1-PL/HDL1-apoA-I	0.13
HDL4-PL	0.12	HDL-apoA-II	0.11
HDL4-FC/HDL4-apoA-I	0.12	HDL1-apoA-II	0.11

LDL1-PL	0.09	HDL2-FC/HDL2-apoA-I	0.09
LDL1-C	0.09	LDL3-TG	0.09
LDL1-FC	0.09	LDL1-C	0.08
HDL3-FC/HDL3-apoA-I	0.07	HDL2-PL/HDL2-apoA-I	0.05
LDL3-apoB	0.03	LDL4-PL	0.02
HDL4-C	0.03	LDL1-PL	0.01
HDL-apoA-II	0.03	LDL4-C	0.01
