# Supplementary material for: Advanced Oxidation Protein Products Are Strongly Associated with the Serum Levels and Lipid Contents of Lipoprotein Subclasses in Healthy Volunteers and Patients with Metabolic Syndrome
Source: Antioxidants (Basel). 2024 Mar 11;13(3):339. doi: 10.3390/antiox13030339 (PMC10968302; doi:10.3390/antiox13030339)
Supplement: Supplementary file 1 [file antioxidants-13-00339-s001.zip › Table S4.pdf]

Table S4. Differences in serum levels and lipid content of IDL particles between HV and patients with MS.

Variable	All (N=130)	HV (N=65)	MS (N=65)	p
IDL-C	15.9 (11.1, 21.8)	14.5 (10.0, 18.9)	18.3 (13.3, 26.6)	0.0006
IDL-FC	4.6 (3.1, 6.2)	4.0 (2.9, 5.4)	5.3 (3.8, 7.8)	0.0013
IDL-TG	9.4 (5.5, 18.2)	7.1 (4.1, 11.6)	13.8 (7.3, 22.5)	< **0.0001**
IDL-PL	7.8 (6.2, 11.1)	7.3 (6.1, 9.4)	9.4 (6.5, 12.7)	0.0199
IDL-apoB	6.0 (4.5, 7.8)	5.4 (4.0, 6.9)	6.9 (5.1, 9.0)	< **0.0001**
IDL-C/IDL-apoB	2.69 (2.45, 2.91)	2.68 (2.49, 2.89)	2.75 (2.45, 2.94)	0.9870
IDL-FC/IDL-apoB	0.76 (0.69, 0.83)	0.76 (0.70, 0.82)	0.76 (0.66, 0.84)	0.7675
IDL-TG/IDL-apoB	1.70 (1.05, 2.41)	1.41 (0.91, 2.06)	1.97 (1.25, 2.72)	0.0035
IDL-PL/IDL-apoB	1.39 (1.22, 1.59)	1.42 (1.31, 1.63)	1.35 (1.12, 1.54)	0.0048

Data are presented as median (q1, q3). Differences between HV and patients with MS were tested using the Mann-Whitney U test. Serum levels of IDL components are given in mg/dL. *p*-values < 0.0003 are considered statistically significant and are depicted in bold. apoB, apolipoprotein B; C, cholesterol; FC, free cholesterol; HV, healthy volunteer; IDL, intermediate-density lipoprotein; MS, metabolic syndrome patient; N, number; PL, phospholipid; TG, triglyceride.
