# Supplementary material for: Advanced Oxidation Protein Products Are Strongly Associated with the Serum Levels and Lipid Contents of Lipoprotein Subclasses in Healthy Volunteers and Patients with Metabolic Syndrome
Source: Antioxidants (Basel). 2024 Mar 11;13(3):339. doi: 10.3390/antiox13030339 (PMC10968302; doi:10.3390/antiox13030339)
Supplement: Supplementary file 1 [file antioxidants-13-00339-s001.zip › Table S5.pdf]

Table S5. Differences in serum levels of total LDL and LDL subclasses between HV and patients with MS.

Variable (mg/dL)	All (N=130)	HV (N=65)	MS (N=65)	p
LDL-C	125.1 (113.0, 153.1)	137.4 (121.8, 154.3)	117.1 (90.4, 148.1)	0.0006
LDL1-C	28.1 (23.4, 34.6)	30.4 (26.5, 36.7)	24.5 (21.3, 30.1)	< **0.0001**
LDL2-C	21.6 (14.8, 26.6)	25.9 (20.1, 30.7)	17.4 (12.2, 22.3)	< **0.0001**
LDL3-C	21.4 (15.4, 27.6)	23.8 (21.0, 29.1)	17.6 (9.9, 22.2)	< **0.0001**
LDL4-C	19.2 (14.2, 24.2)	20.3 (15.8, 24.4)	17.9 (11.2, 24.1)	0.0519
LDL5-C	16.5 (12.7, 22.3)	15.9 (12.4, 21.9)	17.4 (13.1, 23.8)	0.2434
LDL6-C	20.1 (16.6, 25.8)	19.2 (16.5, 22.8)	22.1 (17.1, 28.8)	0.0387
LDL-FC	38.7 (35.2, 46.3)	42.2 (38.0, 47.3)	37.3 (28.8, 45.1)	0.0011
LDL1-FC	9.0 (7.7, 10.9)	9.5 (8.4, 11.3)	8.2 (7.1, 9.8)	0.0006
LDL2-FC	7.5 (5.3, 8.9)	8.6 (6.7, 9.8)	6.4 (4.9, 7.7)	< **0.0001**
LDL3-FC	7.2 (5.5, 8.6)	7.9 (7.1, 9.2)	6.5 (4.3, 7.3)	< **0.0001**
LDL4-FC	6.2 (4.9, 7.4)	6.6 (5.3, 7.6)	5.8 (4.5, 7.1)	0.0463
LDL5-FC	5.3 (4.4, 6.7)	5.3 (4.2, 6.5)	5.3 (4.6, 6.8)	0.2549
LDL6-FC	6.1 (5.1, 7.1)	5.8 (4.9, 6.5)	6.4 (5.3, 7.6)	0.0226
LDL-TG	21.7 (18.1, 25.9)	20.0 (16.5, 23.6)	23.8 (20.5, 28.3)	< **0.0001**
LDL1-TG	6.8 (5.7, 8.2)	6.4 (5.1, 7.6)	7.5 (6.3, 8.6)	0.0008
LDL2-TG	2.6 (2.1, 3.2)	2.7 (2.1, 3.2)	2.6 (2.1, 3.2)	0.5220
LDL3-TG	2.5 (1.9, 3.0)	2.5 (2.1, 3.0)	2.4 (1.9, 3.0)	0.3663
LDL4-TG	2.7 (1.9, 3.4)	2.5 (1.6, 3.0)	2.9 (2.1, 3.6)	0.0026
LDL5-TG	2.5 (1.8, 3.6)	2.0 (1.4, 3.0)	3.1 (2.2, 4.3)	< **0.0001**
LDL6-TG	3.2 (2.6, 4.1)	2.8 (2.3, 3.4)	3.9 (2.9, 4.7)	< **0.0001**
LDL-PL	70.9 (63.5, 83.3)	75.9 (67.9, 85.4)	66.6 (51.5, 80.2)	0.0015
LDL1-PL	15.5 (13.3, 18.7)	16.6 (14.7, 19.7)	14.3 (12.1, 16.9)	0.0005
LDL2-PL	12.1 (8.4, 14.5)	14.0 (11.1, 16.2)	9.6 (7.0, 12.4)	< **0.0001**
LDL3-PL	11.9 (8.9, 15.0)	13.2 (11.3, 15.6)	10.1 (6.3, 12.4)	< **0.0001**
LDL4-PL	10.4 (8.0, 13.0)	11.4 (9.0, 13.1)	10.2 (6.7, 12.9)	0.1051
LDL5-PL	9.2 (7.3, 12.0)	8.7 (7.0, 11.8)	9.6 (7.5, 12.6)	0.1638
LDL6-PL	11.6 (10.1, 14.3)	11.2 (10.0, 12.8)	12.6 (10.2, 15.3)	0.0291
LDL-apoB	81.7 (70.0, 94.6)	82.3 (72.8, 94.9)	78.5 (65.0, 92.1)	0.2648
LDL1-apoB	14.7 (12.7, 17.8)	15.3 (13.4, 18.4)	13.9 (11.5, 16.3)	0.0046
LDL2-apoB	12.3 (8.9, 14.7)	13.8 (11.2, 16.4)	9.9 (7.3, 12.8)	< **0.0001**
LDL3-apoB	12.5 (9.8, 15.6)	13.7 (12.0, 16.4)	10.8 (6.9, 13.2)	< **0.0001**
LDL4-apoB	12.4 (9.4, 15.7)	13.4 (9.7, 15.3)	12.3 (8.4, 16.2)	0.4409
LDL5-apoB	11.9 (9.2, 16.2)	11.3 (8.4, 15.2)	12.8 (9.9, 17.7)	0.0306
LDL6-apoB	16.2 (13.6, 22.0)	15.8 (13.4, 18.0)	18.0 (14.1, 23.9)	0.0138

Data are presented as median (q1, q3). Differences between HV and patients with MS were tested using the Mann-Whitney U test. *p*-values < 0.0003 are considered statistically significant and are depicted in bold. ApoB, apolipoprotein B; C, cholesterol; FC, free cholesterol; HV, healthy volunteer; LDL, low-density lipoprotein; MS, metabolic syndrome patient; N, number; PL, phospholipid; TG, triglyceride.
