# Supplementary material for: Advanced Oxidation Protein Products Are Strongly Associated with the Serum Levels and Lipid Contents of Lipoprotein Subclasses in Healthy Volunteers and Patients with Metabolic Syndrome
Source: Antioxidants (Basel). 2024 Mar 11;13(3):339. doi: 10.3390/antiox13030339 (PMC10968302; doi:10.3390/antiox13030339)
Supplement: Supplementary file 1 [file antioxidants-13-00339-s001.zip › Table S7.pdf]

Table S7. Differences in serum levels of HDL subclasses between HV and patients with MS.

Variable (mg/dL)	**All (N=130)**	**HV (N=65)**	**MS (N=65)**	p
HDL-C	58.6 (51.3, 69.2)	65.2 (57.7, 74.5)	52.7 (47.9, 60.6)	< **0.0001**
HDL1-C	17.2 (13.8, 22.5)	18.4 (15.1, 26.8)	15.7 (12.9, 20.1)	0.0011
HDL2-C	8.6 (7.5, 10.2)	9.5 (8.3, 12.6)	8.2 (7.3, 9.7)	0.0012
HDL3-C	11.2 (10.0, 13.2)	12.2 (10.7, 13.6)	10.4 (9.6, 11.9)	**0.0001**
HDL4-C	20.5 (17.2, 23.9)	22.3 (18.2, 24.7)	19.0 (16.0, 22.7)	0.0013
HDL-FC	14.5 (12.5, 16.7)	16.0 (14.1, 18.9)	13.5 (11.8, 15.0)	< **0.0001**
HDL1-FC	5.0 (4.1, 6.1)	5.4 (4.8, 7.4)	4.3 (3.7, 5.2)	< **0.0001**
HDL2-FC	2.5 (2.1, 2.9)	2.7 (2.4, 3.1)	2.3 (2.0, 2.7)	0.0015
HDL3-FC	2.8 (2.3, 3.2)	3.0 (2.6, 3.4)	2.5 (2.1, 3.0)	0.0012
HDL4-FC	4.6 (3.9, 5.4)	4.9 (4.0, 5.7)	4.4 (3.8, 5.2)	0.0326
HDL-TG	10.5 (9.0, 13.3)	9.9 (8.7, 11.8)	11.4 (9.7, 13.6)	0.0058
HDL1-TG	3.3 (2.6, 4.4)	3.0 (2.5, 4.2)	3.6 (2.7, 4.6)	0.3258
HDL2-TG	1.8 (1.5, 2.3)	1.6 (1.3, 2.1)	2.0 (1.6, 2.5)	0.0013
HDL3-TG	2.3 (1.9, 2.8)	2.1 (1.7, 2.5)	2.7 (2.1, 3.1)	**0.0002**
HDL4-TG	3.6 (3.0, 4.3)	3.4 (2.5, 3.9)	3.7 (3.3, 4.7)	0.0003
HDL-PL	81.9 (72.1, 93.2)	89.4 (79.2, 99.8)	77.1 (67.5, 84.5)	< **0.0001**
HDL1-PL	20.5 (16.8, 26.5)	22.1 (18.6, 33.9)	18.9 (14.8, 22.9)	0.0021
HDL2-PL	13.8 (11.7, 15.9)	14.4 (12.6, 18.6)	13.2 (10.9, 15.4)	0.0175
HDL3-PL	18.2 (15.9, 20.6)	19.1 (17.4, 20.9)	17.1 (15.2, 19.6)	0.0037
HDL4-PL	28.8 (25.4, 31.8)	29.9 (26.4, 32.7)	26.3 (23.1, 30.7)	0.0033
HDL-apoA-I	159.3 (144.5, 178.2)	167.8 (155.7, 183.7)	149.2 (138.1, 166.2)	< **0.0001**
HDL1-apoA-I	26.3 (20.0, 34.7)	27.5 (22.3, 45.4)	24.9 (18.5, 30.1)	0.0067
HDL2-apoA-I	18.7 (16.3, 22.1)	19.6 (17.2, 23.3)	17.5 (15.2, 20.6)	0.0036
HDL3-apoA-I	30.2 (26.7, 33.3)	30.8 (27.5, 33.5)	28.8 (24.9, 32.5)	0.0396
HDL4-apoA-I	79.5 (70.8, 89.0)	81.9 (72.7, 91.1)	75.5 (68.3, 86.5)	0.0231
HDL-apoA-II	35.2 (32.2, 38.4)	36.0 (33.6, 38.6)	33.8 (31.5, 37.5)	0.0389
HDL1-apoA-II	2.4 (1.9, 3.5)	2.5 (2.1, 4.1)	2.1 (1.6, 3.0)	0.0058
HDL2-apoA-II	3.9 (3.2, 4.6)	4.0 (3.5, 4.6)	3.7 (3.0, 4.5)	0.1107
HDL3-apoA-II	7.3 (6.5, 8.2)	7.3 (6.9, 8.1)	7.1 (6.3, 8.4)	0.7392
HDL4-apoA-II	19.8 (17.5, 22.6)	20.9 (18.4, 23.2)	19.2 (17.0, 21.9)	0.0600

Data are presented as median (q1, q3). Differences between HV and patients with MS were tested using the Mann-Whitney U test. p-values < 0.0003 are considered statistically significant and are depicted in bold. ApoA-I, apolipoprotein A-I; apoA-II, apolipoprotein A-II; C, cholesterol; FC, free cholesterol; HV, healthy volunteer; HDL, high-density lipoprotein; MS, metabolic syndrome patient; N, number; PL, phospholipid; TG, triglyceride.
