# Supplementary material for: Advanced Oxidation Protein Products Are Strongly Associated with the Serum Levels and Lipid Contents of Lipoprotein Subclasses in Healthy Volunteers and Patients with Metabolic Syndrome
Source: Antioxidants (Basel). 2024 Mar 11;13(3):339. doi: 10.3390/antiox13030339 (PMC10968302; doi:10.3390/antiox13030339)
Supplement: Supplementary file 1 [file antioxidants-13-00339-s001.zip › Table S8.pdf]

Table S8. Differences in the lipid content of HDL particles between HV and patients with MS.

Variable	All (N=130)	HV (N=65)	MS (N=65)	p
HDL-C / HDL-apoA-I	0.37 (0.35, 0.40)	0.39 (0.36, 0.41)	0.36 (0.33, 0.38)	< **0.0001**
HDL1-C / HDL1-apoA-I	0.65 (0.60, 0.71)	0.64 (0.60, 0.69)	0.65 (0.60, 0.72)	0.8687
HDL2-C / HDL2-apoA-I	0.48 (0.45, 0.51)	0.49 (0.45, 0.53)	0.47 (0.43, 0.50)	0.0105
HDL3-C / HDL3-apoA-I	0.39 (0.37, 0.40)	0.40 (0.38, 0.41)	0.38 (0.36, 0.39)	< **0.0001**
HDL4-C / HDL4-apoA-I	0.26 (0.24, 0.27)	0.26 (0.25, 0.28)	0.24 (0.23, 0.26)	< **0.0001**
HDL-FC / HDL-apoA-I	0.09 (0.08, 0.10)	0.10 (0.09, 0.10)	0.09 (0.08, 0.10)	0.0018
HDL1-FC / HDL1-apoA-I	0.18 (0.16, 0.21)	0.18 (0.16, 0.21)	0.18 (0.16, 0.20)	0.5939
HDL2-FC / HDL2-apoA-I	0.13 (0.12, 0.14)	0.14 (0.13, 0.14)	0.13 (0.12, 0.14)	0.5342
HDL3-FC / HDL3-apoA-I	0.09 (0.08, 0.10)	0.10 (0.09, 0.10)	0.09 (0.08, 0.10)	0.0055
HDL4-FC / HDL4-apoA-I	0.06 (0.05, 0.06)	0.06 (0.05, 0.06)	0.06 (0.05, 0.06)	0.1660
HDL-TG / HDL-apoA-I	0.06 (0.06, 0.08)	0.06 (0.05, 0.07)	0.08 (0.06, 0.10)	< **0.0001**
HDL1-TG / HDL1-apoA-I	0.12 (0.09, 0.17)	0.11 (0.08, 0.13)	0.15 (0.11, 0.19)	< **0.0001**
HDL2-TG / HDL2-apoA-I	0.10 (0.08, 0.13)	0.08 (0.07, 0.11)	0.12 (0.09, 0.16)	< **0.0001**
HDL3-TG / HDL3-apoA-I	0.08 (0.06, 0.10)	0.07 (0.06, 0.08)	0.09 (0.07, 0.12)	< **0.0001**
HDL4-TG / HDL4-apoA-I	0.04 (0.04, 0.05)	0.04 (0.03, 0.05)	0.05 (0.04, 0.06)	< **0.0001**
HDL-PL / HDL-apoA-I	0.52 (0.49, 0.54)	0.53 (0.50, 0.55)	0.51 (0.47, 0.54)	0.0024
HDL1-PL / HDL1-apoA-I	0.78 (0.74, 0.83)	0.77 (0.74, 0.82)	0.78 (0.74, 0.83)	0.4605
HDL2-PL / HDL2-apoA-I	0.74 (0.68, 0.78)	0.74 (0.68, 0.79)	0.74 (0.68, 0.76)	0.6940
HDL3-PL / HDL3-apoA-I	0.61 (0.60, 0.63)	0.62 (0.60, 0.63)	0.61 (0.59, 0.63)	0.1450
HDL4-PL / HDL4-apoA-I	0.36 (0.34, 0.37)	0.36 (0.35, 0.37)	0.35 (0.33, 0.37)	0.0015

Data are presented as median (q1, q3). Differences between HV and patients with MS were tested with the Mann-Whitney U test. p-values < 0.0003 are considered statistically significant and are depicted in bold. ApoA-I, apolipoprotein A-I; C, cholesterol; HV, healthy volunteer; HDL, high-density lipoprotein; MS, metabolic syndrome patient; PL, phospholipid; TG, triglyceride.
