# Supplementary material for: Advanced Oxidation Protein Products Are Strongly Associated with the Serum Levels and Lipid Contents of Lipoprotein Subclasses in Healthy Volunteers and Patients with Metabolic Syndrome
Source: Antioxidants (Basel). 2024 Mar 11;13(3):339. doi: 10.3390/antiox13030339 (PMC10968302; doi:10.3390/antiox13030339)
Supplement: Supplementary file 1 [file antioxidants-13-00339-s001.zip › Table S9.pdf]

Table S9. Correlation analyses of AOPPs with the serum levels and lipid content of VLDL, separately in HV and patients with MS.

Variable	AOPPs (μmol/L)			
	HV (N=65)		MS (N=65)	
	r	p	r	p
VLDL-C	0.68	**<0.0001**	0.79	**<0.0001**
VLDL1-C	0.64	**<0.0001**	0.78	**<0.0001**
VLDL2-C	0.66	**<0.0001**	0.79	**<0.0001**
VLDL3-C	0.70	**<0.0001**	0.81	**<0.0001**
VLDL4-C	0.68	**<0.0001**	0.74	**<0.0001**
VLDL5-C	0.12	0.3597	0.02	0.8899
VLDL-FC	0.68	**<0.0001**	0.80	**<0.0001**
VLDL1-FC	0.70	**<0.0001**	0.78	**<0.0001**
VLDL2-FC	0.66	**<0.0001**	0.80	**<0.0001**
VLDL3-FC	0.67	**<0.0001**	0.83	**<0.0001**
VLDL4-FC	0.65	**<0.0001**	0.74	**<0.0001**
VLDL5-FC	0.24	0.0551	0.42	0.0005
VLDL-TG	0.71	**<0.0001**	0.82	**<0.0001**
VLDL1-TG	0.66	**<0.0001**	0.79	**<0.0001**
VLDL2-TG	0.65	**<0.0001**	0.80	**<0.0001**
VLDL3-TG	0.65	**<0.0001**	0.81	**<0.0001**
VLDL4-TG	0.63	**<0.0001**	0.76	**<0.0001**
VLDL5-TG	0.10	0.4077	0.27	0.0325
VLDL-PL	0.69	**<0.0001**	0.80	**<0.0001**
VLDL1-PL	0.66	**<0.0001**	0.80	**<0.0001**
VLDL2-PL	0.68	**<0.0001**	0.82	**<0.0001**
VLDL3-PL	0.68	**<0.0001**	0.82	**<0.0001**
VLDL4-PL	0.66	**<0.0001**	0.75	**<0.0001**
VLDL5-PL	0.27	0.0300	0.27	0.0328
VLDL-apoB	0.69	**<0.0001**	0.82	**<0.0001**
VLDL-C/VLDL-apoB	0.34	0.0057	0.60	**<0.0001**
VLDL-FC/VLDL-apoB	-0.39	0.0013	0.32	0.0103
VLDL-TG/VLDL-apoB	0.08	0.5248	0.47	**<0.0001**
VLDL-PL/VLDL-apoB	-0.21	0.0892	0.03	0.8327

Spearman correlation analyses were used to evaluate the associations of the serum levels of AOPPs with the serum levels and lipid content of VLDL. p-values < 0.0003 are considered statistically significant after a Bonferroni correction for multiple testing and are depicted in bold. Serum levels of lipids and apoB in VLDL are given in mg/dL. AOPPs, advanced oxidation protein products; apoB, apolipoprotein B; C, cholesterol; FC, free cholesterol; HV, healthy volunteer; MS, metabolic syndrome patient; PL, phospholipid; r, Spearman's correlation coefficient; TG, triglyceride, VLDL, very low-density lipoprotein.
